# A Novel Pathosystem With the Model Plant *Arabidopsis thaliana* for Defining the Molecular Basis of *Taphrina* Infections

**DOI:** 10.1111/1758-2229.70118

**Published:** 2025-06-10

**Authors:** Agate Auzane, Margaretta Christita, Kai Wang, Timo Sipilä, Sitaram Rajaraman, Gugan Eswaran, Jasmin Kemppinen, Alejandro De La Fuente, Klaas Bouwmeester, Petri Auvinen, Lars Paulin, Jarkko Salojärvi, Maija Sierla, Mikael Broché, Kirk Overmyer

**Affiliations:** ^1^ Organismal and Evolutionary Biology Research Program, Faculty of Biological and Environmental Sciences, and Viikki Plant Science Centre University of Helsinki Helsinki Finland; ^2^ Biosystematics Group Wageningen University & Research Wageningen the Netherlands; ^3^ Institute of Biotechnology University of Helsinki Helsinki Finland

**Keywords:** *Arabidopsis*, effectors, genomics, receptors, reverse genetics, *Taphrina*, yeast cell wall

## Abstract

Plant‐associated yeasts modulate host immunity to promote or prevent disease. Mechanisms of yeast perception by the plant innate immune system remain unknown, with progress hindered by lack of a model system with the model plant *Arabidopsis thaliana* (*Arabidopsis*). A yeast strain of *Taphrina tormentillae*, named M11, was previously isolated from wild *Arabidopsis. Taphrina* have been found on many non‐host plants, and their complex ecology remains understudied. Here, the interaction of M11 with *Arabidopsis* was characterised. Infection of *Arabidopsis* with the birch pathogen *T. betulina*, used as a non‐host control, triggered typical defence activation features but did not multiply, demonstrating *Arabidopsis* had immunity against a non‐adapted yeast. M11 triggered attenuated defence activation features, grew *in planta*, and caused subtle but clear leaf deformation symptoms, demonstrating it is pathogenic. M11 was widely distributed in environmental sequencing data and found on multiple non‐host plants, suggesting *Taphrina* play previously unrecognised ecological roles on multiple plant species. M11 genome features involved in host interaction were analysed, and potential immune stimulatory molecules in chitin‐free cell walls were identified. A pilot screen demonstrated the utility of reverse genetics with *Arabidopsis* and identified that the BAK1 co‐receptor is involved in the perception of M11 *Taphrina* cell walls.

## Graphical abstract

A *Taphrina* strain, identified as *Taphrina tormentillae,* was shown to cause disease symptoms on the genetic model plant *Arabidopsis thaliana* , from which it was isolated. A genetic approach was used to implicate the known co‐receptor BAK1 in the activation of immune responses by likely PAMPs found in *T. tormentillae* cell walls. The distribution of this and other *Taphrina* species on plants other than their known hosts challenges the practice of naming *Taphrina* species for the plant from which they were isolated.

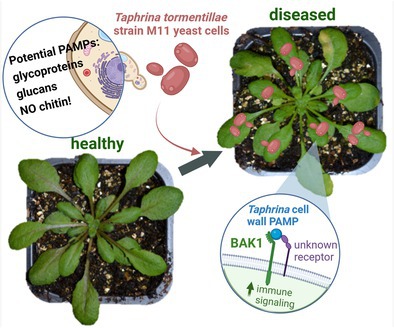

## 1 Introduction

Pathogens are adapted to overcome host‐specific and non‐host resistance mechanisms utilised by the host plant (Dodds and Rathjen 2010; Pieterse et al. 2012; Cui et al. 2015; Panstruga and Moscou 2020). Plants possess a two‐tiered innate‐immune system (Jones and Dangl 2006). The first level is termed basal or non‐host resistance and consists of multiple mechanisms (Lee et al. 2017), including pattern recognition receptors, which recognise conserved pathogen associated molecular patterns (PAMPs) to induce PAMP‐triggered immunity (PTI) (Jones and Dangl 2006). To overcome PTI, adapted pathogens have evolved effectors, which interfere with plant immune signalling. To counter this, plants deploy nucleotide‐binding leucine‐rich repeat immune receptors, which activate immune signalling pathways and downstream responses, termed effector‐triggered immunity (ETI).

Human pathogenic yeasts such as *Candida albicans* have been important systems for understanding yeast‐host interactions and have resulted in the definition of human immune receptors responsible for detecting pathogenic yeasts (Jouault et al. 2009). Progress with plant‐yeast interactions lags behind. There are few known phytopathogenic species among the true yeasts in subphylum Saccharomycotina, with a few examples in the genus *Eremothecium* (Wendland and Walther 2011). However, there are a large number of phytopathogenic yeast‐like fungi with dimorphic lifecycles (hereafter referred to as yeasts), for instance in the Ascomycota subphylum Taphrinomycotina and the Basidiomycota subphylum Ustilaginomycotina (Begerow et al. 2017; Lachance and Walker 2018).

Yeasts from the *Arabidopsis* phyllosphere were previously isolated, including the M11 strain of *Taphrina* (Wang et al. 2016). Species belonging to the genus *Taphrina* (Ascomycota, Taphrinomycotina, Taphrinomycetes, Taphrinales, Taphrinaceae) are little studied pathogens of primarily woody plant species, although some *Taphrina* have herbaceous host species, including, for example, *Curcuma*, *Potentilla*, and some ferns (Mix 1949; Ahmed and Kulkarni 1968; Rodrigues and Fonseca 2003; Fonseca and Rodrigues 2011). Most *Taphrina* cause tumour‐like symptoms on their hosts (Mix 1949; Kern and Naef‐Roth 1975; Bacigálová et al. 2005; Fonseca and Rodrigues 2011). *Protomyces* is the sister genus to *Taphrina*; members of this genus are pathogenic on herbaceous hosts mostly in the families Umbelliferae and Compositae and have similar lifecycles and virulence strategies to the *Taphrina* (Wang et al. 2021). *Taphrina* and *Protomyces* species also share some similarities with *Pneumocystis* species, which are yeast pathogens of mammals; all of these pathogenic genera are members of the Taphrinomycotina (Ma et al. 2016). *Taphrina* species are biotrophic pathogens and invade the intercellular spaces of leaves and woody tissues, just below the epidermis (Mix 1949). Host defence pathways against *Taphrina* species remain poorly defined, and for most *Taphrina* species, there is little to no information available about defined pairs of virulent strains and susceptible/resistant host genotypes. The best studied system is the interaction of *T. deformans* with its host peach, where molecular and metabolic studies have implicated pathogenesis‐related proteins, the plant hormone salicylic acid, and chlorogenic acid in peach resistance (Goldy et al. 2017; Svetaz et al. 2017). *Taphrina* are dimorphic, with a dikaryotic infectious filamentous phase, which invades host tissues, and an easy to culture haploid yeast phase, which resides in the phyllosphere of the host between infection cycles. Infections do not necessarily occur on a yearly basis, and it is held that *Taphrina* can survive as a yeast in the host phyllosphere indefinitely (Mix 1949). This trait makes *Taphrina* species facultative parasites that behave like opportunistic pathogens. Most *Taphrina* isolates were isolated from their respective hosts in a diseased state (Mix 1949). In recent environmental sequencing studies, *Taphrina* have often been found on plants other than their hosts and in a variety of different ecological roles. Some *Taphrina* have been isolated in their yeast states from inert surfaces or plants without disease symptoms, suggesting that some *Taphrina* may be specialised in atypical lifestyles, such as endoliths or non‐pathogenic phyllosphere residents (Moore 1998; Inacio et al. 2004; Selbmann et al. 2014). Much of the previous work on *Taphrina* species is quite old; however, recent genome sequencing projects have opened this genus to modern molecular approaches (Cissé et al. 2013; Tsai et al. 2014; Wang et al. 2020). Members of the genus *Taphrina*, including the M11 strain (Wang et al. 2016), are known for the ability to produce plant hormones auxin and cytokinin. Auxin biosynthesis pathways in *Taphrina* and *Protomyces* species have been addressed in several studies (Cissé et al. 2013; Tsai et al. 2014; Wang, Sipilä, et al. 2019). In spite of recent advances, the complex biology and ecology of these plant‐associated fungi with dual lifestyles remain understudied.

*Arabidopsis thaliana* (referred to hereafter as *Arabidopsis*) is a genetic model plant; the use of this model has facilitated the definition of the plant innate immune system. While receptors involved in immunity against other pathogen classes are well defined, those detecting yeasts remain unknown. Investigation of yeast‐plant interactions utilising *Arabidopsis* has been hindered by a lack of *Arabidopsis‐associated* yeasts. The availability of *Taphrina* strains from wild *Arabidopsis* (Wang et al. 2016) now opens this possibility. Previous studies have used *T. betulina* to study the non‐host interaction with *Arabidopsis* (Gehrmann 2013) and *Protomyces arabidopsidicola* to probe *Arabidopsis* immune activation by a phyllosphere resident yeast (Wang, Huang, et al. 2019; Wang et al. 2021).

*Arabidopsis* has not been previously known to be a host for *Taphrina*. This study aims to describe *Taphrina* strain M11 in comparison to related *Taphrina* species, define its interaction with *Arabidopsis*, identify potential mechanisms of interaction with *Arabidopsis* immune signalling, and explore its distribution and ecology.

## 2 Experimental Procedures

### 2.1 Plants and Cultivation Conditions

*Arabidopsis* ( *Arabidopsis thaliana* ) seeds were obtained from the Nottingham Arabidopsis Stock Centre (NASC; http://arabidopsis.info/) or as indicated. All genotypes used were based on the Col‐0 accession. The primers used for genotyping mutants are listed in Table S2A.

Standard plant cultivation conditions were as follows. For soil‐grown plants, seeds were sown on a well‐watered 1:1 mix of peat (Kekkilä; www.kekkila.fi) and vermiculite, stratified in the dark at 4°C for 72 h, then grown in chambers (Fitotron SGC120, Weiss Technik; www.weiss‐technik.com) at +23°C/+18°C, 65/75% relative humidity, ~170 μmol m^−2^ s^−1^ illumination, and a 12/12 h (light/dark) or constant temperature (23°C), constant humidity (ca. 60%), 150 μmol m^−2^ s^−1^ illumination, and an 8/16 h photoperiod, as indicated. For sterile plant cultivation, seeds were sterilised with chlorine gas for 5 h, sown on 0.5 × MS 0.8% agar, and stratified in the dark at 4°C for 72 h. One‐week‐old seedlings were transplanted into six‐well plates with 4 mL of 0.5 × MS 0.8% agar. The medium and roots were separated from the shoots using tight‐fitting polypropylene disks with a 4 mm hole in the middle. Plants were grown in Fitotron SGC120 growth chambers with a 12/12 h light/dark cycle at ~170 μmol m^−2^ s^−1^ light, +23°C/+18°C, and 65/75% relative humidity.

### 2.2 Microbial Strains and Culture

*Taphrina* strain M11 used here (Wang et al. 2016) has been deposited in the HAMBI‐Helsinki Microbial Domain Biological Resource Centre—under the accession number HAMBI: H3698 and in the DSMZ—The German Collection of Microorganisms and Cell Cultures—under the accession number DSM 110146. All other *Taphrina* strains were obtained from the Portuguese yeast culture collection (PYCC; https://pycc.bio‐aware.com/). *T. betulina* (strain PYCC 5889 = CBS 119536) is not adapted to *Arabidopsis* and was used as a non‐host response control. Two *T. tormentillae* strains (strain CBS 332.55 = PYCC 5705 and strain PYCC 5727) are the strains most closely related to M11. *T. tormentillae* strain PYCC 5705/CBS 332.55 (formerly named *T. carnea* ) was originally isolated from birch and thought to be a birch pathogen, but later shown to be conspecific with *T. tormentillae* (Fonseca and Rodrigues 2011). All yeast were grown on 0.2× potato dextrose agar (PDA) made with 15 g/L agar in potato dextrose broth (PDB; BD Biosciences; https://www.bd.com). *Pseudomonas syringae* pv. tomato strain DC3000 transgenically bearing the *AvrB* avirulence gene (*Pst* DC3000 AvrB) was cultured in NYGA media.

### 2.3 *Arabidopsis* Infections

For soil grown plants, plants were grown with an 8/16 h photoperiod and seven‐day‐old *Taphrina* cells were collected using an inoculation loop, washed in 2 mL 10 mM MgCl_2_ and resuspended in the same at OD_600_ = 0.3. Prior to hand infiltrations, plants were kept at high humidity for 30–60 min to open the stomata. Leaf halves from four‐week‐old soil‐grown *Arabidopsis* were hand infiltrated with yeast suspensions using a needleless syringe, then returned to standard growth conditions, covered with a transparent plastic lid to maintain high humidity for the first 24 h. Similarly prepared suspensions (OD = 0.1) from a one‐day‐old culture of *Pst* DC3000 *AvrB* were used as a positive control for HR induction. Mock treatments with 10 mM MgCl_2_ were used as a negative control in all infection experiments.

For sterile plants, freshly grown cells of all strains were harvested, washed, and suspended in 10 mM MgCl_2_ with 0.04% wetting agent Silwet L77. Yeast suspensions (OD_600_ = 0.02) and control solution were applied onto 24‐day‐old plants using sterile plastic spray bottles, keeping all wells except one covered with sterile aluminium foil. Half of the plants were harvested immediately, and the rest at 9 days post infection (dpi). Harvested plants were put in tared tubes with 1 mL of 10 mM MgCl_2_, weighed, cooled on ice, and ground in a tissue homogeniser (*Precellys* 24; https://www.bertin‐instruments.com) with 3 mm silica beads (2 × 30 s at 5000 rpm, with cooling on ice between runs). Dilutions of homogenates were plated on 0.2× PDA, and colonies were counted after 4 days. Additionally, seedling sterility was periodically checked by plating ground uninfected leaf samples on LB media and 1× PDA.

### 2.4 Histological Staining

Biofilm formation was quantified using crystal violet staining (Wilson et al. 2017). Four‐day‐old cells were harvested, washed, and suspended at OD_600_ = 0.02. Yeast suspension (200 μL) was added to 800 μL of 0.2× PDB (final OD_600_ = 0.004) and grown in polystyrene 48‐well plates (CELLSTAR Cell Culture Multiwell Plates, TC treated, Greiner Bio‐One) without shaking. To prevent contamination, each yeast species was separated by empty wells containing only media. After eight and 16 days, wells were rinsed to remove loosely adherent cells, stained for 15 min with 1% crystal violet solution, and photographed. Subsequently, biofilm‐bound dye was dissolved in 100% ethanol and quantified spectrophotometrically (λ600).

Crystal violet staining was used to visualise M11 cells (Valadon et al. 1962) and biofilms (Wilson et al. 2017) in and on infected leaves; briefly, 0.5% crystal violet staining solution was prepared in aqueous 20% methanol. Leaves of wild type *Arabidopsis* that had been infiltrated with *Taphrina* strain M11 at 3–7 dpi were cleared in 90% acetone, placed on a slide, and stained with a drop of the staining solution for 5–10 s at room temperature, de‐stained with ddH_2_O as required, and mounted in 60% glycerol. Samples were observed under a Leica compound microscope (MZ 2500; https://www.leica‐microsystems.com) with a magnification of 200× to 400×.

Trypan blue staining was used to visualise hypersensitive response‐like cell death in infected soil grown plants. Whole leaves were stained by boiling for ca. 1 min in a 1:1 dilution of trypan blue staining solution (0:05%) in lactophenol (1:1:1, glycerol: 85% lactic acid: phenol) in 95% ethanol. Samples were cleared at room temperature in chloral hydrate (2.5 g chloral hydrate per 1 mL ddH_2_0) with several changes of de‐staining solution until clear.

DAB (3,3’‐Diaminobenzidine; Sigma; www.sigmaaldrich.com) staining was used to visualise the *in planta* accumulation of H_2_O_2_ (Jambunathan 2010). DAB stain solution (0.1% w/v) was prepared fresh and protected from light. Four‐week‐old soil‐grown *Arabidopsis* grown under an 8/16 h photoperiod were hand inoculated and stained for 2 h in a closed container in the dark at high humidity. DAB solution was infiltrated into the leaves by vacuum infiltration. The staining reaction was stopped by immersing samples in clearing solution (3:1 solution of 95% ethanol in lactophenol).

β‐Glucuronidase (GUS) activity staining was used to investigate activation of host plant auxin and cytokinin transcriptional responses during infection using transgenic *Arabidopsis* with the following promoter‐reporter systems; the auxin‐responsive DR5 promoter or the cytokinin‐responsive TCS promoter fused to the β‐glucuronidase (GUS) reporter; denoted as DR5::GUS and TCS::GUS, respectively. Four‐week‐old soil‐grown *Arabidopsis* grown under an 8/16 h photoperiod were infected by hand infiltration. Positive controls for DR5::GUS were treated with 2, 5, and 10 μM indole acetic acid; for TCS::GUS controls were 2, 5, and 10 μM 6‐benzylaminopurine; all negative controls were treated with a mock infection of MgCl_2_ with Silwet. GUS staining solution contained 1 mM 5‐bromo‐4‐chloro‐3‐indolyl b‐D‐glucuronide dissolved in methanol, 5 mM potassium ferricyanide, and 5 mM potassium ferrocyanide in 50 mM sodium phosphate buffer and adjusted to pH 7.2. For histochemical staining, seedlings were fixed with ice‐cold 90% acetone for 1 h, washed two times with ice‐cold wash solution (50 mM sodium phosphate buffer, pH 7.2) for 30 min for each wash. Seedlings were vacuum infiltrated for 5 min and kept at room temperature in GUS staining solution. Stained seedlings were washed two times with absolute ethanol, then cleared and stored in 70% ethanol.

### 2.5 Promoter‐RUBY Lines

RUBY is a novel non‐invasive plant reporter system based on the conversion of tyrosine to the strongly red/purple coloured betalain (He et al. 2020). Promoter‐RUBY lines were constructed with several pathogen or hormone responsive promoters (Baral et al. 2024). For treatment of promoter‐RUBY lines, approx. three‐week‐old plants were hand injected with either water, 200 nM flg22, or autoclaved 0.9 g/L M11 cell walls suspension. Pictures were taken 72 h post injection.

### 2.6 Leaf Symptom Assays

To investigate leaf curling, leaves of soil‐grown plants, grown under an 8/16 h photoperiod, were infected with strain M11 and *T. betulina,* then at 14 dpi were transversely cross‐sectioned halfway between the leaf base and tip by hand using a razor blade and photographed. Curling index was measured from photos of leaf sections using Image J (https://imagej.nih.gov/ij/), as in Booker et al. (2004), and shown in Figure S1.

To quantify leaf bending, leaves were photographed at 14 dpi; then leaf bending was measured using ImageJ. Leaf bending was quantified by measuring the angle between the base of the petiole and the leaf tip, as illustrated in Figure S1.

### 2.7 *T. tormentillae* M11 Cell Wall Preparation and Immune Elicitation Tests

Cell wall preparations and root growth inhibition assays were performed as previously described in Wang et al. (2022). Briefly, seedlings were exposed to cell wall preparation for 8 days, and their root growth was compared between treatment and control plates. For mutant screening, a 10‐day treatment was used to enhance root phenotype. Mutant and wild type seedlings were grown on treatment plates in alternating groups of five to account for potential plate location effects. Root length was measured in ImageJ software. At 14 days, seedlings were also assessed for shoot phenotypes, shoot weight, and chlorophyll content. Shoot phenotypes were assessed in the primary screen visually and as the weight of five pooled seedlings in the secondary screen. Chlorophyll was extracted at +65°C in DMSO, and sample absorbance was measured at 652 nm to approximate the total chlorophyll content.

For stomatal immune response, four‐week‐old plants grown at 12 h/12 h light/dark were used. Plants were sprayed with either water or autoclaved 0.9 g/L cell wall suspension using an airbrush gun. To ensure uniform wetting of the leaf surface, Silwet‐L77 was added to a final concentration of 0.012%. Two leaves per plant were sampled 45 min after treatment. Stomatal impressions were prepared by applying Xantopren Mucosa M mixed with Xantopren Activator to the abaxial side of the leaf. To visualise stomata, impressions were painted with clear, quick‐drying nail polish and photographed at 200X magnification. Stomatal aperture was calculated by dividing stomatal width by length.

### 2.8 qPCR

For qPCR experiments, 22‐day‐old, soil‐grown plants cultivated under a 12/12 h photoperiod were used. Four to six fully expanded leaves were hand injected with a suspension of eight‐day‐old *Taphrina* cells in 10 mM MgCl_2_, OD_600_ = 0.3, or just 10 mM MgCl_2_ (mock). Plants were left in high moisture for 1 day. Rosettes were pooled by six, resulting in three samples per treatment, and flash frozen two dpi. Samples were ground in liquid nitrogen, and RNA was extracted with GeneJET Plant RNA Purification Mini Kit (Thermo Scientific). RNA concentration and quality were checked with NanoDrop. RNA (3 μg) was treated with DNAseI. Reverse transcription was performed with RevertAid Premium Reverse Transcriptase (Thermo Scientific) and cDNA was diluted to 100 μL. From this, 3 μL were used as a template in RT‐qPCR carried out on CFX384 Touch real‐time PCR detection system (Bio‐Rad) with 5 × HOT FIREPol EvaGreen qPCR Mix (Solis Biodyne). Immune signalling marker genes, primer sequences, and efficiencies are listed in Table S2B. Analysis of qPCR results and quality control was performed using qBase 3.4 (CellCarta), (Hellemans et al. 2007). Three reference genes, *PP2AA3*, *TIP41*, and *YLS8*, with stable expression levels (M‐value in experiments, 0.1–0.2) were used for normalisation. Data are scaled to control treatment average values.

### 2.9 Genome Sequencing, Assembly, and Analysis

DNA extraction, genome sequencing, and assembly were performed as previously described in Wang, Sipilä, et al. (2019). In short, chromosomal DNA was extracted (Hoffman 1997) and its quantity and quality assessed by Qubit fluorometer and Nanodrop ND‐1000 (Thermo Fisher Scientific, USA). Paired‐End DNA libraries were prepared according to the manufacturer's guide for sequencing with MiSeq System (Illumina, California USA) at the DNA Sequencing and Genomics Laboratory, Institute of Biotechnology, University of Helsinki. Raw data are available at the NCBI under the accession SRX4936057 (Sipilä and Overmyer 2018). Resulting reads were assembled with SPAdes version 3.1.1 pipeline (Bankevich et al. 2012) and quality determined using the QUAST tool version 5.0 (http://quast.sourceforge.net/). Genome completeness was assessed using gVolante integrated BUSCO v5 with the fungal ortholog dataset (Nishimura et al. 2019; Seppey et al. 2019).

Genome annotation was performed using Augustus version 2.5.5 (Stanke et al. 2008) which was trained on RNA sequencing results from the *Taphrina betulina* genome sequencing project (Bioproject: PRJNA188318). For further details on the automated annotation pipeline see Wang, Sipilä, et al. (2019).

To analyse the distribution of orthologous proteins in *Taphrina* M11 and selected members of the *Taphrinomycotina*, the OrthoVenn2 platform (Xu et al. 2019) was used with an E‐value cut‐off E < 0.01. Proteomes from the following whole genome sequencing projects were included: *T. deformans* JCM22205, BAVV01; *T. wiesneri* JCM22204, BAVU01; *T. populina* JCM22190, BAVX01; *T. flavorubra* JCM22207, BAVW01; *S. pombe* 972 h‐, ASM294v2. Proteomes of *P. arabidopsidicola* strain C29, QXMI01; *P. lactucaedebilis* YB‐4353, QXDS01; *P. gravidus* Y‐17093, QXDP01; *P. macrosporus* Y‐12879, QXDT01 were from (Wang, Sipilä, et al. 2019).

Conserved domains in annotated proteins of M11 were identified using HMMER software versions v3.2.1 and v3.3 by querying the Pfam database with E < 1e‐30 cut‐off (Finn et al. 2016). Conserved domains known to be characteristic of plant‐associated microbes were identified by comparison to the dataset in Levy et al. (2017).

For candidate effector‐like protein (CEP) comparison across different species, CEPs were defined as short, secreted proteins (SSPs) by identification of open reading frames (ORFs) in the size range 80 to 333 amino acids (aa) and screening with SignalP 4.1 tool (Petersen et al. 2011). SSP sequence secretion signals were trimmed and mature SSP peptides with ≥four cysteine residues were categorised as cysteine‐rich SSPs (CSSPs). In an alternate approach, ORFs were screened for secretion signals and transmembrane domains with SignalP 5.0 and Phobius, which utilise neural networks with training (Almagro Armenteros et al. 2019) and a hidden Markov model (Käll et al. 2004), respectively. CEPs were then predicted with EffectorP 3.0 (Sperschneider and Dodds 2022) from these secreted ORFs that lacked transmembrane domains.

To analyse predicted CEPs for the presence of conserved, genus‐specific motifs identified in Wang et al. (2020), FIMO version 5.5.3 was used (Grant et al. 2011). Query motifs were generated with MEME version 5.5.3 (Bailey and Elkan 1994). Conserved domain sequences were identified with the Web CD‐Search tool. Additionally, to predict the potential function of CEPs, a BLAST search against the PHI base (Pathogen–Host Interactions database; http://www.phi‐base.org/) 4.15 protein sequences was performed (*E*‐value < 0.01).

Orthologs for enzymes of interest were identified using the BLASTp tool to search the M11 and other *Taphrina* genomes with the query sequences provided in the supplemental files. The identity of proteins was further confirmed by performing a BLASTp search against the Swiss‐Prot database (default parameters). For the alignment of chitin synthases, the Clustal Omega multiple sequence alignment program was used (Madeira et al. 2019).

These sequence data are available at the Genebank (NCBI) database under the following accession numbers: BioProject, PRJNA487587; BioSample, SAMN09906266; Sequence Read Archive, SRX4936057.

## 3 Results

### 3.1 *Taphrina* Strains Isolated From Wild *Arabidopsis*

**FIGURE 1:**
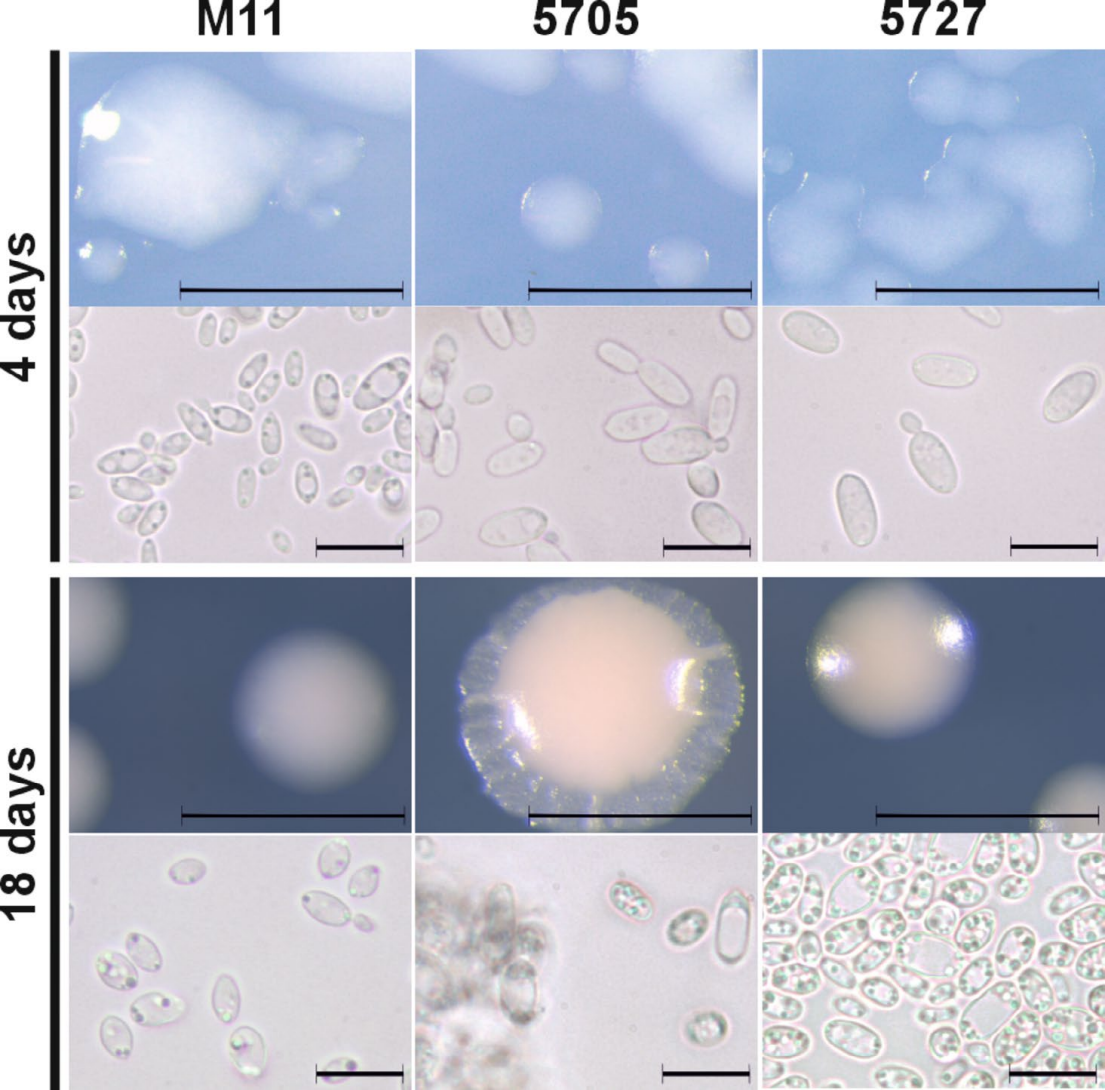
Cell and colony morphology differences in *T. tormentillae* strains. *T. tormentillae* cells were grown on 0.2 × PDA media and photographed after 4 and 18 days. Scale bar in colony pictures corresponds 1 mm, in cell pictures—10 μm. Used *T. tormentillae* strains from left to right: Strain M11 (*Arabidopsis* isolate), strain PYCC 5705/CBS 332.55 (birch isolate), PYCC 5727/CBS 339.55 (*Potentilla* isolate).

We have previously isolated yeasts from the phyllosphere of wild *Arabidopsis* (Wang et al. 2016) including OTU3, which had two *Taphrina* strains, M11 and M12, with ITS sequences (LT602860) that are identical to each other and most closely related (99% similarity) to *T. tormentillae*, a known pathogen of herbaceous host plants in the genus *Potentilla* (Wang et al. 2016; Fonseca and Rodrigues 2011). This suggests that M11 is a strain of *T. tormentillae*. The only two available *T. tormentillae* strains were used for comparison. Notably, all three strains displayed differences in colony and cell morphology (Figure 1 and Supporting Figure S2). At 4 days, strain M11 displayed smaller and more variable cell sizes, as visually assessed. At 18 days, M11 colony morphology was distinct with pale pink colour with less defined edges, while other strains were more orange in colour and PYCC 5705 colonies exhibited flattened edges. PYCC 5705 colonies were also resistant to disruption with inoculation loop and dispersal in water. Strains of *T. tormentillae* displayed little variation in carbon assimilation patterns (Table 1, Supporting Table S3, Data S1). Among these, only strain M11 was able to assimilate L‐xylose, D‐melezitose, and exhibited exceptionally robust growth on starch. PYCC 5727 uniquely utilised methyl‐ß‐D‐xylopyranoside and D‐fucose. The carbon utilisation profile above is consistent with published descriptions of *T. tormentillae* (Fonseca and Rodrigues 2011; Boekhout 2023). These results support that M11 is a strain *of T. tormentillae*.

**TABLE 1:** *Taphrina tormentillae* carbon source utilisation. Assimilation of different carbon sources was tested using API 50 CH strips from bioMérieux and yeast nitrogen base with amino acids and ammonium sulphate from US Biological. Growth was quantified after 3 weeks. Key to growth symbols: Very strong (+++), strong (++), medium (+), weak (w), unclear if present (?), absent (−). For full profile of carbon assimilation in *T. tormentillae* strains see Table S3.

	M11			PYCC5705			PYCC5727		
	R1	R2	R3	R1	R2	R3	R1	R2	R3
L‐Xylose	?	w	+	−	−	−	−	−	−
Me‐β‐Xyl^a^	−	−	−	−	−	−	w	+	++
Arbutin	+	−	w	−	−	−	−	−	+
D‐Maltose	+	++	++	?	+	−	−	w	−
D‐Melezitose	−	w	w	−	−	−	−	−	−
Starch	+++	+++	+++	++	++	++	w	+	+
Glycogen	w	w	w	−	−	−	−	?	w
D‐Lyxose	w	+	+	?	−	−	−	?	w
D‐Fucose	−	−	−	−	−	−	w	+	?
L‐Arabitol	+	+	+	?	?	?	?	w	−

^a^ Me‐β‐Xyl = methyl‐ß‐D‐xylopyranoside. R1‐R3, Replication 1–3.

To explore the environmental distribution of *T. tormentillae*, ITS sequences were queried against *Arabidopsis* microbiome studies and other environmental sequencing data (Table 2, Supporting Table S4). *T. tormentillae* was identified in two *Arabidopsis* microbiome studies (Table S4A) and in one, it was described to act as a heritable microbiome hub (Almario et al. 2022; Brachi et al. 2022). *T. tormentillae* was also present in diverse environments (Table 2, Supporting Table S4B, Data S2). Among these were a tendency for regions with cooler climates, soil samples, and importantly, multiple plant species not related to its known host.

**TABLE 2:** *Taphrina tormentillae* environmental occurrence. NCBI database was queried using BLAST with *T. tormentillae* ITS region sequences in June 2023. Hits with > 97% sequence similarity and > 95% sequence coverage were grouped according to source type and their appearance in independent sequencing projects counted. For the full BLAST results see Table S4.

Independent occurrences	Source
4	Soil in cold temperate region
3	Soil in high‐elevation region
3	Soil from arctic/subarctic region
2	Roots of a plant other than known host
2	Pine needles
1	Biofinish containing surface of pine sapwood impregnated with raw linseed oil
1	*Potentilla erecta* , Slovakia
1	*Tragopogon pratensis* , Germany
1	*Fagus sylvatica* phyllosphere, southern France
1	*Empetrum nigrum* fruit endophyte, Oulu, Finland
1	*Cladonia stellaris*, Russia
1	*Myotis evotis* bat wing surface, western North America
1	*Scolytus multistriatus* beetle, Sweden
1	Arctic stream, Svalbard, Norway
1	Rainwater, China
1	Fungal spore trap (air), Lithuania
1	Indoor surface swab, USA

### 3.2 M11 Interactions With *Arabidopsis*

**FIGURE 2:**
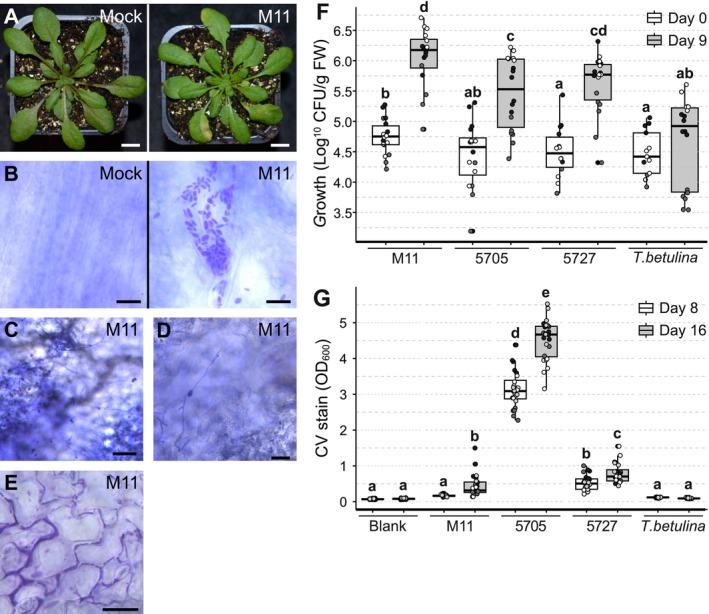
Host symptoms, *Taphrina* growth and cell morphology in infected *Arabidopsis*. (A) Four‐week‐old control and infected *Arabidopsis* were photographed at seven days post infection (dpi). *Taphrina* strain M11 infiltration (M11), mock infiltration using MgCl_2_ (mock). Scale bars = 1 cm. (B–E) Visualisation of the morphology of *Taphrina* strain M11 cells in the leaf of *Arabidopsis* after hand infiltration using a needless syringe with four‐week‐old *Arabidopsis*. (B), Control mock infiltrated leaf (Scale bar = 10 μm), and M11 infiltrated leaf with yeast cells (Scale bar = 10 μm) one dpi. (C), Hyphal cells were observed at three dpi (Scale bar = 5 μm). (D), Close up of hyphal cells (Scale bar = 2 μm). (E), Biofilm formation was observed on the leaf surface at three dpi, as shown, or later (Scale bar = 30 μm). (F) *Taphrina* growth in the phyllosphere of in vitro *Arabidopsis*. *Taphrina* yeast suspensions were sprayed onto the surface of 24‐day‐old, sterile wild type *Arabidopsis*. Used *T. tormentillae* strains from left to right: Strain M11 (*Arabidopsis* isolate), strain PYCC 5705/CBS 332.55 (birch isolate), PYCC 5727/CBS 339.55 (*Potentilla* isolate). The birch pathogen *T. betulina* used here as a non‐host response control. Yeast growth was quantified through cultivation‐based technique immediately after spraying and at nine dpi. Combined data are presented from three independent experiments (*n* = 6) for each experiment, data points from each experiment are represented with a different colour. Letters above boxplots represent significance groups from a Tukey's test performed on linear mixed model computed in R with biological repeats as a random effect. Means *α* ≥ 0.05 share a common letter. (G) In vitro biofilm formation by *Taphrina* species using the same strains as described above. The presence of adherent, biofilm‐forming cells were monitored by spectrophotometric quantification of released crystal violet stain after treatment with a with 1% solution and ethanol extraction. Pooled data from four independent experiment replicates is presented (*n* = 6 for each, total *n* = 24), data points from each experiment are plotted with a different colour. Letters above boxplots represent significance groups as described above. CFU, colony forming units; CV, crystal violet; FW, fresh weight.

Preliminary tests suggested M11 caused disease on *Arabidopsis* (Wang 2015) prompting further investigation. Infecting *Arabidopsis* leaves with strain M11 resulted in leaf deformations and chlorosis (Figure 2A). Based on this result, *Taphrina* strain M11 was targeted for genome sequencing and characterisation of its interaction with *Arabidopsis*.

The morphology of M11 cells *in planta* was monitored using microscopic observation of crystal‐violet‐stained infected tissues. Infected leaf tissues exhibited clusters of M11 yeast cells at one dpi (Figure 2B). In some tissues at slightly later time points (3 dpi) the growth of infectious hyphae was detected (Figure 2C,D). In some infected leaves, dark staining cell aggregates with the appearance of a biofilm were observed (Figure 2E).

Growth of M11, two closely related *T. tormentillae* strains, and the non‐host control, *T. betulina*, on axenic 24‐day‐old *Arabidopsis* was measured (Figure 2F). All three strains of *T. tormentillae* exhibited the ability to multiply on *Arabidopsis* to slightly different degrees, while *T. betulina* showed little to no growth.

Biofilm formation was quantitatively assayed in vitro on polystyrene plates (Figure 2G). The non‐host control, *T. betulina*, was not able to form biofilms in this assay. In contrast, all strains of *T. tormentillae* formed biofilms, albeit to a varying extent. Strain PYCC 5705 demonstrated a strong ability to form biofilms (Figure 2G). Strain PYCC 5727 exhibited an intermediate level of biofilm formation, and M11 formed small, adherent biofilm‐like patches only after 16 days.

### 3.3 M11 Causes Leaf Deformation Symptoms

**FIGURE 3:**
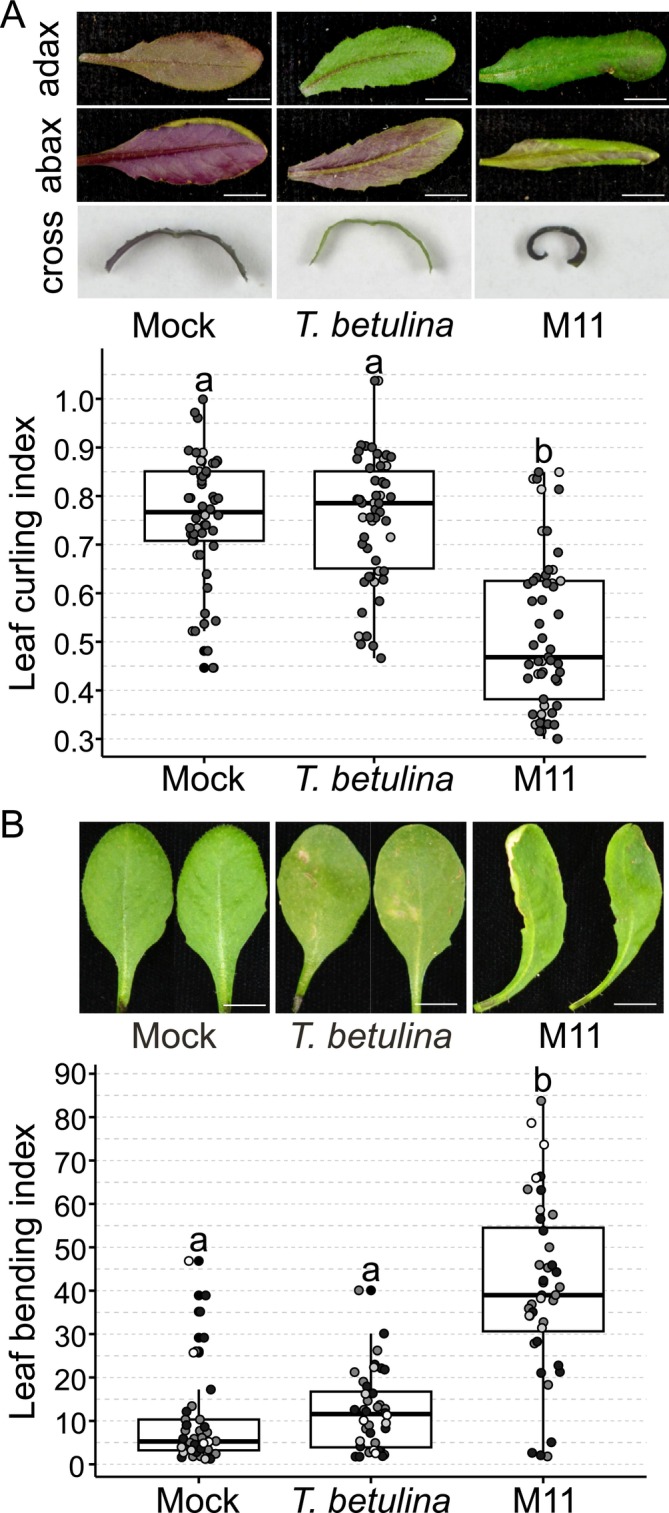
Leaf deformation phenotypes. Subtle leaf deformation phenotypes observed in response to infection with *Taphrina* species. Leaves of four‐week‐old soil grown *Arabidopsis* were hand infiltrated with a needless syringe delivering cell suspensions of M11, *T. betulina*, or mock treatment with 10 mM MgCl_2_. Observations were made at 14 days post infection (A) Leaf curling was seen in primary infected leaves and in new leaves that developed after infection. Leaves were photographed on the adaxial side (adax.), abaxial side (abax.) and hand sectioned at their mid‐point. The ca. 1 mm hand section was placed on its side and photographed to reveal its curvature (cross.). Scale bars = 0.5 cm. Leaf curling was quantitatively measured from photographs with ImageJ and the leaf curling index calculated as described (Figure S1A; (Booker et al. 2004), which results in lower scores for leaves with greater curvature. Box plots depict pooled results from three independent biological replicates (total *n* = 54), data points from each experiment are represented with a different colour. Letters above the box plots indicate significance groups calculated with ANOVA and Tukey HSD post hoc test. Means *α* ≥ 0.05 share a common letter. (B) Leaf bending phenotypes were documented in photographs of primary infected leaves and quantitatively measured from photographs with ImageJ. The leaf bending index was calculated as the angle between a line defined by the petiole and a second line defined by the leaf mid‐point and tip, as shown (Figure S1B). Box plots depict pooled results from four independent biological replicates (*n* = 5 per biological repeat, total *n* = 40), data points from each experiment are represented with a different colour. Letters above the box plots indicate significance groups calculated with Kruskal–Wallis test and Dunn's post hoc test (*α* = 0.05).

At later time points, infected leaves exhibited subtle symptoms reminiscent of leaf deformations caused by other *Taphrina* species. These late‐presenting symptoms were quantified as leaf curling and leaf bending at 14 dpi (Figure 3). Leaf curling was measured using a curvature index (Figure S1A), where smaller values indicate increased curvature. M11‐infected plants exhibited significantly enhanced leaf curling compared to controls (Figure 3A). Infections also caused leaf bending (Figure 3B), which was quantified using a leaf bending index (Figure S1B). A significantly increased leaf bending index was observed in response to infection with M11 compared to controls (Figure 3B). Both of these leaf phenotypes were specific to M11 infection, as the non‐host control was not significantly different from the mock control (Figure 3A,B).

### 3.4 *Arabidopsis* Immune Response to M11 Infection

**FIGURE 4:**
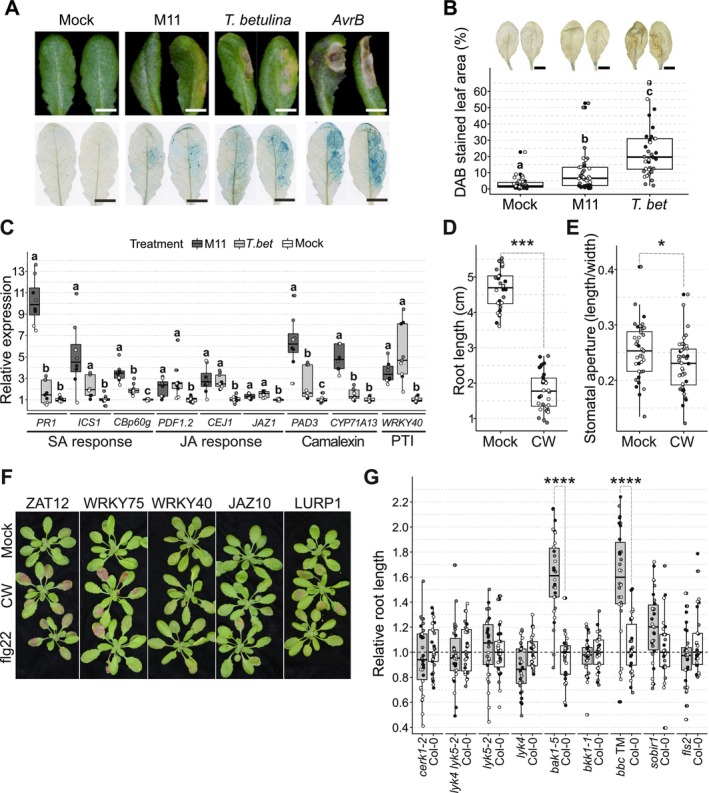
*Arabidopsis* immune response to *Taphrina*. Plants were subjected to treatments with live yeast cells (A–C) or isolated cell walls of *Taphrina* M11 (D–G), and various immune responses observed. (A–C) Immune response two days post infection (dpi) after live‐cell infiltration into *Arabidopsis* leaves with a needless syringe. (A) Cell death in infected four‐week‐old plants. Infected leaves were photographed (top) and trypan blue histological staining was used to visualise cell death (bottom) at two dpi. Leaves were infiltrated with (from left to right): Mock solution (10 mM MgCl_2_), *Taphrina* strain M11 (M11) isolated from wild *Arabidopsis*, birch pathogen *T. betulina* used here as a control for a non‐host response, and avirulent *Pseudomonas syringae* pv. tomato strain DC3000 transgenically expressing *AvrB* (*AvrB*) used here as a positive control for the hypersensitive response. (B) ROS accumulation during infection of four‐week‐old *Arabidopsis* with *Taphrina* strain M11 (M11) or *T. betulina* (*T.bet*) was monitored at two dpi by histologically visualising H_2_O_2_ accumulation with 3,3′‐diaminobenzidine (DAB) staining. Leaf halves of four‐week‐old soil grown *Arabidopsis* were hand infiltrated with a needless syringe. Stained and cleared leaves were photographed (top) and stain was quantified digitally by counting brown pixels in ImageJ (bottom). Pooled data from three independent experiment replicates (*n* = 12 for each, total *n* = 36), data points from each experiment are represented with a different colour. Letters above the box plots indicate significance groups calculated with Kruskal–Wallis test and Dunn's post hoc test (*α* = 0.05). (C) Immunity marker gene expression change relative to negative control measured with qPCR after infection with *Taphrina* yeasts OD_600_ = 0.3 in 10 mM MgCl_2_. Water (negative control), *T. betulina* (non‐host control), and *T. tormentillae* M11. Plants (22‐day‐old) were hand infiltrated through stomata, six plants pooled per sample and flash‐frozen two dpi. Combined data are presented from three independent experiments, *n* = 3 for each experiment (total *n* = 9). Data points from each experiment are represented with different colour. Letters above boxplots represent significance groups determined with non‐parametric Kruskal‐Wallis test followed by Dunn's posthoc test with Holm adjustment for multiple comparisons (*α* = 0.05). (D–G) *Arabidopsis* immune response to crude 0.9 mg/mL *T. tormentillae* M11 cell wall extract (M11 CW). (D) Root length was measured to assess growth‐defence trade‐off in seedlings exposed to M11 cell wall extract. Four‐day‐old seedlings were transferred to either 0.5 × MS agar control plates or plates supplemented with cell wall extract and photographed for root length measurements after eight days. Data points from each independent experiment replicate are represented with different colour. One‐way ANOVA was used to test for treatment effect. Student's *t*‐test was used to compare means of mock‐treated and cell‐wall‐treated root lengths (*p*‐value < 2.2e‐16). (E) Stomatal immune response was quantified by measuring stomatal aperture (width/length) 45 min after spraying four‐week‐old, soil grown plants with 0.012% Silwet‐L77 control solution or 0.012% Silwet‐L77 with M11 cell wall extract. Two leaves per plant were sampled. Stomata were visualised using stomatal imprint method and measured in ImageJ. Experiment was replicated four times. Data points from each experiment are plotted in different colours and represent average stomatal aperture per leaf (total *n* = 40). Two‐way ANOVA was used to test for treatment and plant effect. Both had statistically significant effect on stomatal aperture (*α* = 0.05). Student's *t*‐test revealed small, but statistically significant (*p* = 0.025) difference between stomatal aperture in mock‐treated (mean 0.253) and cell‐wall‐treated (mean 0.228) leaves. (F) Expression of reactive oxygen species (ROS), pattern triggered immunity (PTI), salicylic acid (SA), and jasmonic acid (JA) marker genes was visually assessed in 21–24‐day‐old promoter‐*RUBY* lines treated with M11 cell wall extract (M11 CW), 200 nM flg22 (positive control) or water (negative control). Activation of the promoter results in appearance of red colour visible with naked eye. *ZAT12, WRKY75*—ROS marker genes, *WRKY40*—PTI marker gene, *JAZ10*—jasmonic acid marker gene, *LURP1*—pathogen marker gene. Experiment was replicated three times, representative plants are shown (for pictures of all plants see Figure S3). (G) Involvement of known pattern recognition receptors and co‐receptors in recognition of *Taphrina* M11 cell walls. Root growth‐defence trade‐off assays were performed as described previously. Root length was measured ten days after transplanting. Root length is normalised against growth on 0.5 × MS agar control plates and plotted as relative growth to wild type. Each mutant is grouped together with the respective wild type that has been grown on the same treatment plate. Mutant abbreviation: *bbc* TM, *bak1‐5 bkk1‐1 cerk1‐2* triple mutant. Pooled data from three independent experiment replicates are displayed, data points from each experiment are plotted in a different colour. Repeated Mann–Whitney *U* tests with Holm correction for multiple testing were performed to compare mutant and respective wild type growth. “****” indicates *p* < 0.0001.

The activation of cell death upon *Taphrina* infection was monitored visually (top) and with trypan blue staining (bottom) at two dpi (Figure 4A). M11 treatment resulted in chlorosis and leaf deformation symptoms that were associated with a very low level of cell death. Activation of hypersensitive response (HR)‐like cell death was observed in leaves challenged with the non‐adapted *T. betulina*; however, this was less than in leaves treated with the avirulent *Pst* DC3000 *AvrB* strain, used here as a control for a strong HR.

DAB staining was used to visualise the accumulation of H_2_O_2_, and brown coloured pixels were quantified (Figure 4B). Compared to the mock treatment, there was a small but significant (*p* = 2.106E‐10) increase in DAB staining in M11 leaves, which were stained significantly less than leaves treated with the non‐host control, *T. betulina*.

To probe *Arabidopsis* responses at the molecular level, immune marker gene expression was assessed at two dpi (Figure 4C). Both M11 and *T. betulina* weakly induced jasmonic acid response markers (*PDF1.2, CEJ1, JAZ1*) and increased transcript levels for the PTI marker *WRKY40*. Differences were observed in the salicylic acid (SA) response and camalexin biosynthesis marker genes. M11 caused a stronger upregulation in the salicylic acid response (*PR1, ICS1, CBp60g*) and camalexin biosynthesis (*PAD3, CYP71A13*) marker genes, whereas *T. betulina* had a weak (*ICS1, CBp60g, PAD3*) or no effect (*PR1, CYP71A13*).

### 3.5 Cell Walls of *T. tormentillae* Are Recognised by *Arabidopsis*

The ability of M11 yeast‐cell walls to elicit an immune response was investigated using assays based on the inhibition of root growth and closure of stomata by immune activation (Figure 4D,E). Root growth was inhibited in seedlings grown on plates containing crude M11 cell wall preparations (Figure 4D). Cell wall treatments caused a reduction in stomatal aperture in leaves of adult soil‐grown plants (Figure 4E).

To further dissect *Arabidopsis* immune response to M11 cell walls, the non‐invasive RUBY reporter was used to visualise promoter activity of immune signalling genes. Five promoters transcriptionally responsive to various pathogen and PAMP treatments, hormone treatments, and reactive oxygen species were selected (Table S11) (Baral et al. 2024). Infiltration with flg22 was also used as a PAMP control. Infiltration with M11 cell walls (Figure 4F and Figure S3) resulted in strong RUBY accumulation at 72 h with promoters primarily responsive to ROS (*ZAT12* and *WRKY75*). *LURP1* and *WRKY40*, which are responsive to flagellin and a biotrophic Oomycete pathogen, exhibited medium and low accumulation, respectively. No RUBY accumulation was observed in the JA and wounding responsive *JAZ10*. Flg22 strongly activated *ZAT12* and *WRKY75*, but no RUBY accumulation was observed with *LURP1* (Figure 4F, Figure S3). These findings further support that the M11 cell wall contains PAMP(s) and is able to activate *Arabidopsis* immune signalling.

Reverse genetics was used to test a panel of receptor knock‐out mutants (Table S1) as candidate immune receptors involved in the recognition of M11 cell walls. This included known PAMP receptors and co‐receptors, and candidate receptors of *Taphrina* cell wall components. For the latter, a collection of L‐type lectin receptor‐like kinases (LecRKs) and proteins (LecPs) was used. Additionally, selected malectin‐type RLKs and RLPs, secreted jacalin‐type RLPs, and G‐type LecRKs were targeted. These included genes known to respond to biotic stress (Bellande et al. 2017). When possible, candidates with yeast‐responsive expression profiles were used (Data S3).

The observed root growth inhibition upon immune activation with M11 cell walls (Figure 4D) was used for a genetic screen based on the root growth assay. In total, 108 mutant lines were included in the primary screen, which was replicated twice. The *bak1‐5* and LECRK−IV.1_m1 mutants exhibited reduced sensitivity to M11 cell walls (Figure S4). Additionally, larger shoot size and less chlorosis were noted in two independent knock‐out mutants of the *SD1‐13* gene (AT1G11350) (Figure S5). Genotypes were confirmed by PCR for known receptors/co‐receptors, and candidates with altered phenotypes in the primary screen. As seen in a previous study, no T‐DNA insertion was found in the LECRK‐IV.1_m1 (SALK_019496C) mutant (Wang et al. 2014). In the secondary screen, LECRK−IV.1_m1 and the *SD1‐13* mutants did not show phenotypes. However, reduced sensitivity to M11 cell walls was confirmed for *bak1‐5* and a triple co‐receptor mutant with the *bak1‐5* mutation (*bak1‐5 bkk1‐1 cerk1‐2*), but no other known receptors or co‐receptors exhibited phenotypes (Figure 4G). Phenotypes were observed in both roots (Figure 4G) and shoots (Figure S6).

### 3.6 M11 Activates Host Growth‐Hormone Responses

**FIGURE 5:**
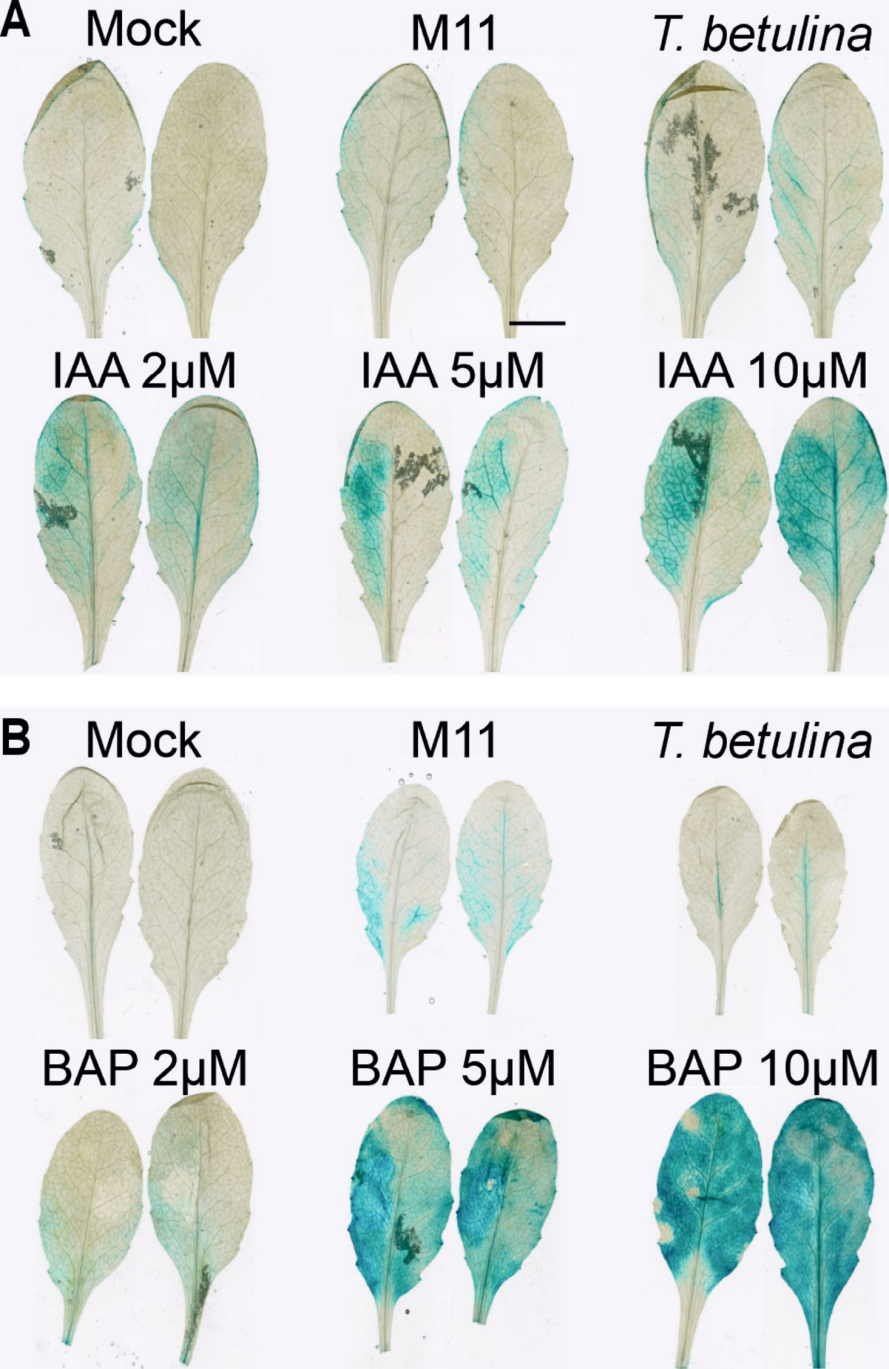
Activation of host hormone responses during *Taphrina* infections. Activation of the *Arabidopsis* auxin and cytokinin transcriptional responses in response to infection with *Taphrina* strain M11 or *T. betulina*. Infections of four‐week‐old soil grown *Arabidopsis was* by hand infiltration of *Taphrina* cell suspensions using a needless syringe. Left hand leave halves were infiltrated and histologically stained to visualise β‐glucuronidase (GUS) activity at 2 days post infection. (A) Activation of the plant auxin response to various treatments, as indicated, using plants transgenically bearing the auxin responsive pDR5::GUS promoter‐reporter construct. As a positive control, treatments with the auxin, indole acetic acid (IAA), were used at the concentrations, 2, 5, and 10 uM and the negative control was mock infected with 10 mM MgCl_2_. (B) Activation of the plant cytokinin response to various treatments, as indicated, using *Arabidopsis* transgenically bearing the cytokinin responsive pTCS::GUS promoter‐reporter construct. As a positive control, treatments with the cytokinin, benzylaminopurine (BAP), were used at the concentrations, 2, 5, and 10 μM and the negative control was mock infected with 10 mM MgCl_2_.

*Taphrina* are known producers of the plant hormones auxin and cytokinin, which have a variety of roles in plant‐microbe interactions (Spaepen and Vanderleyden 2011). To address this, the activation of host hormone transcriptional responses was monitored during infection using two *Arabidopsis* lines transgenically bearing either cytokinin‐responsive or auxin‐responsive promoter‐GUS reporter constructs. These reporter lines were infiltrated with *Taphrina* strain M11 or control hormones, followed by histological staining of GUS activity to visualise hormone response activation (Figure 5A,B). Auxin (indole acetic acid) and cytokinin (6‐benzylaminopurine) were used at three different concentrations as positive controls. The results demonstrate that both M11 and *T. betulina* were able to activate *Arabidopsis* auxin (Figure 5A) and cytokinin (Figure 5B) transcriptional responses. In response to both M11 and *T. betulina*, the auxin response was similar in extent and spatial distribution, localised to the leaf periphery and secondary vasculature (Figure 5A). However, the cytokinin transcriptional response to M11 infection was both stronger and involved more tissues, especially around the base of the leaf, while infection with *T. betulina* resulted in only a small response along the primary leaf vein (Figure 5B).

### 3.7 *Taphrina* M11 Genome

**FIGURE 6:**
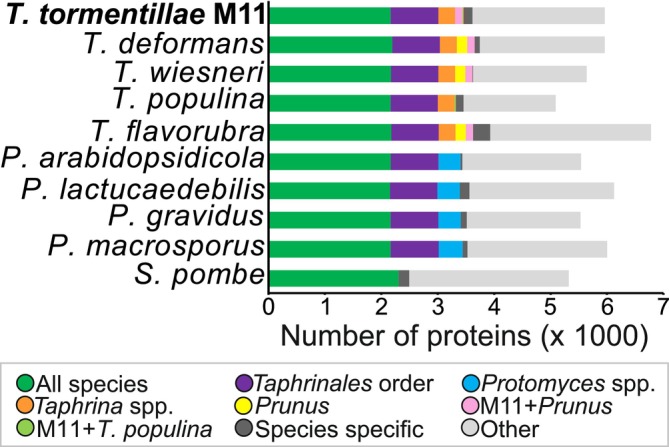
Ortholog distribution in M11 and other Taphrinomycotina yeasts. Ortholog analysis was performed using OrthoVenn2, with *E* < 0.01 cut‐off for ortholog calling. Fission yeast (*Schizosaccharomyces pombe*) was used as an outgroup.

In order to discover molecular components involved in *Taphrina*‐plant interactions, the genome of *Taphrina* strain M11 was sequenced, resulting in a high‐quality draft genome assembly of 13.6 Mbp in 234 contigs, which was 96.97% complete, as assessed by BUSCO analysis (Table S5). *Taphrina* M11 genome characteristics were consistent with other sequenced *Taphrina* species, including low repetitive sequence content (Table S6). Similar to other *Taphrina* species, 5808 proteins were annotated (Table S7). Ortholog distribution was monitored across M11 and several other species of *Taphrina* (Figure 6). Additionally, several species of the closely related genus *Protomyces* were used for comparison, and *S. pombe* was used as an outgroup to define more broadly conserved genes. On average 38.5% of all proteins were common across the subphylum Taphrinomycotina; however, these were not specific to the Taphrinomycotina as they included conserved eukaryotic housekeeping genes. The genera *Taphrina* and *Protomyces* shared 14.9% of their genes, while 5.3% were unique to the genus *Taphrina* (Figure 6). The genus *Protomyces* had slightly more genus‐specific proteins*—*7.1%. Only 151 proteins (2.6%) were found to be unique to *Taphrina* strain M11. *Taphrina* M11 shared a sizable portion (132 proteins, 2.3%) of orthologous proteins with *Taphrina* species pathogenic on *Prunus* species, but not with *T. populina*, which is pathogenic on *Populus* (20 proteins, 0.3%). Additionally, conserved domains characteristic of plant‐associated microorganisms were identified in *Taphrina* M11 annotated proteins (Data S4) (Levy et al. 2017), consistent with the association of M11 with plants.

### 3.8 Candidate Effector‐Like Proteins

Candidate effector‐like proteins (CEPs) were identified in the genomes of M11 and other ascomycete yeasts with different lifestyles (Table S6). In M11, a total of 18,660 short (80–333 aa) ORFs were identified, 767 of which contained a secretion signal and were defined as short secreted proteins (SSPs; Table S6). Of the SSPs, 337 contained four or more cysteine residues and were defined as cysteine‐rich SSPs (CSSPs). Plant pathogenic and rhizosphere‐associated fungi had, on average, more SSPs and CSSPs than saprophytic fungi (Table S6). The number of SSPs and CSSPs (767 and 337 respectively) in M11 was similar to other *Taphrina* species and consistent with its plant pathogenic lifestyle.

CEP analysis was validated with EffectorP 3.0, as an alternate method. This approach yielded 683 CEPs, among which 397 were cysteine‐rich (C ≥ 4) (Data S5). In total, 165 of the CEPs were classified as apoplastic effectors and 485 as cytoplasmic. Ten percent of the CEPs contained at least one of seven conserved motifs previously described to be present in *Taphrina* effectors (Wang et al. 2020). Motif 7 was most common, present in 19 CEPs. Only 10 CEPs displayed a modular structure with more than one motif (Data S5). PHI‐base search for CEP homologues resulted in 74 CEPs with hits, 49 of which were for homologues of proteins with an effect on virulence or pathogenicity in other species (Data S6).

### 3.9 Putative Plant Hormone Biosynthesis Pathways

Genes involved in auxin and cytokinin production (Tables [Link], [Link]) were identified in the M11 genome using known biosynthesis genes from other species, which represented four possible routes for auxin biosynthesis in microbes; specifically, the indole‐3‐acetamide, indole‐3‐pyruvate, indole‐3‐acetonitrile, and tryptamine pathways. The *Taphrina* M11 genome contained complete enzymatic machinery for auxin production via three different routes, the indole‐3‐acetamide, indole‐3‐pyruvate, and tryptamine pathways (Tables [Link], [Link]).

Two key enzymes of cytokinin biosynthesis were also identified in the M11 genome (Table S8), tRNA‐isopentenyltransferase and cytokinin phosphoribohydrolase. However, the presence of other enzymes involved in this pathway cannot be excluded, as no query sequences for other known steps of the pathway were available from closely related fungi.

### 3.10 Biosynthesis of Potentially Immunoactive Cell‐Wall Polysaccharides

Putative cell wall biosynthesis proteins were identified in the *Taphrina* M11 genome (Table S10, Data S7 and S8). Two conserved chitin synthases (class I and III) in the *Taphrina* M11 genome and three conserved chitin synthases in *T. deformans* (class I, II, III) were identified (Figures [Link], [Link]). Additionally, a putative chitin deacetylase homologue was found, which may be used by M11 to convert chitin into chitosan, two chitinases that could be involved in chitin remodelling, and three chitin transglycosylases that could crosslink chitin to β‐glucan (Table S10, Data S7).

The M11 genome contains homologues of proteins necessary for the production of β‐glucans both with β‐1,3‐linkages and β‐1,6‐linkages (Table S10, Data S7). The presence of α‐glucan was also queried; two α‐1,3‐glucan/α‐1,4‐glucan synthase genes were identified in the M11 genome (Table S10, Data S7). Also, a set of putative N‐glycosylation and O‐glycosilation enzymes was identified, including potential galactosyltransferases and four copies of the Och1 gene, which encodes the enzyme that adds the first fungal specific mannose residue during N‐glycosylation.

## 4 Discussion

### 4.1 M11 Causes Disease on *Arabidopsis*

*Taphrina* strain M11 was found to have an ITS sequence 99% similar to that of *T. tormentillae* (Wang et al. 2016), suggesting that M11 is a strain of *T. tormentillae* (Vu et al. 2016; Boekhout et al. 2021). However, as previously noted, ITS sequences do not always offer good resolution to the species level in the genera *Taphrina* and *Protomyces* (Rodrigues and Fonseca 2003; Wang et al. 2021). Sequencing of protein‐coding genes, genome analyses, or additional sampling of reference material will be required to determine the relationship between M11 and *T. tormentillae*. Remarkably, the two other *Taphrina* strains tested, *T. tormentillae* strain PYCC 5727 and the closely related strain PYCC 5705, whose ITS sequence defined it as *T. tormentillae* (Rodrigues and Fonseca 2003), also multiplied on *Arabidopsis*, but to slightly lower levels (Figure 2F). This indicates that the ability to grow on *Arabidopsis* might be a common feature across *T. tormentillae*. Reciprocal infections of these strains on *Potentilla* species known to be hosts for *T. tormentillae* will be required in future studies to address the host range of these species.

Infections with *T. betulina*, which is not adapted and used here as a control for the non‐host defence response, resulted in accumulation of H_2_O_2_, activation of rapid HR‐like cell death, no significant growth *in planta*, and no late leaf‐deformation symptoms (Figures 2, 3, 4). These results indicate *Arabidopsis* has immunity against *T. betulina* and likely other *Taphrina* species. This may be based on PTI or other mechanisms. The HR‐like cell death observed here is considered a hallmark of ETI; however, ETI‐like resistance, based on direct or indirect effector recognition, is also a recognised mechanism of non‐host resistance (Panstruga and Moscou 2020; Oh and Choi 2022). Infections with *Taphrina* strain M11, which was isolated from wild *Arabidopsis*, resulted in an attenuated ROS response compared to *T. betulina*, a small amount of cell death that was consistent with symptom development, successful multiplication on *Arabidopsis*, and leaf symptom development including chlorosis and late presentation of leaf deformations. These findings support the idea that strain M11 is adapted to and pathogenic on *Arabidopsis*. M11 genomic features also support that it is a plant pathogen. These features include candidate effector proteins, with conserved motifs found in other *Taphrina* species. Conserved protein domains previously shown to be specific to plant‐associated microbes or biotrophic plant pathogens (Levy et al. 2017; Pandaranayaka et al. 2019) were also found.

Currently, no receptor‐mediated resistance against *Taphrina* is known at the mechanistic level; although several studies have begun to address this possibility. Evidence of genetic resistance against witches' broom disease caused by *T. betulina* segregating in natural populations of birch has been documented (Christita and Overmyer 2021). *Taphrina* resistance has also been addressed in peach (Goldy et al. 2017; Svetaz et al. 2017), where *Taphrina* causes significant economic losses and chemical fungicides are the only available means of control. Results below discuss some of the cell wall PAMPs encoded in the M11 genome and implicate a co‐receptor known to be involved in both ETI and PTI in *Arabidopsis*.

### 4.2 Defence Signalling in the M11‐*Arabidopsis* Interaction

Marker gene responses during infections exhibited some differences between the non‐host and pathogenesis response. The greater response seen with SA and camalexin marker genes upon M11 treatment at 48 h, as compared to that of *T. betulina*, is likely due to a rapid non‐host resistance mechanism that limits *T. betulina* growth. Alternatively, these pathways may not be involved in the *Arabidopsis* non‐host response to *T. betulina*. Previously, a higher SA response was seen in a peach cultivar with increased resistance (Svetaz et al. 2017). However, Svetaz et al. compared the response of two different peach genotypes to virulent *T. deformans*, but did not test non‐host resistance.

Microbial cell walls are known to be a common source of PAMPs due to their highly conserved nature. Taken together, the elicitation of multiple defence responses by M11 cell walls, including stomatal closure, inhibition of root growth, and defence gene promoter activity (Figure 4), indicates the presence of PAMPs in M11. Since many components of cell walls are highly conserved, similar PAMPs likely contribute to the immune response observed during both M11 and *T. betulina* infection.

Sequencing of the *Taphrina* M11 genome resulted in a high quality (96.97% complete; Table S5) assembly. Although completeness estimates were high, and repetitive sequence content low, the number of contigs was relatively high, as has been seen in other sequenced *Taphrina* genomes (Cissé et al. 2013; Tsai et al. 2014; Wang et al. 2020). Previously, molecular examination of chromosome band number by pulsed field gel electrophoresis indicated that all five examined *Taphrina* species had at least 17 chromosomes (Wang et al. 2020). This high number of chromosomes may contribute to assembly problems; however, *Taphrina* species may also have repetitive sequence islands, or other structures, that impede assembly of short‐read genome sequence data.

To identify potentially immunoactive *Taphrina* cell wall components, this genome was queried for known fungal PAMP biosynthesis genes and other known immunoactive carbohydrates (Table S10, Data S7). Conserved genes for chitin synthesis, remodelling, and crosslinking were found in the M11 and *T. deformans* genomes (Table S10; Figures [Link], [Link]); however, previous biochemical studies of cell walls did not find evidence of chitin in the yeast‐like cells of *Taphrina* (Valadon et al. 1962; Petit and Schneider 1983). In dimorphic fungi, cell wall composition changes depending on the growth form (Díaz‐Jiménez et al. 2012). Chitin was detected in *T. wiesneri* infected cherry leaf sections by staining with fluorescein‐isothiocyanate‐conjugated wheat germ agglutinin, which only bound to foot cells of asci (Komatsu et al. 2010). Loss or allocation of chitin to only reproductive structures are also seen in other Taphrinomycotina members. In *S. pombe*, the best studied yeast from the subphylum, chitin can only be detected in ascospores (Arellano et al. 2000). Members of the genus *Pneumocystis* are unculturable yeasts that are obligate pathogens of mammals, and remarkably the only fungi known that do not contain chitin (Ma et al. 2016). Conserved chitin synthases in *Taphrina* genomes suggest *Taphrina* could have detectable chitin only in some specific structures other than vegetative yeast cells. The M11 genome encodes a putative chitin deacetylase, which could transform chitin into the less immunogenic chitosan. Two α‐1,3‐glucan/α‐1,4‐glucan synthases are also encoded by M11 (Table S10). These could contribute to the masking of other cell wall PAMPs, as has been observed for the plant pathogen *Magnaporthe oryzae* and a range of human pathogenic fungi (Fujikawa et al. 2012; Ruiz‐Herrera and Ortiz‐Castellanos 2019).

Importantly, the *Arabidopsis* chitin receptors CERK1, LYK4, and LYK5 were not required for an immune response to *Taphrina* M11 cell walls. Chitin receptor mutants and wild type plants displayed similar levels of growth–defence trade‐off when treated with *Taphrina* M11 cell walls (Figure 4G), thus further supporting the lack of chitin involvement in M11‐host interactions.

Other components of M11 cell walls that could be eliciting the plant immune response are β‐glucans and glycoproteins. β‐glucans are known to elicit immune responses in a wide range of plants (Fesel and Zuccaro 2016), including *Arabidopsis* (Melida et al. 2018; Rebaque et al. 2021). The machinery required for the production of β‐glucans with β‐1,3‐ and β‐1,6‐ linkages was present in the M11 genome (Table S10). The presence of these polysaccharides in *Taphrina* is also supported by biochemical studies (Petit and Schneider 1983). Cell walls of M11 also contain glycoproteins, which could be recognised by plant immune receptors. Cell wall monosaccharide analysis indicates that *Taphrina* walls contain mannose, rhamnose, and galactose (Petit and Schneider 1983; Sjamsuridzal et al. 1997; Schwerdt et al. 2021). These could be arranged in galactomannans and rhamnomannans as seen in *Rhynchosporium secalis*, a filamentous plant‐pathogen in the Pezizomycotina (Ascomycota) (Pettolino et al. 2009). Both galactomannans and rhamnomannans are recognised by the innate immunity of animals (Barreto‐Bergter and Figueiredo 2014) and could possibly also be recognised by plants. *Taphrina* cell walls could also contain simple mannose polysaccharides. Mannopeptides (protease digested mannan glycoproteins) have been previously demonstrated to elicit immune responses in tomato cell cultures (Grosskopf et al. 1991). The presence of mannose and galactose containing glycans in M11 cell walls was supported by cell wall biosynthesis enzyme predictions (Table S10). *Taphrina* M11 *N‐*glycosylation and *O‐*glycosylation enzymes are similar to those of *S. pombe* (Ohashi et al. 2020), including the lack of M‐Pol I complex protein Van1 and the presence of various galactosyltransferase homologues. These results suggest a rich variety of potential PAMPs is present in the M11 cell wall.

The viability of a genetics approach with this system was tested in a pilot screen for receptors involved in M11 cell wall perception. The reverse genetic screen suggested the tested candidates did not function as receptors for *Taphrina* cell wall PAMPs. The tested candidates included a selection of LecRKs (L‐, G‐, and malectin‐type), LecPs (malectin‐ and jacalin‐type), and known receptors or co‐receptors (Table S1), all of which were not different from wild type, with one exception. The BAK1 co‐receptor was required for immune responses to *Taphrina* cell walls (Figure 4G, Figure S6). BAK1 typically associates with leucine‐rich repeat (LRR) receptor‐like kinases and LRR receptor‐like proteins (LRR‐RLKs/RLPs) (Yasuda et al. 2017) suggesting further candidates for M11 cell wall perception. BAK1 was found to form a complex with LecRK‐VI.2 (Wang, Huang, et al. 2019), thus its involvement with other LecRKs remains possible. All the results above, taken together, support that M11 is pathogenic on *Arabidopsis* and illustrate the value of a genetically tractable model system for plant–yeast interactions.

*Taphrina* are well documented as producers of the plant hormones auxin and cytokinin (Kern and Naef‐Roth 1975; Cissé et al. 2013; Tsai et al. 2014; Streletskii et al. 2016, 2019; Wang et al. 2016). Auxin and cytokinin production are widely believed to be responsible for the dramatic tumour symptoms typical of *Taphrina* species, although this has never been formally tested. Here, we show activation of plant hormone responses during infection with M11 *Taphrina* (Figure 5). Auxin response was activated slightly and non‐specifically in response to both pathogenic and non‐pathogenic *Taphrina* species. *Arabidopsis* cytokinin response was specifically activated in response to the pathogenic M11 strain, but not the non‐host control. Cytokinins are key plant developmental hormones that promote cell division (Argueso et al. 2009) and are also known to be produced by several plant associated microorganisms, including other tumour inducing pathogens (Pertry et al. 2010). Several studies have presented evidence of cytokinin production in *Taphrina* species (Sommer 1961; Barthe and Bulard 1974; Streletskii et al. 2019).

The auxin and cytokinin biosynthesis genes were examined in the M11 genome. The key enzymes necessary for cytokinin production were present in the M11 genome (Table S8), further supporting the hypothesis that the observed activation of cytokinin signalling in *Arabidopsis* may be of microbial origin. The enzymes necessary for auxin production through three different routes: the indole‐3‐acetamide, indole‐3‐pyruvate, and tryptamine pathways (Tables [Link], [Link]), were found in M11. To our knowledge, this is the first study in which the tryptophan monooxygenase and indole acetamide hydrolase pathways have been found in *Taphrina*. In previous studies, this pathway has not been detected in *Taphrina* species (Tsai et al. 2014). In another study, only the two genes of the indole‐3‐pyruvate pathway were found in the genome of *T. deformans* (Cissé et al. 2013). The function of these multiple auxin biosynthesis pathways remains unknown. Models for future testing are suggested by the multiple roles played by auxin in microbes (Spaepen and Vanderleyden 2011), including subversion of host immunity (Fu and Wang 2011; Naseem and Dandekar 2012; Ma and Ma 2016), promoting host growth (Ahmad et al. 2008; Contreras‐Cornejo et al. 2009), regulation of fungal development (Chanclud and Morel 2016), and adaptation to the phyllosphere (Vorholt 2012; Kemler et al. 2017).

### 4.3 The Distribution and Ecology of M11 *Taphrina*

Remarkably, *T. tormentillae* occurred frequently in various environments and several plants outside its typical host range, including *Arabidopsis* (Table 2) (Wang et al. 2016; Almario et al. 2022; Brachi et al. 2022; Ruraz et al. 2023). Considering the other *T. tormentillae* strains used here, PYCC 5727 was isolated from the known host, *P. canadensis*, and PYCC 5705 was isolated from birch ( *Betula pendula* ). This characteristic is fairly common; several *Taphrina* species have been found on plants other than their hosts. *T. deformans* and an undefined *Taphrina* species have been found to act as hub microbes in the phyllosphere of romaine lettuce and switchgrass, respectively (VanWallendael et al. 2022; Brandl et al. 2023). We have found *T. padi* on peach (Sipilä and Overmyer, unpublished results). The presence of a *Taphrina* species was correlated with dysbiosis in Chinese rye grass (Qian et al. 2024). Finally, *T. carpini*, *T. padi*, and *T. epiphylla* were also common on *Arabidopsis*, in addition to *T*. *tormentillae* (Almario et al. 2022; Brachi et al. 2022). These findings taken together have implications for the isolation of new *Taphrina* species, which are frequently named after their host of origin, assuming a single host. With easily available environmental sequencing data, distribution should be considered when characterising new species. However, such analysis must be mindful of potential artefacts, such as detection of dead cells, PCR bias, sequencing errors, among others, as previously discussed (Lücking et al. 2020). Additionally, furthering our understanding of *Taphrina* species will require overcoming the limited sampling. Many *Taphrina* species have been described using a limited number of strains and DNAs, a problem that has been discussed for other rare fungi (Kachalkin et al. 2019). Additionally, few live *Taphrina* cultures remain publicly available for future research, with many described species having no strains available. *Taphrina* sequence data are similarly limited, with only rDNA and protein coding sequences available from the few cultured strains and draft genome sequences from only eight species (Cissé et al. 2013; Tsai et al. 2014; Wang et al. 2020).

Some *Taphrina* appear to be specialised as non‐pathogenic phyllosphere residents with a broad host‐range (Inacio et al. 2004). These species have characteristic physiological properties, an expanded carbon source utilisation profile, and most have the ability to assimilate inositol. M11 does not utilise inositol, but its carbon utilisation profile is intermediate between broad host‐range and typical *Taphrina* species (Table S3), suggesting a continuum in the breadth of host range for the phylloplane‐resident yeast form of *Taphrina* species. Remarkably, M11 is a heritable microbiome hub (Almario et al. 2022; Brachi et al. 2022) that correlates with enhanced *Arabidopsis* fitness when present as a phyllosphere microbiome resident. However, access to the phylloplane of multiple plants may open the possibility of developing new hosts for the infectious hyphal life phase. Given the subtle disease M11 caused on *Arabidopsis*, this interaction may represent an intermediate in this process.

## 5 Conclusions

The ability of M11 *Taphrina* to cause disease on *Arabidopsis* was demonstrated, establishing a genetically tractable model system for yeast‐plant interactions. A pilot reverse genetic screen with this system implicated the BAK1 co‐receptor and excluded known chitin receptors in *Arabidopsis* perception of M11 cell walls. Analysis of the M11 genome has identified systems involved in host interactions, including multiple potentially immunoactive cell‐wall components. A broad distribution, including multiple plant species not related to their known host, was demonstrated for *Taphrina* strains similar to M11, suggesting *Taphrina* interact in diverse ecological roles with multiple plants. This genetically tractable model system opens the possibility to address questions of the complex multiple ecological functions of *Taphrina*.

## Author Contributions

**Agate Auzane:** investigation, funding acquisition, supervision, formal analysis, validation, visualization, writing – review and editing, writing – original draft. **Margaretta Christita:** writing – review and editing, writing – original draft, investigation, formal analysis, visualization, funding acquisition, validation. **Kai Wang:** writing – review and editing, investigation, formal analysis, data curation, visualization. **Timo Sipilä:** data curation, investigation, validation, writing – review and editing. **Sitaram Rajaraman:** writing – review and editing, formal analysis, investigation, software, validation. **Gugan Eswaran:** writing – review and editing, investigation. **Jasmin Kemppinen:** writing – review and editing, investigation, formal analysis, validation. **Alejandro De La Fuente:** writing – review and editing, investigation. **Klaas Bouwmeester:** resources, writing – review and editing. **Petri Auvinen:** writing – review and editing, supervision, funding acquisition. **Lars Paulin:** writing – review and editing, validation, investigation. **Jarkko Salojärvi:** writing – review and editing, software, funding acquisition, supervision, validation. **Maija Sierla:** writing – review and editing, funding acquisition, supervision, validation. **Mikael Broché:** writing – review and editing, resources, supervision, validation. **Kirk Overmyer:** writing – original draft, writing – review and editing, conceptualization, formal analysis, project administration, funding acquisition, investigation, supervision, validation, visualization.

## Conflicts of Interest

The authors declare no conflicts of interest.

## Supporting information


**DATA S1.** Supporting Information.

**DATA S2.** Supporting Information.

**DATA S3.** Supporting Information.

**DATA S4‐S8.** Supporting Information.

**FIGURE S1.** Leaf curling index and leaf bending index measurement.

**FIGURE S2.** Colony colour differences in *T. tormentillae* strains.

**FIGURE S3.** Expression of promoter‐RUBY lines after exposure to M11 cell wall extract, all replicates.

**FIGURE S4.** Root growth inhibition assay with lectin receptor kinase/protein mutants.

**FIGURE S5.** Photos of mutant lines with reduced chlorosis and shoot growth inhibition.

**FIGURE S6.** Shoot weight and chlorophyll quantification of known receptor/co‐receptor mutants.

**FIGURE S7.** Phylogeny of CHS genes from *Taphrina* species and fungal model organisms from the phylum Ascomycota.

**FIGURE S8.** Sequence conservation in class I CHS genes from *Taphrina* species.

**FIGURE S9.** Sequence conservation in class II CHS genes from *Taphrina deformans*.

**FIGURE S10.** Sequence conservation in class III CHS genes from *Taphrina* species.

**TABLE S1.** Receptor‐like kinase (RLK) and receptor‐like protein (RLP) lines included in the reverse genetic screen.

**TABLE S2.** Primers used for genotyping and qPCR.

**TABLE S3.** Carbon assimilation by *Taphrina tormentillae* strains.

**TABLE S4.** *Taphrina tormentillae* distribution.

**TABLE S5.** *Taphrina* strain M11 genome assembly statistics.

**TABLE S6.** Comparison of genome statistics and candidate effector proteins.

**TABLE S7.** Comparison of orthologs in selected Taphrinomycotina species.

**TABLE S8.** Auxin and cytokinin biosynthesis pathways in *Taphrina* strain M11.

**TABLE S9.** M11 *Taphrina* auxin (IAA: indole‐3‐acetic acid) biosynthesis pathways data.

**TABLE S10.** Putative cell wall biosynthesis and chitin modification genes in *Taphrina* strain M11.

**TABLE S11.** Promotor‐RUBY line immunity marker genes.

## Data Availability

The data that supports the findings of this study are available in the supplemental material of this article or can be found in the public databases under the accessions listed herein. Any data not found from the above sources are available from the corresponding author upon reasonable request.
