# Supplementary material for: A Novel Pathosystem With the Model Plant Arabidopsis thaliana for Defining the Molecular Basis of Taphrina Infections
Source: Environ Microbiol Rep. 2025 Jun 10;17(3):e70118. doi: 10.1111/1758-2229.70118 (PMC12152203; doi:10.1111/1758-2229.70118)
Supplement: Supplementary file 5 — FIGURE S1. Leaf curling index and leaf bending index measurement. [file EMI4-17-e70118-s009.pdf]

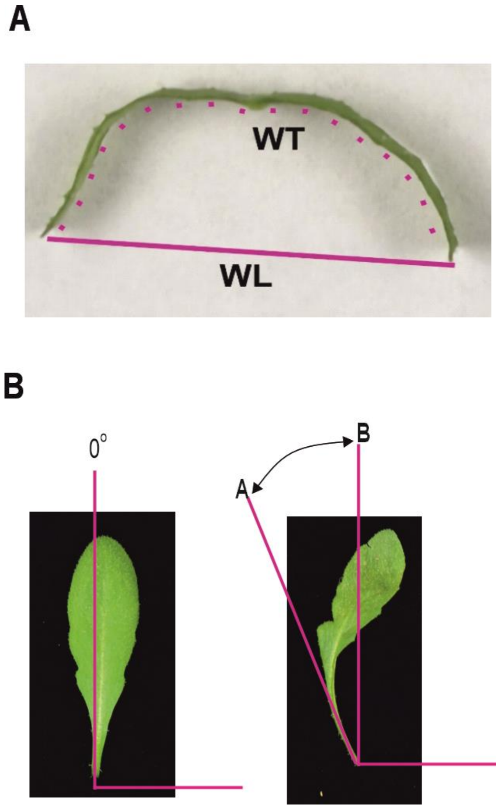

Figure S1. **Leaf curling index and leaf bending index measurement.** Leaf curling **(A)** was measured as in (Booker et al., 2004), briefly this calculates the ratio of the leaf width (W_L) to the total width (W_T), so that leaves with greater curling will have smaller values for the leaf curling index. Leaf bending **(B)** was quantified by measuring the angle between a line following the base of the petiole and a second line defined by points at the middle and tip of the leaf, such that leaves with greater bending will have higher values.
