# Supplementary material for: A Novel Pathosystem With the Model Plant Arabidopsis thaliana for Defining the Molecular Basis of Taphrina Infections
Source: Environ Microbiol Rep. 2025 Jun 10;17(3):e70118. doi: 10.1111/1758-2229.70118 (PMC12152203; doi:10.1111/1758-2229.70118)
Supplement: Supplementary file 6 — FIGURE S2. Colony colour differences in T. tormentillae strains. [file EMI4-17-e70118-s011.pdf]

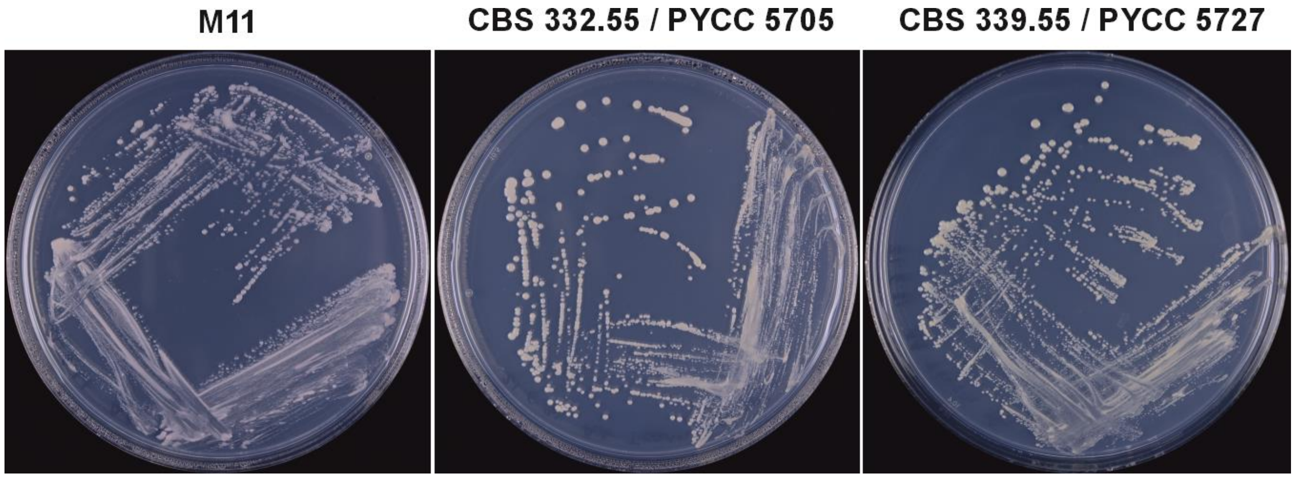

Figure S2. Colony colour differences in *T. tormentillae* strains. *T. tormentillae* cells were grown on 0.2 x PDA media and photographed after 18 days. Used *T. tormentillae* strains from left to right: strain M11 (*Arabidopsis* isolate), strain PYCC 5705/CBC332.55 (birch isolate), PYCC 5727/CBS339.55 (*Potentilla* isolate).
