# Supplementary material for: A Novel Pathosystem With the Model Plant Arabidopsis thaliana for Defining the Molecular Basis of Taphrina Infections
Source: Environ Microbiol Rep. 2025 Jun 10;17(3):e70118. doi: 10.1111/1758-2229.70118 (PMC12152203; doi:10.1111/1758-2229.70118)
Supplement: Supplementary file 7 — FIGURE S3. Expression of promoter‐RUBY lines after exposure to M11 cell wall extract, all replicates. [file EMI4-17-e70118-s024.pdf]

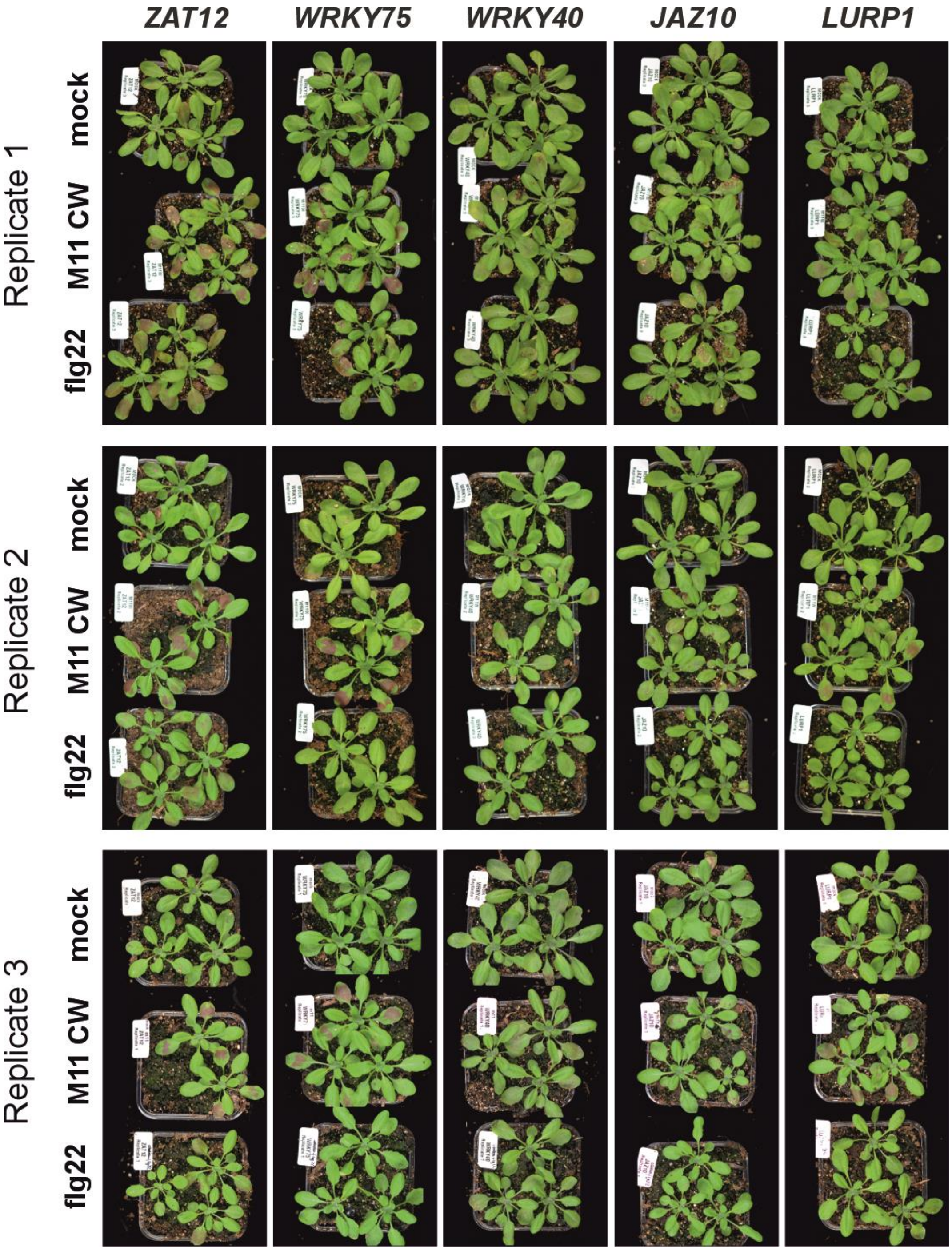

Figure S3. Expression of promoter-RUBY lines after exposure to M11 cell wall extract, all replicates. 21-24 day old RUBY lines were treated with either M11 cell wall extract (M11 CW), 200 nM flg22 (positive control) or water (negative control). *ZAT12, WRKY75* – reactive oxygen species (ROS) marker genes; *WRKY40* – pattern triggered immunity (PTI) marker gene; *JAZ10* – jasmonic acid marker gene; *LURP1* – pathogen marker gene. Activation of the promoter results in appearance of red colour.
