# Supplementary material for: A Novel Pathosystem With the Model Plant Arabidopsis thaliana for Defining the Molecular Basis of Taphrina Infections
Source: Environ Microbiol Rep. 2025 Jun 10;17(3):e70118. doi: 10.1111/1758-2229.70118 (PMC12152203; doi:10.1111/1758-2229.70118)
Supplement: Supplementary file 8 — FIGURE S4. Root growth inhibition assay with lectin receptor kinase/protein mutants. [file EMI4-17-e70118-s023.pdf]

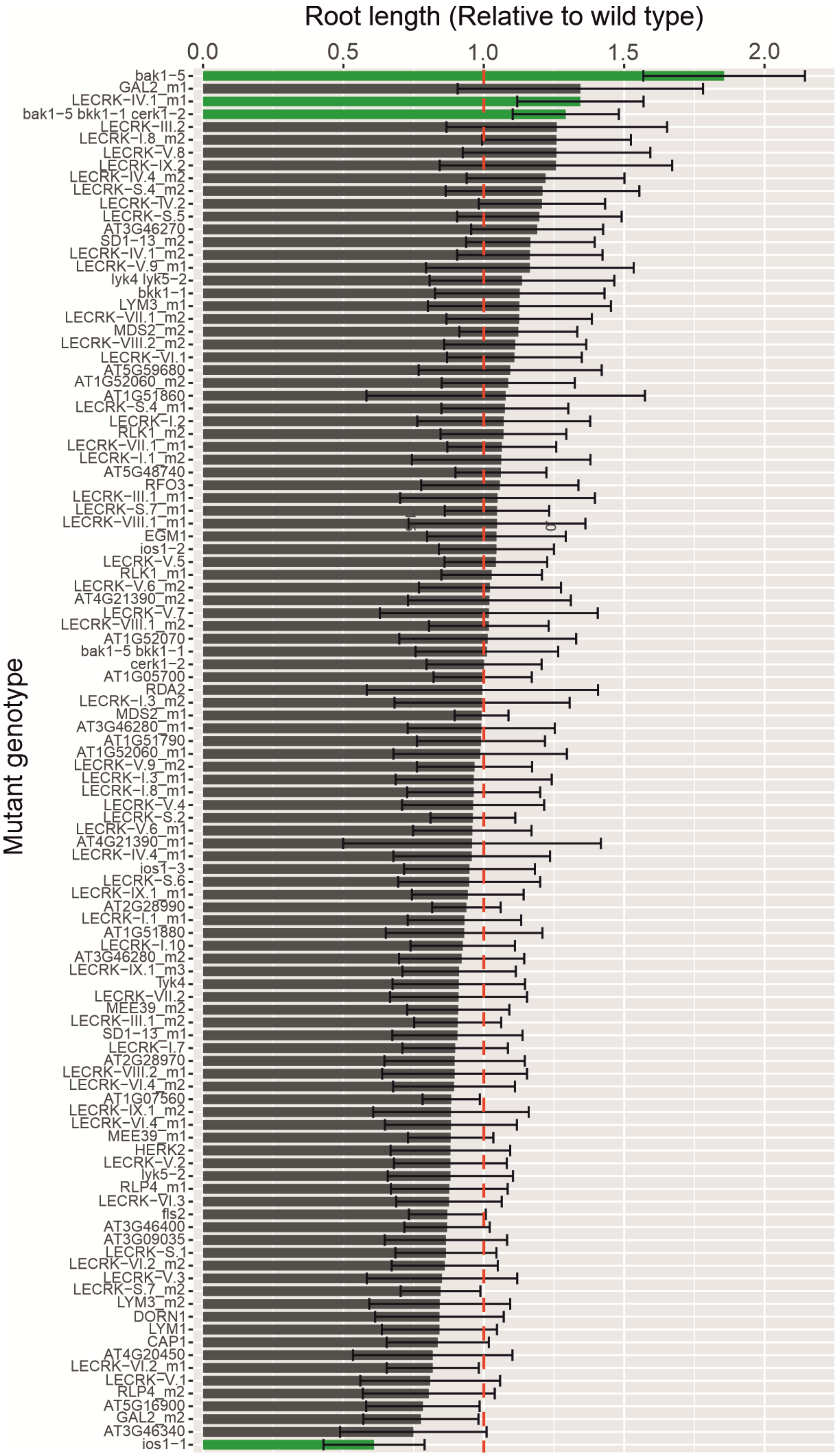
Root length (Relative to wild type)
0.0
0.5
1.0
1.5
2.0
Mutant genotype
bak1−5
GAL2_m1
LECRK−IV.1_m1
bak1−5 bkk1−1 cerk1−2
LECRK−III.2
LECRK−I.8_m2
LECRK−V.8
LECRK−IX.2
LECRK−IV.4_m2
LECRK−S.4_m2
LECRK−IV.2
LECRK−S.5
AT3G46270
SD1−13_m2
LECRK−IV.1_m2
LECRK−V.9_m1
lyk4 lyk5−2
bkk1−1
LYM3_m1
LECRK−VII.1_m2
MDS2_m2
LECRK−VIII.2_m2
LECRK−VI.1
AT5G59680
AT1G52060_m2
AT1G51860
LECRK−S.4_m1
LECRK−I.2
RLK1_m2
LECRK−VII.1_m1
LECRK−I.1_m2
AT5G48740
RFO3
LECRK−III.1_m1
LECRK−S.7_m1
LECRK−VIII.1_m1
EGM1
ios1−2
LECRK−V.5
RLK1_m1
LECRK−V.6_m2
AT4G21390_m2
LECRK−V.7
LECRK−VIII.1_m2
AT1G52070
bak1−5 bkk1−1
cerk1−2
AT1G05700
RDA2
LECRK−I.3_m2
MDS2_m1
AT3G46280_m1
AT1G51790
AT1G52060_m1
LECRK−V.9_m2
LECRK−I.3_m1
LECRK−I.8_m1
LECRK−V.4
LECRK−S.2
LECRK−V.6_m1
AT4G21390_m1
LECRK−IV.4_m1
ios1−3
LECRK−S.6
LECRK−IX.1_m1
AT2G28990
LECRK−I.1_m1
AT1G51880
LECRK−I.10
AT3G46280_m2
LECRK−IX.1_m3
lyk4
LECRK−VII.2
MEE39_m2
LECRK−III.1_m2
SD1−13_m1
LECRK−I.7
AT2G28970
LECRK−VIII.2_m1
LECRK−VI.4_m2
AT1G07560
LECRK−IX.1_m2
LECRK−VI.4_m1
MEE39_m1
HERK2
LECRK−V.2
lyk5−2
RLP4_m1
LECRK−VI.3
fls2
AT3G46400
AT3G09035
LECRK−S.1
LECRK−VI.2_m2
LECRK−V.3
LECRK−S.7_m2
LYM3_m2
DORN1
LYM1
CAP1
AT4G20450
LECRK−VI.2_m1
LECRK−V.1
RLP4_m2
AT5G16900
GAL2_m2
AT3G46340
ios1−1

Figure S4. Root growth inhibition assay with lectin receptor kinase/protein mutants. Mutants were grown on plates containing M11 cell walls and their relative root length to wild type plants plotted**.** For full descriptions of the screened mutants see Table S1.
