# Supplementary material for: A Novel Pathosystem With the Model Plant Arabidopsis thaliana for Defining the Molecular Basis of Taphrina Infections
Source: Environ Microbiol Rep. 2025 Jun 10;17(3):e70118. doi: 10.1111/1758-2229.70118 (PMC12152203; doi:10.1111/1758-2229.70118)
Supplement: Supplementary file 9 — FIGURE S5. Photos of mutant lines with reduced chlorosis and shoot growth inhibition. [file EMI4-17-e70118-s006.pdf]

Replicate 1 SD1-13_m1 (SALK099776c)

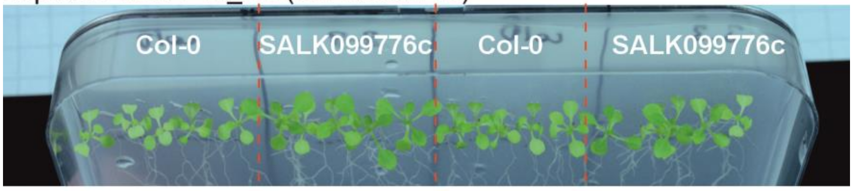

Replicate 2 SD1-13_m1 (SALK099776c)

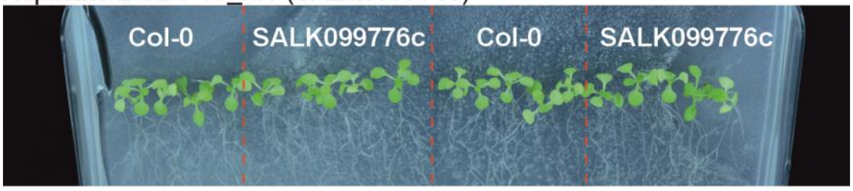

Replicate 1 SD1-13_m2 (SALK026338c)

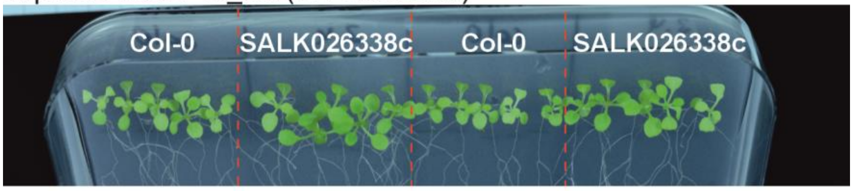

Replicate 2 SD1-13_m2 (SALK026338c)

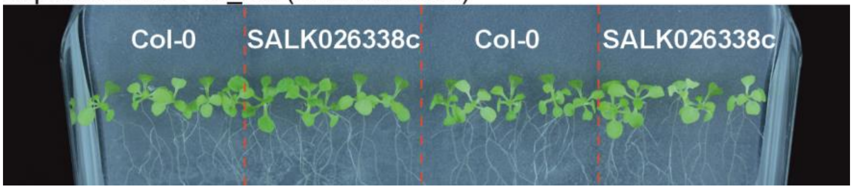

Figure S5. Photos of mutant lines with reduced chlorosis and shoot growth inhibition. Mutants were grown on plates contain M11 cell walls in the primary reverse genetic screen and genotypes displaying visible phenotypes photographed.
