# Supplementary material for: A Novel Pathosystem With the Model Plant Arabidopsis thaliana for Defining the Molecular Basis of Taphrina Infections
Source: Environ Microbiol Rep. 2025 Jun 10;17(3):e70118. doi: 10.1111/1758-2229.70118 (PMC12152203; doi:10.1111/1758-2229.70118)
Supplement: Supplementary file 10 — FIGURE S6. Shoot weight and chlorophyll quantification of known receptor/co‐receptor mutants. [file EMI4-17-e70118-s025.pdf]

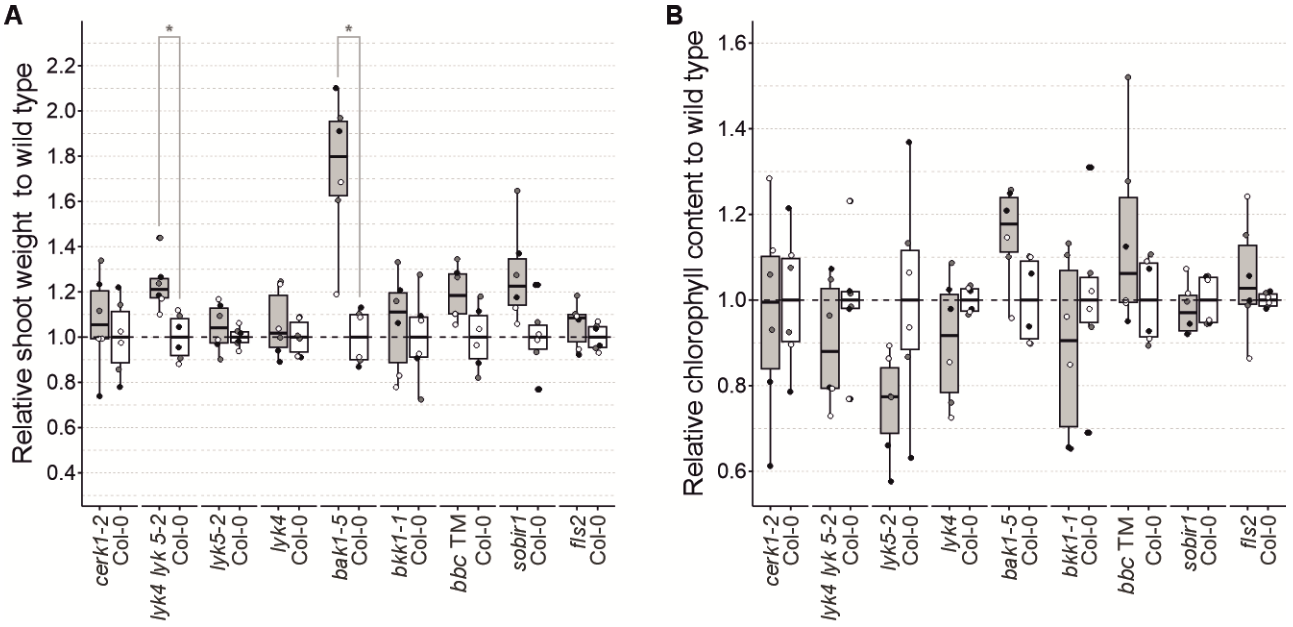

Figure S6. Shoot weight and chlorophyll quantification of known receptor/co-receptor mutants. Mutants were grown on plates contain M11 cell walls in the primary reverse genetic screen. Chlorosis was quantified with a chlorophyll content assay. Mutant abbreviation: *bbc* TM, *bak1-5 bkk1-1 cerk1-2* triple mutant
