# Supplementary material for: A Novel Pathosystem With the Model Plant Arabidopsis thaliana for Defining the Molecular Basis of Taphrina Infections
Source: Environ Microbiol Rep. 2025 Jun 10;17(3):e70118. doi: 10.1111/1758-2229.70118 (PMC12152203; doi:10.1111/1758-2229.70118)
Supplement: Supplementary file 11 — FIGURE S7. Phylogeny of CHS genes from Taphrina species and fungal model organisms from the phylum Ascomycota. [file EMI4-17-e70118-s013.pdf]

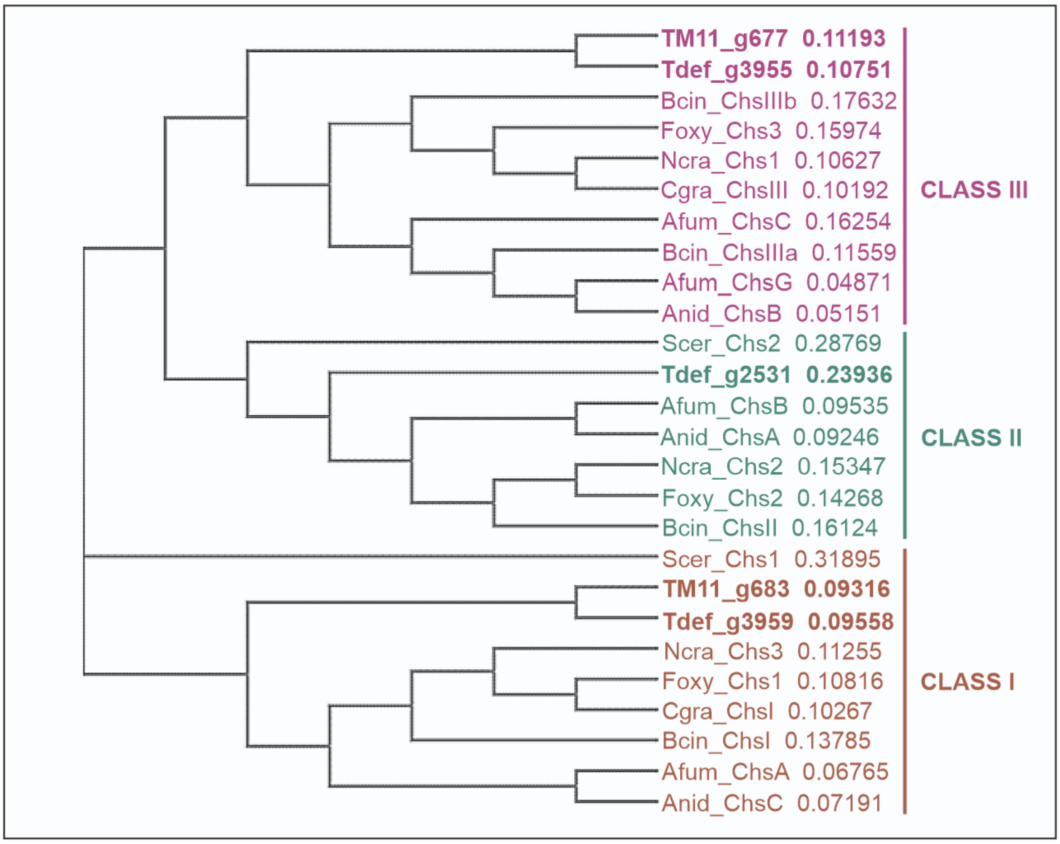

Figure S7. Phylogeny of CHS genes from *Taphrina* species and fungal model organisms from the phylum *Ascomycota*.
