# Supplementary material for: A Novel Pathosystem With the Model Plant Arabidopsis thaliana for Defining the Molecular Basis of Taphrina Infections
Source: Environ Microbiol Rep. 2025 Jun 10;17(3):e70118. doi: 10.1111/1758-2229.70118 (PMC12152203; doi:10.1111/1758-2229.70118)
Supplement: Supplementary file 12 — FIGURE S8. Sequence conservation in class I CHS genes from Taphrina species. [file EMI4-17-e70118-s005.pdf]

Figure S8. Sequence conservation in class I CHS genes from *Taphrina* species. Multiple sequence alignment was performed with Clusal Omega. Conserved pfam domains are higlighted as follows: pink, chitin synthase 1 N-terminal (pfam08407); yellow, chitin synthase 1 (pfam01644); blue, partial chitin synthase 2 (pfam03142). Locations of the first two pfam domains are from NCBI database. The coordinates of chitin synthase 2 are from Li *et al*., 2016, because at NCBI database they were supported by alignment of only 7 sequences. Red boxes indicate conserved functional motifs listed in Li *et al*., 2016: 1, ligand binding; 2, metal ion binding site; 3, donor saccharide binding; 4, acceptor saccharide binding; 5, product binding. Blue boxes indicate conserved sequence patterns defined by Li *et al*., 2016. Blue colored amino acids in CHS sequences from *Taphrina* species do not match the conserved sequence patterns.

```
S_cer_Chs1  MSDQNNRSRNEYHSNRKNEPSYELQNAHSGLFHSSNEELTNRNQRYTNQNASMGSFTPVQ 60
T_M11_g683  ------------------------------------------------------------ 0
T_def_g3959 ------------------------------------------------------------ 0
N_cra_Chs3  ------------------------------------------------------------ 0
F_oxy_Chs1  ------------------------------------------------------------ 0
C_gra_ChsI  ------------------------------------------------------------ 0
A_fum_ChsA  ------------------------------------------------------------ 0
A_nid_ChsC  ------------------------------------------------------------ 0
B_cin_ChsI  ------------------------------------------------------------ 0


S_cer_Chs1  SLQFPEQSQQTNMLYNGDDGNNNTINDNERDIYGGFVNHH-RQR-PPP---ATAEYNDVF 115
T_M11_g683  ---------MSNLNWPDTD--YR---RN-N-------------------RSP-GVYNNNY 25
T_def_g3959 ------------MSWQNGQ--YS---DG-GHYDNGHN----------PNYPQ-TQYDQHY 31
N_cra_Chs3  -----------------------------------MDPR--MHTAPPAGHPIQPGYQLED 23
F_oxy_Chs1  -----------------MDPRYG---AQ-----PQQHPH--PHRTPSPGQPLQQGYQLDD 33
C_gra_ChsI  -----------------------------------MDPR--YHRTPSPGQPLQHGYQLED 23
A_fum_ChsA  ------------MSYNRLD-HYG---ED-GDRSPTMNPQHLADRTPSPGRPL-NTYQLSD 42
A_nid_ChsC  ------------MSYNRLGDPYG---DDRDARSPIMNPSSLSNRSPSPGRPL-DGYQLSD 44
B_cin_ChsI  ------------MSYNRLDPA-G---QGQDDYYN-MN-----NRHQS--PHP-QGYQLED 35
                                                                   *:

S_cer_Chs1  N---TNS--QQLPSEHQYNNVPSYPLPSINVIQTTPELIHNGSQTMATPIERPFFNENDY 170
T_M11_g683  GPYDPYRPENHSSTS--FNH-VT-------------------S----------------- 46
T_def_g3959 DPYAPYSQQQHNQSNSSFGYTPS-------------------V----------------- 55
N_cra_Chs3  NPYHHQQ-------------------------------------GFDIPAGP-------- 38
F_oxy_Chs1  NPFDDGRYGQYGPS----------------------------QQHLAMPSGP-------- 57
C_gra_ChsI  NPFNNNAAYQPPPQHDPYGHTSP-------------------HQQLDVPMGP-------- 56
A_fum_ChsA  NPYVH------------------------------------------------------- 47
A_nid_ChsC  APYGHH------------------------------------------------------ 50
B_cin_ChsI  APYGRPNTTSPGPG----------------------------MHNLEIPMGP-------- 59


S_cer_Chs1  YYNNRNSRTSPSIASSSDGYADQEARPILEQPNNNMNS-----GNIPQYHDQPFGYNNGY 225
T_M11_g683  ----------PI-YDAHD--PLNEAYPLQSYPQGLTASHPY------DATPSPGGMDPYT 87
T_def_g3959 ----------AR-YTLED--PQEESYPLQQYPQGLTASNPY------THTPSPGGMSPYG 96
N_cra_Chs3  --------GR---YSPGDALHIQTPQPIEGMGG---------YNAPGQHYTPDYAVNPEE 78
F_oxy_Chs1  --------DQHRLPTPSDHLNLNAAQSVDNLSG---------YGPP-----GDYAVNPEA 95
C_gra_ChsI  --------P-ARYGTPSDQLPLNAAHSVSNLSG---------YDTPVNH--GDYGVNPEA 96
A_fum_ChsA  ---------DHLQMPSSDRLAEQPTYSVERIPNSYGHNEAYEQRHHEQYPAYDYAVNPEA 98
A_nid_ChsC  ---------HHIEMPSSDRLAEQPTYSVERIPQSYGHNEAYEAQ-HQHYPGYEYSVDPEA 100
B_cin_ChsI  --------GAHRIGTPSDQLQAQPSYSVEHLDQNQYHQRMSL--NPSQSYDSEYSLDPNA 109
```

```
                               *    :    :                          . .

S_cer_Chs1    HGLQAKDYYDDPEGGYIDQRGDDYQINSYLGRNGEMVDPYDYENSLRHMTPMERREYLHD 285
T_M11_g683    HP-NDS-YFH--------DQG--ADLGYG---Q----PH---L--------------QDE 111
T_def_g3959   HG-HDSSYFH--------DG----DLGYQTGHD----PS---R--------------PDD 122
N_cra_Chs3    H---HDAYYNQPYEPQVGHDPYAAAP-----TP----PVAGYQ--------------AHD 112
F_oxy_Chs1    H---HDAYYNQPYEPRPQQQPYDQG-------Y----DQEYDQ--------------PYD 127
C_gra_ChsI    H---HDAYYNQPYEPSPHDPSVPYD-------Q----PTG-YS--------------EYD 127
A_fum_ChsA    H---HDAYYTQPYEPTVTPQ-DDYDLGQYHEQH----QPY-----------------QD 132
A_nid_ChsC    H---HDAYYTQPYQPTVTPGHDDYDLGQYPGHQ----HSY-----------------QD 135
B_cin_ChsI    H---HDAYYQPPYQPSPHEEH---PLQNYAPGQ----DPYAYN--------------DDD 145
              *    . *:                                                  :

S_cer_Chs1    DSRPVNDGKEELDSVKSGYSHRDLGEYDKDDFSRDDEYDDLNTIDKLQFQANGVPASSSV 345
T_M11_g683    MQSPLLDQFP-----------------------------------K-----SL---HDGL 128
T_def_g3959   MQSPLLEQFP-----------------------------------K-----EL---HDGP 139
N_cra_Chs3    DQRPMLMHTD-----------------------------------S-----QV------- 125
F_oxy_Chs1    DHRPMLQHQP-----------------------------------S-----DA------- 140
C_gra_ChsI    DNRPMLPHQD-----------------------------------T-----TD------- 140
A_fum_ChsA    DQVPILQPEN-----------------------------------P-----FG-PD---- 147
A_nid_ChsC    D-EPILQPED-----------------------------------P-----FQAQN---- 150
B_cin_ChsI    DHQPILQSHE-----------------------------------P-----YGPDPHSAS 165
                 *:

S_cer_Chs1    SSIGSKESDIIVSNDNLTANRALKRSGTEIRKFKLWNGNFVFDSPISKTLLDQYATTTEN 405
T_M11_g683    PSPGEPDPVMT-PYAQVPPVGAHPRRWKTIKRVELYNGNLVLDCPVPQKLLATLPIK--- 184
T_def_g3959   PTPGSVTRHTT-GYAQPPPMGAQPRRYKTMKRVELYKGNLVLDCPIPPKLLATLPIK--- 195
N_cra_Chs3    --GQSDPYHDE-PQ--PPTNNAPIKRWKTVKQVLLYRGNLVLDCPIPPKLLNQLPHG--- 177
F_oxy_Chs1    --P-SEPYQD------QPQQGGGIKRWKTVKQVLLYRGNLVLDCPVPPVLLQQNPHG--- 188
C_gra_ChsI    --G-Y-QDNP------TPQPAGGLKRWKTVKQVLLYRGNLVLDCPVPPRLLNQIPHG--- 187
A_fum_ChsA    --PYSEEYHDD-PAAVPTPSPAPIRRWKTVKEVQLFHGNLVLDCPIAPKLLSQVPHAE-- 202
A_nid_ChsC    --PYSDDYQED-MTIAPTPSPAPLRRWKTVKEVQLFQGNLVLDCPIAPKLLNQIPHAE-- 205
B_cin_ChsI    GTDYKGGYDGT-VQSPSATPVPALRRYKTVKEVQLFNGNLVLDCPIPPKLLNQVNHAP-- 222
                                      :  . ::.. *:.**:*:*.*:   **

                                                             1.
S_cer_Chs1    ANTLPNEFKFMRYQAVTCEPNQLAEKNFTVRQLKYLTPRETELMLVVTMYNEDHILLGRT 465
T_M11_g683    ---EGREFTHMRYTAATGDPSEFVSRGFTLRQKLYQPSRQTELFIVITMYNENEILFART 241
T_def_g3959   ---DGREFTHMRYTAATGDPSDFARKGFTLRQALYQPQRQTELFIVITMYNENEILFART 252
N_cra_Chs3    ---ERDEFTHMRYSAATCDPSEFYEENFTLRQKLFSKPRHTELFIVVTMYNEDEILFART 234
F_oxy_Chs1    ---ERDEFTHMRYSAATCDPNDFYDHDFTLRQRLFTKPRHTELFIVVTMYNEDDILFART 245
C_gra_ChsI    ---ERDEFTHMRYTAATCDPNYFYDDNFTLRQKLFSKPRHTELFIVVTMYNEDEILFART 244
A_fum_ChsA    -PPGRDEFTHMRYSAATCDPADFYEERFTLRQKLFAKPRHTELFIVITMYNEDDFLFART 261
A_nid_ChsC    -NGQRDEFTHMRYSAATCDPKDFFEERFTLRQKLFAKPRHTELFIVVTMYNEDDFLFART 264
B_cin_ChsI    -PPERDEFTHMRYSAATCDPSEFFEERFTLRQKLFAKPRHTELFIVVTMYNEDDVLFART 281
                    **..*** *.* :*  :    **:**  :   *.***::*:*****:..*:.**

S_cer_Chs1    LKGIMDNVKYMVKKKNSSTWGPDAWKKIVVCIISDGRSKINERSLALLSSLGCYQDGFAK 525
T_M11_g683    MHSVMKNIAHLVSRTKSRVWGTEGWMKVVVSIVSDGRSKINPRTLSYLAAMGVYQDGIAK 301
T_def_g3959   MHSVMKNIAHLVSRTKSRMWGKDGWMKVVVCVVSDGRSKINPRTLSYLAAMGVYQDGIAK 312
N_cra_Chs3    MIGVFKNIEYMCKRTESKTWGKDAWKKIVVCVVSDGRAKINPRTRALLAGMGVYQEGIAK 294
F_oxy_Chs1    MTGVFKNIEYMCNRPNSKTWGKDAWKKIVVCVVSDGRSKINPRTKALLAGMGVYQEGIAK 305
C_gra_ChsI    MIGVLKNVEYMCNRKESKTWGKDAWKKIVVCVVSDGRAKINPRTRALLAGMGVYQEGIAK 304
A_fum_ChsA    LIGVFKNIEYMCNRTQSKTWGKDAWKKIVVCVISDGRAKINPRTRAVLAGLGVYQDGIAK 321
A_nid_ChsC    MVGVFKNIEHMCSRTRSKTWGKDAWKKIVVCVISDGRAKINPRTRAVLAGLGCYQDGIAK 324
B_cin_ChsI    MHGVFKNIEFMCTRKDSKTWGKDAWKKIVVCVVSDGRAKINPRTRAVLAGLGVYQDGIAK 341
              : .::.*: .: .:  *  ** :.* *:**.::****:*** *: : *:.:* **:*:**
```

```
S_cer_Chs1    DEINEKKVAMHVYEHTTMINITNISESEVSLECNQGTVPIQLLFCLKEQNQKKINSHRWA 585
T_M11_g683    NVVNNEPVTAHLYEYTTQISINAD--LTF-KGSDRGIVPVQMLFCLKEKNQKKINSHRWF 358
T_def_g3959   NVVNNEPVTAHLYEYTTQISINSD--LTF-KGSDRGIVPVQMLFCLKEKNQKKINSHRWF 369
N_cra_Chs3    QQVNGKDVTAHIYEYTTQVGMTIK--NDVVQLIPK-QQPVQMLFCLKEKNQKKINSHRWF 351
F_oxy_Chs1    QQVNGKDVTAHIYEYTTQTHLQIK--NDVVQLVHR-RQPVQMLFCLKEKNAKKINSHRWF 362
C_gra_ChsI    QQVNGKDVTAHIYEYTSQVGMMIK--NDVVTLVPK-QQPVQMLFCLKEKNQKKINSHRWF 361
A_fum_ChsA    QQVNGKDVTAHIYEYTTQVGLELK--GTQVHIKGRSACPVQMIFCLKEKNQKKINSHRWF 379
A_nid_ChsC    QQVNGKDVTAHIYEYTTQVGMELK--GNQVHLKPRSGVPVQMIFCLKEKNQKKINSHRWF 382
B_cin_ChsI    QQVNGKDVTAHIYEYTTQVGISLK--KDIVTLTPK-QQPVQLLFCLKEKNQKKINSHRWF 398
              : :* : *: *:**:*:   :             :   *:*::*****:* ********

                             2.
S_cer_Chs1    FEGFAELLRPNIVTLLDAGIMPGKDSIYQLWREFR-NPNVGGACGEIRTDLGKRFVKLLN 644
T_M11_g683    FSAFGQVLNPNVCVLLDAGTQPGGDSIYHLWKAFDLNSNVGGACGEIVAMKGPYGKYLLN 418
T_def_g3959   FSAFGEVLVPNVCVLLDAGTQPGGDSIYHLWKSFDLNSNVGGACGEIVAMKGKYGKYLLN 429
N_cra_Chs3    FQAFGRVLDPNICVLIDAGIKPGGSSIYHLWKAFDLEPMCAGACGEIKAMLGTGGKNLIN 411
F_oxy_Chs1    FTAFGRVLDPNICVLLDAGIRPGGSSIYHLWKAFDLEPMCSGACGEIKAMLGTGGKYLLN 422
C_gra_ChsI    FQAFGRVLDPNICVLIDAGIKPGGNSIYHLWKAFDLEPMCAGACGEIKAMLGTGGKHLLN 421
A_fum_ChsA    FQAFGRVLDPNICVLLDAGIKPGRDSIYQLWKAFDVEPMCGGACGEIKVMLSH-GKKLLN 438
A_nid_ChsC    FQAFGRVLDPNICVLLDAGIQPGKDSIYRLWKAFDVEPMCGGACGEIKVMLDH-GKKLFN 441
B_cin_ChsI    FQAFGRVLDPNICVLLDAGIKPGKDSIYHLWKAFDLEPHCAGACGEIKAMLGPGGKNLVN 458
              * .*..:* **: .*:**** ** .***:**: *  :   .****** .  .     *.*

                                             3.
S_cer_Chs1    PLVASQNFEYKMSNILDKTTESNFGFITVLPGAFSAYRFEAVR-----GQPLQKYFYGEI 699
T_M11_g683    PLVAAQNFEYKMSNILDKPLESVFGFISVLPGAFSAYRFAALQNDSVGQGPLAQYFKGEK 478
T_def_g3959   PLVAAQNFEYKMSNILDKPLESVFGFISVLPGAFSAYRFAALQNDSVGQGPLAQYFKGES 489
N_cra_Chs3    PLVATQNFEYKMSNILDKPLESAFGFISVLPGAFSAYRYVALQNDKNGQGPLEKYFAGEK 471
F_oxy_Chs1    PLVAAQNFEYKMSNILDKPLESAFGFISVLPGAFSAYRYVALQNDKNGKGPLEKYFLGET 482
C_gra_ChsI    PLVATQNFEYKMSNILDKPLESAFGFISVLPGAFSAYRYVALQNDKNGQGPLEKYFAGEK 481
A_fum_ChsA    PLVAGQNFEYKLSNILDKPLESAFGFISVLPGAFSAYRYVALQNDKNGQGPLERYFLGEK 498
A_nid_ChsC    PLVAGQNFEYKLSNILDKPLESAFGFISVLPGAFSAYRYIALQNDKNGQGPLERYFLGEK 501
B_cin_ChsI    PLVATQNFEYKMSNILDKPLESAFGFISVLPGAFSAYRYVALQNDKTGNGPLEKYFAGEK 518
              **** ******:******  ** ****:**********: *::       ** :** **

                             4.                                     5.
S_cer_Chs1    MEN-EGFHFFSSNMYLAEDRILCFEVVTKKNCNWILKYCRSSYASTDVPERVPEFILQRR 758
T_M11_g683    MHG-ANAGIFEANMYLAEDRILCFELVAKRKSAWVLHYVKSAYAVTDVPDELPELISQRR 537
T_def_g3959   MHG-ANAGIFEANMYLAEDRILCFELVAKRKSSWVLHYVKSAYAVTDVPDELPELISQRR 548
N_cra_Chs3    LHGG-DAGIFTANMYLAEDRILCFELVTKRNCHWILQYVKSATGETDVPADLTELILQRR 530
F_oxy_Chs1    LHGGSDAGLFESNMYLAEDRILCFELVTKRNCHWILQYVKSATGETDVPDTVTELVLQRR 542
C_gra_ChsI    LEG-AGAGIFTSNMYLAEDRILCFELVTKRNCHWILQYVKSATGETDVPDTVTELVLQRR 540
A_fum_ChsA    MHG-ANAGIFTANMYLAEDRILCFEIVTKRNCRWLLQYVKSSTGETDVPDRMAEFILQRR 557
A_nid_ChsC    MHG-ANAGIFTANMYLAEDRILCFEIVTKRNCRWLLQYVKSSTGETDVPDQMAEFILQRR 560
B_cin_ChsI    MHG-ANAGIFTANMYLAEDRILCFELVSKRNCHWILQYVKSATGETDVPDTMAELILQRR 577
              :..  .  :* :*************:*:*::. *:*:* :*: . ****  : *:: ***

S_cer_Chs1    RWLNGSFFASVYSFCHFYRVWSSGHNIGRKLLLTVEFFYLFFNTLISWFSLSSFFLVFRI 818
T_M11_g683    RWLNGSFFAAVYALWHTFAIWRSDHGFFRKIFFHIEFLYQGISMLFSWFGLGNFFIAFSI 597
T_def_g3959   RWLNGSFFAAVYALWHTFAIWRSDHGIFRKLMFHIEFLYQGISMLFSWFGLGNFFIAFTI 608
N_cra_Chs3    RWLNGSFFAAIYAIVHFHQFFRSDHSFLRKIAFFIEFVFQTVNMIFAWFAIGNFFLVFKI 590
F_oxy_Chs1    RWLNGSFFAAIYAIVHFLDFLRSDHTFLRKFAFFIEFIFNTINMIFAWFAIGNFFLVFKI 602
C_gra_ChsI    RWLNGSFFAGIYAIAHFYEFFRSDHSMLRKLMFFVEFVFNTINLIYAWFAIGNFFLVFKI 600
A_fum_ChsA    RWLNGSFFAAVYAIAHFYQIWRSDHSFMRKFMLLVEFIYQTINMIFAWFNIGNFFLVFHI 617
A_nid_ChsC    RWLNGSFFAAVYAITHFYQLWRSDHSFIRKFMLLIETIYQTINMLFAWFGIGNFFLVFHI 620
B_cin_ChsI    RWLNGSFFAAIYALAHFYQIFRSDHSFLRKIMFLIEFTYQTINMIFAWFALGNFFLVFHI 637
              *********.:*:: *   .  *.* : **: : :*  :  .. : :** :..**:.* *
```

```
S_cer_Chs1    LTVSIALAY-----------HSAFNVLSVIFLWLYGICTLSTFILSLGNKPKSTEKFYVL 867
T_M11_g683    LSKALATNKWTIGPRAGKEIFAPGDVLYTVCTWLYAALIVLCFVLSMGNRPQGSKWAYMG 657
T_def_g3959   LAKALAENKWTSGAKIGRDIFSPGQVLYTVCTWIYAGLVVMCFILSMGNRPQGSKWAYIV 668
N_cra_Chs3    LTTGLGDEKL---------LGTVGQILGVVFAWAYGVTLITCFVLSMGNRPAGSPRLYMG 641
F_oxy_Chs1    LTTSLGDDTL---------LGRTGEILGVVFTWLYGVFLITCFVLSLGNRPAGSGRLYTA 653
C_gra_ChsI    LTTSLGDDNL---------LGRTGEILGVVFTWVYGIALITCFVLAMGNRPAGSGPYYIT 651
A_fum_ChsA    LTTYLGDAEL---------LGTTGKVLGVVFEWLYLATLVTCFVLSLGNRPGGSNKFYMT 668
A_nid_ChsC    LTTYLGDADL---------LGTAGKVLGVVFEWLYLATLVTCFVLSLGNRPGGSNKLYMT 671
B_cin_ChsI    LTTSLGSSDL---------LGNVGVILGVVFEWLYLFTLLTCFILALGNRPQGTNKVYMS 688
              *:  :.                    :* .:   * *     :   *:*::**:*  .:   *

S_cer_Chs1    TCVIFAVMMIYMIFCSIFMSVKSFQNILKN----D----TISFEGLITTEAFRDIVISLG 919
T_M11_g683    TMVFFAILMGYMLFAAGFLSYVSIQSLIYTVDATGNKSFESVWAALRADPIFYQLCISLI 717
T_def_g3959   TMTFFALLMGYMIFAAGFLSYVSIEGLILTIETGGNRSFATIFKAVRSDPIFYQLCISLL 728
N_cra_Chs3    MVVFWAIIFIYLMFAAIYIAVVAIQTDVQ-----K----GLSFTDLFRNELFYTLIVSVV 692
F_oxy_Chs1    MCWFWAIIMIYLLFAAIFIAVKAIIADVN---DAN----GFNFADIFKNKVFYMLIISVM 706
C_gra_ChsI    MVYFWAFIMIYLLFAAVFIAVKAIIADVH---DSN----GFNITDLFKNPVFYTLIISVM 704
A_fum_ChsA    MVYFWIGIMIYLTFAAIFVTVKSIQKEVA---D-N----SFSVGQLFSNSQFFSIFVSLG 720
A_nid_ChsC    MVYLWVFIMIYLAFAAVFVTVRSIQEEVK---D-G----SFTFSTLFTNSTFFSIIVSLG 723
B_cin_ChsI    MVYFWIIIMIYLMFASIFITVKSIQTQLA---K-D----EFNWTDIIKNQIFYTLIISLA 740
                 ::  :: *: *.: :::  ::   :                   :     *  : :*:

S_cer_Chs1    STYCLYLISSIIYLQPWHMLTSFIQYILLSPSYINVLNIYAFCNVHDLSWGTKGAMAN-- 977
T_M11_g683    STYGLYLIASVLYFEPWHMITSFVQYMLISPSYINILNVYAFCNTHDISWGTKGDTGVKT 777
T_def_g3959   STYGLYILSSLLYFEPWHMITSFVQYLLISPSYINILNVYAFCNTHDISWGTKGDSGVKT 788
N_cra_Chs3    STYGIWLIASLLMFDPWHMVTSMVQYMLLSPTYTNVLNVYAFCNTHDISWGTKGDDKPDK 752
F_oxy_Chs1    STFGIWLIASLIMLDPWHMATSLVQYMLLTPTFTNVLNVYAFCNTHDVSWGTKGDDKVEK 766
C_gra_ChsI    STYGIWLIASLLMFDPWHMITSFVQYMLLTPTYTNILNVYAFCNTHDISWGTKGDDKAES 764
A_fum_ChsA    STYVMWLLASLIFLDPWHMFTSFIQYMLLTPTYINVLNIYAFCNTHDITWGTKGDDKAEK 780
A_nid_ChsC    STYVMWFIASIIFMDPWHMFTCFIQYILLTPTYINVLNIYAFCNTHDITWGTKGDDKAEK 783
B_cin_ChsI    STYLLWFISSFLFFDPWHMFTSFLQYLLLTPHISIFSTFTLFCNTHDITWGTKG------ 794
              **: :::::*.: ::**** *.::**:*::*      . ..  ***.**.:*****

S_cer_Chs1    PLGKIN-TTEDGTFKMEVLVSSSEIQANYDKYLKVLNDFDPKSESRPTEPSYDEKKTGYY 1036
T_M11_g683    DLGVVNVSKTDGKLEMAMPTSEVDVDALYSKALTTLQTKEPEKKS---KRDAQTKQEDYY 834
T_def_g3959   DLGVVNVSKTDGKLELAMPTSEVDVDELYMKALKTLQVKEPEAKN---KRDAKTKQEDYY 845
N_cra_Chs3    LP-SVN-TKDGQGK-TD-LPDEGDLNASYERELQVFSRKYVKPVTAPTSAQLEEKQMDYY 808
F_oxy_Chs1    LP-SVN-TKDGTGK-TD-LPDEGDLNAQYQRELAVFAQKHVEVKTTPTPSQLQEKQMDYY 822
C_gra_ChsI    LP-TVS-TKDGSGK-TD-LPDEADLNAQYERELTVFSTKFVKEVKAPTESQLAEAQMDYY 820
A_fum_ChsA    LP-SAN-MKPGGKVDVDIPQDDGDLNAQYEAELAKFAQKPPKETKVI---SEEERQADYY 835
A_nid_ChsC    LP-SAN-LKPGGKVDVNIPQDDGDLNAQYEAELMKFAQKPPKEIKTI---SEEERQADYY 838
B_cin_ChsI    ------------------------------------------------------------ 794

S_cer_Chs1    ANVRSLVIIFWVITNFIIVAVVLETGGIADYIAMKSISTDDTLETAKKAEIPLMTSKASI 1096
T_M11_g683    KAFRTRVVLFWIFSNGVLIGLILGVGGVDQIDATSG------------STS---ASRASV 879
T_def_g3959   KAFRTRVVLFWILTNGALVGVVLGVGGVNQIVTGST------------STS---TSNAST 890
N_cra_Chs3    RGVRSMVVLVWMITNFALCAVVLSTAGLERIDPEEG------------SQEQQTTKRATI 856
F_oxy_Chs1    RGVRTGVVLIWMVSNFGLAALVLSSAGLDRISPNKD------------KEAE-QLSRSNI 869
C_gra_ChsI    RGVRSVVVLAWMISNFGLAAVVLSAAGLERINPAAN------------STDD-VDGRANI 867
A_fum_ChsA    KGFRSAVVLAWVFCNFALGAVVLSAAGLDRFNSDKN------------ATD---DDRATI 880
A_nid_ChsC    KGFRSSVVLVWVFCNFALGAVVLSSAGLDRFSDDAE------------AAETDRNNRAMI 886
B_cin_ChsI    ------------------------------------------------------------ 794
```

```
S_cer_Chs1  YFNVILWLVALSALIRFIGCSIYMIVRFFKKVTFR 1131
T_M11_g683  YLSIIFWSVAGLSAFRALGSLAYLVLRLFHGE--- 911
T_def_g3959 YLSIVFWSVAGLSLFRFIGCILYLIIRLFHGE--- 922
N_cra_Chs3  YMSVVLWSVAVLSGFKFVGACWFLVVRMFRGV--- 888
F_oxy_Chs1  YMSIVLWSVAGLSAFKFIGAMWFLVVRMFRGV--- 901
C_gra_ChsI  YMSVVLWSVAGLSSFKFIGAMWFLVVRMFRGV--- 899
A_fum_ChsA  YMAVVLWSVAGLSIFKFIGAMWFLVVRMFRGV--- 912
A_nid_ChsC  YMAVVLWSVAGLSIFKFLGAMWFLVVRMFRGV--- 918
B_cin_ChsI  ----------------------------------- 794
```
