# Supplementary material for: A Novel Pathosystem With the Model Plant Arabidopsis thaliana for Defining the Molecular Basis of Taphrina Infections
Source: Environ Microbiol Rep. 2025 Jun 10;17(3):e70118. doi: 10.1111/1758-2229.70118 (PMC12152203; doi:10.1111/1758-2229.70118)
Supplement: Supplementary file 13 — FIGURE S9. Sequence conservation in class II CHS genes from Taphrina deformans. [file EMI4-17-e70118-s020.pdf]

Figure S9. Sequence conservation in class II CHS genes from *Taphrina deformans*. Multiple sequence alignment was performed with Clusal Omega. Conserved pfam domains are higlighted as follows: pink, chitin synthase 1 N-terminal (pfam08407); yellow, chitin synthase 1 (pfam01644); blue, partial chitin synthase 2 (pfam03142). Locations of the first two pfam domains are from NCBI database. The coordinates of chitin synthase 2 are from Li *et al*., 2016, because at NCBI database they were supported by alignment of only 7 sequences. Red boxes indicate conserved functional motifs listed in Li *et al*., 2016: 1, ligand binding; 2, metal ion binding site; 3, donor saccharide binding; 4, acceptor saccharide binding; 5, product binding. Blue boxes indicate conserved sequence patterns defined by Li *et al*., 2016. Blue colored amino acids in the CHS sequence from *Taphrina deformans* do not match the conserved sequence patterns.

```
S_cer_Chs2  ------------------------------------------------------------ 0
T_def_g2531 ------------------------------------------------------------ 0
A_fum_ChsB  ------------------------------------------------------------ 0
A_nid_ChsA  ------------------------------------------------------------ 0
N_cra_Chs2  -----------------MAGYGHSTAGGFGSGS--------------------------- 16
B_cin_ChsII MDRSNTPSMPPMYSETYPDEYDSIPAGHNRHGSEIRLLTSYDDPDTRPKPPPAVSVATPE 60
F_oxy_Chs2  -------------------------------------------------------MAESQ 5


S_cer_Chs2  ------------------------------------------------------------ 0
T_def_g2531 ------------------------------------------------------------ 0
A_fum_ChsB  -------------MSSNS--------------SRMYPPAPNYEEEPPAYGNYGETTSFLA 33
A_nid_ChsA  ------------------------------------------------------------ 0
N_cra_Chs2  ---------------------------------GSGPPGPQYM--LPQYD---EGDD--- 35
B_cin_ChsII HLASLKEEEQEEVIS--SCKLQQEVEISATAMSAHDPNDIAHL--LPVLP---DGPS--- 110
F_oxy_Chs2  TLLP-----KRPIIGGQTAKLQNKNRTSV----HVAFADLPRD--LPEIP---DGIS--- 48


S_cer_Chs2  ------------------------------------------------------------ 0
T_def_g2531 ------------------------------------------------------------ 0
A_fum_ChsB  DHDSSPRHQGVTMRLLPNSTDVDDDLSVDVERGASHHYGIEYS----------------- 76
A_nid_ChsA  ---------------------------MDCQNG--------------------------- 6
N_cra_Chs2  PDADA-TPAGQGVRLLTNLDNSSYISVSEITSQSSHRDNIRPSRLRQAYEPSID------ 88
B_cin_ChsII RRRSLRHPVSSP---LSTL------KSQLIGKKSSLLKNISTSSDRRSYQASVMSTDSYD 161
F_oxy_Chs2  DRRRVHKEQQH------------------LGLD--------------------------- 63


S_cer_Chs2  -MTRNPFMVE-PSNGSPNRRGASNLSKFYANANSNSR-WANPSEESLEDSYDQSNVFQGL 57
T_def_g2531 ------------------------------------------------------------ 0
A_fum_ChsB  DD---------ESTRANVQ-----YVPYSGNANGGYNR--FYGYNAEESPSRPASSLGNV 120
A_nid_ChsA  --------------RRANR-----TVRFARTAESRYPERYSYEYDPEETLSRAAPSMRNA 47
N_cra_Chs2  ARTYEPS---------------LDTRTYEPSISDR-RHMYEP-------SIDERSSYMDP 125
B_cin_ChsII DHRRAPSINTYDDHRRPPSMNTYDDHRRPPSINTYDDHRRPPTATTYDDHRRAPSIIDNV 221
F_oxy_Chs2  ---TTPPVP----PRPLSRLRDVNSHDKLPSIRSPRNLNYQP----SVRSSRSGSIFDDA 112


S_cer_Chs2  PASPSRAA--------------------LRYSPDRRHRT-------------QFYRDSAH 84
T_def_g2531 ------------------------------------------------------------ 0
A_fum_ChsB  PSIPPPAVSA---VEVPQ-YSSRPASPLRPWSPARAADWTR--PPAPPSVTGSQYERADL 174
A_nid_ChsA  PTIPPPTASG---ADEMRYTASRPASPARPWSPTRAADWVR--PPS---AAASYYERADI 99
N_cra_Chs2  PRIPPPDGGSYVSSYMG--TESMVSGHGRPWSPESATG--------YRVPPQGRYEPSEI 175
B_cin_ChsII PDLPPPES-AYRPYSPLQYSPSGRASPTRTWSPIREER--NSSEFNVPPPMGYHYEPSDL 278
F_oxy_Chs2  PSMAPPGG-SYVSYG-----MHDDGSPQRPWTPSSRVSGFTRSDLSRPPPSDGMYEPSDL 166
```

```
S_cer_Chs2  NSPVAPNRYAANLQESPKRAGEAV-IHL-----SEGSNLYPRDNADLPVDPYHLSPQQQ- 137
T_def_g2531 ----------------------------------------------------MPSMGE 6
A_fum_ChsB  NGSPRPGTPSSRYGGSPRRPLPPAPLFAGPGAAT-------------GQDTSIDIGDGN- 220
A_nid_ChsA  NGSPRPGTPSSRYGGSPRRPLPPAPLFSKPGTTT--------------QDTKIDIGDG-- 143
N_cra_Chs2  DGHARPGTPGSSYGNA-RRPLPSAPAPLHYNSPSRAASHYPRYHGGYADDVTVSMGPDD- 233
B_cin_ChsII NGSPRPGTPSTAYGGSPRRPLPPAPLFAAHGA-R----------SPFGDDATIHIPLEHE 327
F_oxy_Chs2  NGSPRPGTPSSRYGGSPRRPLPPAPLFSNSRQPV----------PPIADDATISIPLHDT 216

S_cer_Chs2  ----PSNNLFGSGRLYSQ------SSKYTMSTTSTTAPSLAEADDEK----EKYLTS-TT 182
T_def_g2531 KTNWVDESPYEMP-------------YKGFDVK---AYSVLSDDDDDGGG-----DDDND 45
A_fum_ChsB  ----DPEDPFGGGGRTN-NRHEHRGSIRSFMSD-----STMITDEKE------EMA--KI 262
A_nid_ChsA  -----EEDPFGGGGRTISSRHGPQGSVQSFTSE-----STFIADETD-------LE--KV 184
N_cra_Chs2  ----DRTDIFGPETDLSETRHLNDA--YGFRSS-----QITLSEDPHGTHARSRYDDEDD 282
B_cin_ChsII DDEMLDEDVFGPEKRDM----------------------------------RGTMRSR-- 351
F_oxy_Chs2  ----YDDDVFAPESDLSDARPHP------------------------VDRSSYMSSE-- 245
                   . :

S_cer_Chs2  SYDDQSTIFSADTFNETKFELNH--PTRQQYVRRANSESKRRMVSDLPPPSKKKALLKLD 240
T_def_g2531 DSDRQSLAPTISSKEEGAKTNYGPAPAEPQPRRRHK-----SKT--TEVVNLTEGNLIIE 98
A_fum_ChsB  NLN------EDDVVDVDPNMHYGPAPEK--QSRRGVREA--QMS--KKEVQLINGELILE 310
A_nid_ChsA  DLDEYEEESNETKSMVDPNLHYGPAPEK--QSRRGVRNA--QMA--KKEVQLVNGELILE 238
N_cra_Chs2  VSTTYSSNTGTSASGVDKFEHYGPIPEEGKHERRGVRPP--QMS--RKEVQLINGELVLE 338
B_cin_ChsII --QSYS-TFADDMESAKDYEHYGPAPSGKQERRGANRTT--QMK--KREVKLINGELILE 404
F_oxy_Chs2  --SQDT-LNEGDMEDYDKVEHYGPAPTGAQERRGV-RAP--QMS--RKEVQLINGELVLE 297
                                     *      *                 .  :. * ::

                                                                     1.
S_cer_Chs2  NPIPKGLLDTLPRRNSPEFTEMRYTACTVEPDDFLREGYTLRFA--EMNREQQIAICITM 298
T_def_g2531 REVPEKLRNLLIRHDLKEFNQVKYSAVTCDPDDFVNEGFTLRQHA--QGRETEIMVVMTM 156
A_fum_ChsB  CKIPTILHSFLPRRDDREFTHMRYTAVTCDPDDFTQRGYKLRQQIGSTMRETELFICVTM 370
A_nid_ChsA  CKIPTILHSFLPRRDDREFTHMRYTAVTCDPDDFTQRGYKLRQQIGRTMRETELFICITM 298
N_cra_Chs2  CKIPTILYSFLPRRDEVEFTHMRYTAVTCDPDDFVARGYKLRQNIGRTARETELFICVTM 398
B_cin_ChsII CKIPTILYSFLPRRDEIEFTHMRYTAVTCDPDDFVAKGYKLRQNMGVTARETELFICITM 464
F_oxy_Chs2  CKIPTILYSFLPRRGEVEFTHMRYTAVTCDPDDFVERGYTLRQTFGKTVRETELFICVTM 357
              :*  * . * *:.  **..::*:* * :****  .*:.**       ** :: : :**

S_cer_Chs2  YNEDKYSLARTIHSIMKNVAHLCKREKSHVWGPNGWKKVSVILISDGRAKVNQGSLDYLA 358
T_def_g2531 YNENEKLFTRTLHGVIKNIALLTNRTRSRTWGVDAWQKVVVLIVADGRKNINPRVLDVLT 216
A_fum_ChsB  YNEDETHFTRTMHGIMRNISHFCSRSKSRTWGKDGWKKIVVCIIADGRKKVHPRTLNALA 430
A_nid_ChsA  YNEDETHFTRTMHGVMQNISHFCSRSKSRTWGKDGWKKIVVCIISDGRKKVHPRTLNALA 358
N_cra_Chs2  YNEDEFGFTRTMHAVMKNISHFCSRNKSRTWGADGWQKIVVCVVSDGREIIHPRTLDALA 458
B_cin_ChsII YNETEIDFTRTMHAVMKNISHFCSRSKSRTWGENGWQKIVVAIISDGRQKIHPRTLDALA 524
F_oxy_Chs2  YNEDEIGFTRTMHAVMKNISHFCSRSRSRTWGETGWQKIVVCIVSDGREKIHPRTLDALA 417
            *** :  ::**:*.:::*:: : .* :*:.**  .*:*: * :::***  ::   *: *:

S_cer_Chs2  ALGVYQEDMAKASVNGDPVKAHIFELTTQVSINADLDYV--SKDIVPVQLVFCLKEENKK 416
T_def_g2531 ALGVYQDGLAQTRVNGNPVTAHLYEYTSQYSMTPDLQFRGAEKNIPPVQIAFLLKEQNQK 276
A_fum_ChsB  AMGVYQEGIAKNIVNQKQVTAHVYEYTTQVSLDSDLKFKGAEKGIMPCQVIFCLKEHNQK 490
A_nid_ChsA  ALGVYQEGIAKNVVNQKQVNAHVYEYTTQVSLDSDLKFKGAEKGIVPCQVIFCLKEHNQK 418
N_cra_Chs2  AMGVYQHGIAKNFVNQKAVQAHVYEYTTQVSLDSDLKFKGAEKGIVPCQMIFCLKEKNQK 518
B_cin_ChsII AMGVYQDGIAKNLVNGREVQAHVYEYTTQVSLDSDLKFKGAEKGIVPCQMLFCLKEKNAK 584
F_oxy_Chs2  AMGVYQHGIAKNFVNNRAVQAHVYEYTTQVSLDSDLKFKGAEKGIVPCQMIFCLKEKNQR 477
            *:****..:*:  **   * **::* *:* *:  **.:   .*.* * *: * ***.* :
```

```
                                            2.
S_cer_Chs2    KINSHRWLFNAFCPVLQPTVVTLVDVGIRLNNTAIYRLWKVFDMDSNVAGAAGQIKTMKG 476
T_def_g2531   KLNSHRWALQGLAPLLEPNICVLLDVGTRPGPDSIYKLWKAFDMDSRVAGAAGEIRILAG 336
A_fum_ChsB    KLNSHRWFFNAFGRALQPNICILLDVGTKPEPTALYHLWKAFDQDSNVAGAAGEIKAGKG 550
A_nid_ChsA    KLNSHRWFFNAFGRALQPNICILLDVGTRPEPTALYHLWKAFDQDSNVAGAAGEIKASKG 478
N_cra_Chs2    KLNSHRWFFNAFGKALNPNVCILLDVGTRPGGTSLYHLWKAFDTDSNVAGACGEIKAMKG 578
B_cin_ChsII   KLNSHRWFFNAFGRALTPNICIMLDVGTKPGGNSLYHLWKAFDTDSNVAGACGEIKAMKG 644
F_oxy_Chs2    KLNSHRWFFNAFGKALNPNVCILLDVGTRPSGTSLYHLWKAFDTDSNVAGACGEIKAMKG 537
              *:***** ::.:  * *.:  ::*****:    ::*:***.** **.****.*:*:    *

                                                  3.
S_cer_Chs2    KWGLKLFNPLVASQNFEYKISNILDKPLESVFGYISVLPGALSAYRYRALKNHEDGTGPL 536
T_def_g2531   RAWSALLNPLVASQNFEYKMSNLLDKPCESVFGYIGVLPGAFSAYRYRALAGDALGTGPL 396
A_fum_ChsB    KNMMGLLNPLVASQNFEYKMSNILDKPLESVFGYITVLPGALSAYRFFALQNDADGNGPL 610
A_nid_ChsA    KNMLGLLNPLVASQNFEYKMSNILDKPLESVFGYITVLPGALSAYRFFALQNDAEGNGPL 538
N_cra_Chs2    RFGGNLLNPLVASQNFEYKMSNILDKPLESVFGYITVLPGALSAYRYHALQNDETGHGPL 638
B_cin_ChsII   KGWMGLLNPLVASQNFEYKMSNILDKPIESVFGYITVLPGALSAYRYHALQNDHTGHGPL 704
F_oxy_Chs2    RLGANLLNPLVASQNFEYKMSNILDKPLESVFGYITVLPGALSAYRYHALQNDETGHGPL 597
              :    *:************:**:****  *******  *****:****: **  ..  * ***

                                4.
S_cer_Chs2    RSYFLGETQEGRDHDVFTANMYLAEDRILCWELVAKRDAKWVLKYVKEATGETDVPEDVS 596
T_def_g2531   ESYFRGEKID-LEADVFQQNMYLAEDRILAFELIAKRNCNWLLKYVADAYGETDCPNTVA 455
A_fum_ChsB    NQYFKGETLHGKDADVFTANMYLAEDRILCWELVAKREERWVLKFVKSAVGETDVPDTIP 670
A_nid_ChsA    NQYFKGETLHGKDADVFTANMYLAEDRILCWELVAKREERWVLRFVKSAVGETDVPDSIP 598
N_cra_Chs2    SQYFKGETLHGQHADVFTANMYLAEDRILCWELVAKRGERWVLKYVKGCTGETDVPDTVP 698
B_cin_ChsII   SQYFKGETLHGQNADVFTANMYLAEDRILCWELVAKRDERWVLKYVKGCTGETDVPDTVP 764
F_oxy_Chs2    SQYFKGETLHGQHADVFTANMYLAEDRILCWELVAKRGERWVLKYVKGCTGETDVPDTVP 657
               .** **. .  . ***  **********.:**:***  .*:*::*  . **** *: :

                  5.
S_cer_Chs2    EFISQRRRWLNGAMFAAIYAQLHFYQIWKTKHSVVRKFFLHVEFLYQFIQMLFSWFSIAN 656
T_def_g2531   EIIGQRRRWLNGATFASTYALWNFRRIWSTSHTTRRKLALHVEFLYQAVQLLFTFFGLAN 515
A_fum_ChsB    EFISQRRRWLNGAFFAAVYSLVNVKQLWKTDHSVPRKILLQIEAFYQFLNLLFTYFGLAN 730
A_nid_ChsA    EFISQRRRWLNGAFFAAVYSIVNVKQLWKTDHSLARKILLQIESVYQLLQLIFTYFGLAN 658
N_cra_Chs2    EFVSQRRRWLNGAFFAAVYSLVHFRQIWKTDHTFMRKALLHVEFLYHLLQLLFTYFSLAN 758
B_cin_ChsII   EFVSQRRRWLNGAFFAAVYSLAHFKQVWHTDHTLGRKILLHIEFIYQFIQLLFTYFSLAN 824
F_oxy_Chs2    EFISQRRRWLNGAFFAAVYSLVHFKQIWFTDHTLARKILLHMEFLYQFIQLMFTFFSLAN 717
              *::.********* **: *:   :. ::* *.*:   **   *::*  .*: ::::*::*.:**

S_cer_Chs2    FVLTFYYLAGSMNL--------VIKHGEALFIFFKYLIFCDLASLFIISMGNRPQGAKHL 708
T_def_g2531   FYLTFFFIAQS--------FGQKFHWASYVFVFLRYLCIMVTAAQFILALGNRPRGAKSL 567
A_fum_ChsB    FYLAFFFIAGSLSDERIDPFGH--NMGKYIFFVLRYACILVMCLQFIISMGNRPQGAKKL 788
A_nid_ChsA    FYLAFFFIAGSLTDEKIDPFGH--NMGKYIFIVLRYACVLVMCLQFIFSMGNRPQGAKKL 716
N_cra_Chs2    FYLAFYFIAGGLADPHVDPFNSDGHVARIIFNILRYVCVLLICTQFILSLGNRPQGAKRM 818
B_cin_ChsII   FYLTFYFIAGSLSVDNMDPFGH--SIGKYIFYVLKYVCVLLTATQFILSMGNRPQGARKL 882
F_oxy_Chs2    FYLTFYFVAGGLTDPKVDPFGH--NIATVIFHILRYACVLLISTQFILSLGNRPQGSKKL 775
              * *:*:::* .                .  :*  .::*   .    .   **:::****:*::  :

S_cer_Chs2    FITSMVILSICATYSLICGFVFAFKSLASGTE---------SHKIFVDIVISLLSTYGLY 759
T_def_g2531   FLLCLILYSVIMIYTLGCASYLGVSSILEGLK---EGGSILANKQFANIVLSIVATTGIY 624
A_fum_ChsB    YMSGIIVYSIIMVYTAFCALYLVVLELMAKAG-VGKKELAVSDSLFINIVVSLLSTVGLY 847
A_nid_ChsA    YLSSMIVYSIVMAYTAFCTLYLIVLELMAKTG-HD-VPITMSDTLFVNIVVSLLSTVGLY 774
N_cra_Chs2    YLASMIIYAVIMVYTTFATIFIVVRQIQPSQKSDDKPDLELGNNVFTNLIVSVASTLGLY 878
B_cin_ChsII   YYMSMIIYSIIMVYTLFSTIYIVVREVHD-----NAKNLVMGNNLFTNLVVSLCSTLGLY 937
F_oxy_Chs2    YLISMIIYSIIMVYTTFATFYIIIHQLTS-----KDDKIEMGDNVFTNMIVSILSTIGMY 830
              :   ::: ::    *:   .    :    .:                 ... *  ::::*:  :*  *:*
```

```
S_cer_Chs2   FFSSLMYLDPWHMFTSSIQYFLTLPAFTCTLQIFAFCNTHDVSWGTKGSTQESKQLSKAI 819
T_def_g2531  FVASLMYLDIWHMFTSFLPYMLLVPFYICTLTIFAFCNIHDLSWGTKEEDVADLDVVEAK 684
A_fum_ChsB   FYSSFLYLDPWHMFTSSAQYFALLPSYICTLQVYAFCNTHDVTWGTKGDNTINNDLGAAR 907
A_nid_ChsA   FFTSFMYLDPWHMFTSSAQYFALLPSYICTLQCYAFCNTHDVTWGTKGDNTINTDLGTAR 834
N_cra_Chs2   FVMSFLYLDPWHMFTSAIQYFVLLPSYICTLQIYAFCNTHDVTWGTKGDNVMRTDLGGA- 937
B_cin_ChsII  FLMSFLYLDPWHMFTSSGAYFALLPSYICTLQVYAFCNTHDVTWGTKGDNVMSTDLGAA- 996
F_oxy_Chs2   FIMSILYLDPWHMITSSAQYFILLPSYICTLQVYAFCNTHDVTWGTKGDNVMKTDSWGA- 889
             *  *::*** ***:**   *:  :* : ***  :**** **::**** .     :   *

S_cer_Chs2   VVQGPDGKQIVET----DWPQEVDKKFLEIKSRLKEPEFEESSGNEKQSKNDYYRDIRTR 875
T_def_g2531  VLKDDNTKVKIELYQGADAEGSYEDALANLRLRKPVA---EREQDRSRIQEDYFK----- 736
A_fum_ChsB   IINGTTVEVEMPS-EQLDIDSGYDAALRNLRDRLEVP---PPPVSENQQQEDYYRAVRTY 963
A_nid_ChsA   IINGSIVEVEMPS-EQLDIDSGYDAALRNLRDRLEVP---DPGVSESQQQEDYYRAVRTY 890
N_cra_Chs2   IGKGSTVELEMPS-DQLDIDSGYDECLRNLRDRVMVP---AVPVSEDQLQQDYYKSVRTY 993
B_cin_ChsII  SGKGQTVELEMPS-EQLDIDSGYDEALRNLRDRLEVP---SPPISESQQQEDYYKSVRTY 052
F_oxy_Chs2   VGKGETVELEMPS-EQLDIDSGYDEALRNLRDRLEVP---ESPPSESQLQEDYYKSVRTY 945
               :.   :  :      *     :  : ::: *           ...: ::**::

S_cer_Chs2   IVMIWMLSNLILIMSIIQVFTPQD-TDNGYLIFILWSVAALAAFRVVGSMAFLFMKYLRI 934
T_def_g2531  ------------------------------------------------------------ 736
A_fum_ChsB   MVSIWMVANVILAMSVSEIYGVDSGGTNVYLGIILWSVAVLALIRAVGSTTYAILLVVQK 023
A_nid_ChsA   MVSVWMVANVVLAMAVSEVYGVGSSGTNVYLAIILWSVAVLAIIRAIGSTAYAVLYLIQK 950
N_cra_Chs2   MVVSWMVANATLAMAVSEAYGDSEIGDNFYLRFILWAVAALALFRALGSTTFAAINLVSA 053
B_cin_ChsII  MVLVWMIANGILAMAVSEAYGPDHLGTNYYLTFLLWAVAALALFRAVGSSAFGIINCVEA 112
F_oxy_Chs2   LVLTWMIGNGILGMAVSEIYSARGIGDNYYLRFLLWSVAALAVFRAIGSTTFAVLNVINM 005


S_cer_Chs2   IVSYRNKVEGS---GSWEVSK---LD--LPNVFHKKG----------------------- 963
T_def_g2531  ------------------------------------------------------------ 736
A_fum_ChsB   IVEGKTKFDAGNIVNSNAATSS-YVSSRSTAQYG-GGTSFKDKVTEAGWTLKRTAGKAMF 081
A_nid_ChsA   LVEGKAKFQAGNIASANASAAGSSLGTKSNVSYGSKGLNMTDRINETKWAISRGMQKAMF 010
N_cra_Chs2   LVEGRVRLRLNMKGFRWIKE---KWGDADVKGKF-------EGLGDRARGLARR------ 097
B_cin_ChsII  IVEGRIRVSMT--VPRWMGG----WSSKISEGIS-------EGISSVGNAVKRN------ 153
F_oxy_Chs2   IVEGRVRLSLK--APRWMGG----LKERVNDKMS-------SVSSNLRS----------- 041


S_cer_Chs2   --- 963
T_def_g2531  --- 736
A_fum_ChsB   WKK 1084
A_nid_ChsA   WKK 1013
N_cra_Chs2   --- 1097
B_cin_ChsII  --- 1153
F_oxy_Chs2   --- 1041
```
