# Supplementary material for: A Novel Pathosystem With the Model Plant Arabidopsis thaliana for Defining the Molecular Basis of Taphrina Infections
Source: Environ Microbiol Rep. 2025 Jun 10;17(3):e70118. doi: 10.1111/1758-2229.70118 (PMC12152203; doi:10.1111/1758-2229.70118)
Supplement: Supplementary file 14 — FIGURE S10. Sequence conservation in class III CHS genes from Taphrina species. [file EMI4-17-e70118-s018.pdf]

Figure S10. Sequence conservation in class III CHS genes from *Taphrina* species. Multiple sequence alignment was performed with Clusal Omega. Conserved pfam domains are higlighted as follows: pink, chitin synthase 1 N-terminal (pfam08407); yellow, chitin synthase 1 (pfam01644); blue, partial chitin synthase 2 (pfam03142). Locations of the first two pfam domains are from NCBI database. The coordinates of chitin synthase 2 are from Li *et al*., 2016, because at NCBI database they were supported by alignment of only 7 sequences. Red boxes indicate conserved functional motifs listed in Li *et al*., 2016: 1, ligand binding; 2, metal ion binding site; 3, donor saccharide binding; 4, acceptor saccharide binding; 5, product binding. Blue boxes indicate conserved sequence patterns defined by Li *et al*., 2016. Blue colored amino acids in the CHS sequence from *Taphrina* species do not match the conserved sequence patterns.

```
T_M11_g677    MAYNSRYTR---------------------------------------------GYAQDE 15
T_def_g3955   MAYNSRYSK---------------------------------------------GYTQDE 15
B_cin_ChsIIIb ----------------------METSRS----W----------------NSSAENYPMNE 18
F_oxy_Chs3    MGFN---PQGQGNGPNYDAPREMQDLPAGQAYHFRESDETAAARVSPVSNPYEPDYDQLS 57
A_fum_ChsC    ----------MGT-PRPYSAHSPQESRS--SFYSQPSQ-----------SPTQPTYGRDD 36
N_cra_Chs1    MAYH-GR-------GDGYDGHQLQDLPGGHNQGDQ------------------------- 27
C_gra_ChsIII  MAYRGGH-------ENDYDGHNMQDLPAGTGRYHLPPQ-------------------DEG 34
B_cin_ChsIIIa MAYSGNGGY---------DEHKLQDLPPGGGNYHHPQD-------------------EEE 32
A_fum_ChsG    MAYQGSGSHSPP--HYDDNGHRLQDLPH--GSYEEEAS---------------------- 34
A_nid_ChsB    MAYHGSGPQSPGE-HTYDDGHQLRDLSHSNTSYEEEAS---------------------- 37

T_M11_g677    VPINRH-DASDEEEEDLGDVDLNDGMPMQASLNPFSDTAYSG------------------ 56
T_def_g3955   APINHY-DLSDD-EEDLGNTQFPEPESLRSGMSPFGDTAYQG------------------ 55
B_cin_ChsIIIb ----L-IQHSNNSVTDPAT-QLDNTDHISDVHHSNDAHDVG-----ESLLSDTSGYHPTT 67
F_oxy_Chs3    PPPPLGAQRPVPEQNESSRDLLHSSYQGSVGHNSFDGHSF-----------------GHN 100
A_fum_ChsC    -------------AEDQQQSLLRR-SLASPNGWSYDDPNVS--------TDSLRRYTL-- 72
N_cra_Chs1    -------------HDDAQAPFLS--ENP----MPYDND---RLGTDTPPVRPVSAYSLTE 65
C_gra_ChsIII  -------------VDDVGQSLLRDPHAP----GPYE-S---HLGPAEVPGRPVSAYSLTE 73
B_cin_ChsIIIa -------------G---ALPLLDSGGHGGPFNSPYDSHSQGGLRANTPPVRPVSAYSLTE 76
A_fum_ChsG    ------------------RGLL--SHQQGPFTGPFDDPQQHGS----STTRPVSGYSLSE 70
A_nid_ChsB    ------------------HGLL--SSQQSPFAGPFDDPHQQRGLTASPVQRPTSGYSLTE 77
                                   :

T_M11_g677    -YRPGYD--------------TELDEPIPYQQSVMHQSHYSERSLVEDLKTPIPSITRTS 101
T_def_g3955   -YQPSND--------------DEYEHPTVHDSSSRNANGLELRG-TSRLSTPVQSG---- 95
B_cin_ChsIIIb PYVTGTGQVQR-------------------------------------QRSPTSS----- 85
F_oxy_Chs3    SYGPGAFGHYPADQHGRMPGSPGYEY--PEPEYDVEASRLAESRLSVMHRAPTMQDW--G 156
A_fum_ChsC    --------HDP--------GITAFAPPYPESEA--A------------------------ 90
N_cra_Chs1    SYAPGAGTTRA--------GVAVNPTPPPHGGYGGG------------------------ 93
C_gra_ChsIII  SYAPGAGARTP--------VPGETA---FASGFS-Q------------------------ 97
B_cin_ChsIIIa TYANDPQ---P--------YSSDYN---SSHTYNEQ------------------------ 98
A_fum_ChsG    TYAPEAAYHDP--------YTQPS----PGSVYSAQ------------------------ 94
A_nid_ChsB    SYAPDAAYHDP--------YSANQ------SVYS-G------------------------ 98

T_M11_g677    SPYEPSLKERF--------AGSPELGKQPEVDPEAGFR-ERQ-------QPLRRGHTRKV 145
T_def_g3955   -FHSPALSTKYGFLEKDPHEIVTTPFSHAESDPEAGFR-ERQ-------QPLKRGHTRRV 146
B_cin_ChsIIIb ------VNGEEFFGDQHHLPDIDQDFYRS---PEDSFNAQDMPSANGARGALRRRATRKI 136
F_oxy_Chs3    QNGEALSVPDFAHGRP------DST--YQEFDVDESWM-MRQQQNQ-LAGGLGRSKTRKV 206
A_fum_ChsC    -DVR------------------SARMSGYSGIEMDAWQ-RRQGV---KPSALRRYGTRKI 127
N_cra_Chs1    -GVSSGVDQGYNYGGD---YATDPAYRMSAIDEDDSWL-RRQQPNAAPTGGLKRYATRKV 148
C_gra_ChsIII  -ELDSDANGGFGYGR-----------PASTVDADESWT-RRQQPGH-QAGGLKRYATRKI 143
B_cin_ChsIIIa --LE--DNPY---------PQTDTPLSRAGTTSTEAWR-QRQAPQA---GGLKRYATRKV 141
A_fum_ChsG    --SA--ENPAAAFGVP---GRVASPYARSDTSSTEAWR-QRQAP-GGGPGGLRRYATRKV 145
A_nid_ChsB    --HS--ENPAAAFGVP---GRVASPYARSETSSTEAWR-QRQAGAAGGGNGLRRYATRKV 150
                                                 .:              * *  **::
```

```
T_M11_g677       KLVN---GDIFCTDYPVPLPIQNAIQPKYR-DPESGSEEFTHLRYTRATCDPNDFSQKNG 201
T_def_g3955      KLVN---GNIFCTDYPVPLPIQNAIQPKYR-DPESGSEEFTHLRYTRATCDPNDFTQKNG 202
B_cin_ChsIIIb    KLIQGGQGSVLSANYPVPSAIKNALQPQYR-DIENGTSEFSEMRYTAVTCDPNDFTLMNG 195
F_oxy_Chs3       KLVQ---GSVLSIDYPVPSAIKNAVEPRYRSGPGSMEEEFTKMRYTAATCDPNDFTLRNG 263
A_fum_ChsC       NLVQ---GSVLSVDYPVPSAIQNAIQAEYRDAEEAFHEEFTHMRYTAATCDPDEFTLRNG 184
N_cra_Chs1       KLVQ---GSVLSLDYPVPSAIRNAVQPKYR-DEEGNNEEFFKMRYTAATCDPNDFTLKNG 204
C_gra_ChsIII     KLTQ---GTVLSIDYPVPSAIKNAVQPKYR-DVEGGSEEFMKMRYTAATCDPNDFTLKNG 199
B_cin_ChsIIIa    KLVQ---GSVLSVDHPVPSAIKNAIQQKYRNDLEGGSEEFTHMRYTAATCDPDEFTLKNG 198
A_fum_ChsG       KLVQ---GSVLSVDYPVPSAIQNAIQAKYRNDLEGGSEEFTHMRYTAATCDPNEFTLHNG 202
A_nid_ChsB       KLVQ---GSVLSVDYPVPSAIQNAIQAKYRNDLEGGSEEFTHMRYTAATCDPNEFTLHNG 207
                 :* :    *  :::. ::***  *:**::  .**         .** .:*** .****::*:   **

                              1.
T_M11_g677       YNLRPALYERNTELLIAVTYYNEDKVLTARTLHGIMENIKDICQDKKSKFWNKGSPAWQN 261
T_def_g3955      YNLRPALYQRNTELLIAVTYYNEDKFLTARTLHGVMQNIKDICNDKKSRFWNSTSPAWQN 262
B_cin_ChsIIIb    YTLRQHIYNRHTELLIAITYYNEDKVLFARTLHGVMKNIRDIVNLKKSRFWTQGSAAWEK 255
F_oxy_Chs3       FNLRPKMYNRHTELLIAITYYNEDKVLLARTLHGTMQNIRDIVNLKRSKFWNKGGPAWQK 323
A_fum_ChsC       YNLRPAMYNRHTELLIAITYYNEDKVLTARTLHGVMQNVRDIVNLKKSEFWNKGGPAWQK 244
N_cra_Chs1       YDLRPRMYNRHTELLIAITYYNEDKVLLSRTLHSVMTNIRDIVNLKKSSFWNRGGPAWQK 264
C_gra_ChsIII     YDLRPRMYNRHTELLIAITYYNEDKVLLSRTLHGVMQNIRDIVNLKKSTFWNKGGPAWQK 259
B_cin_ChsIIIa    YNLRPAMYNRHTELLIAVTYYNEDKQLTARTLHGVMQNIRDIVNLKKSDFWNVGGPAWQK 258
A_fum_ChsG       YNLRPAMYNRHTELLIAITYYNEDKTLTSRTLHGVMQNIRDIVNLKKSEFWNKGGPAWQK 262
A_nid_ChsB       YNLRPAMYNRHTELLIAITYYNEDKTLTARTLHGVMQNIRDIVNLKKSEFWNKGGPAWQK 267
                 : **   :*:*:*******:******* * :****. * *::** : *:* **.  . **::

T_M11_g677       IVVCLVFDGIDPCDKGTLDLLATIGIYQDQIMKGKINGKKPTAHIFEYTTQVSVNDRMQL 321
T_def_g3955      IVVCLVFDGIDPCDKGTLDLLATIGIYQDQIMKGEIDGKKPTAHIFEYTTQVSVNDNMQL 322
B_cin_ChsIIIb    IVVCLVFDGFEKADPGVLDLLTTVGVYQDGVRKQDVDGKETTAHVFEFTTQLSITPDLQL 315
F_oxy_Chs3       IVVCLVFDGIDKVDKNVFDVLATVGIYQDGVLKKDVNGKETVAHIFEYTSQVSVTPDQQL 383
A_fum_ChsC       IVVCLVFDGIEPCDKNTLDVLATIGVYQDGVMKKDVDGRETVAHIFEYTTQLSVTPTQQL 304
N_cra_Chs1       IVVCLVFDGLDKTDKNVLDVLATVGVYQDGVIKKDVDGKETVAHIFEYTSQLSVTPNQAL 324
C_gra_ChsIII     IVVCLVFDGIEKTDKSVLDVLATVGIYQDGVVKKDVDGKETVAHIFEYTSQLSVTPSQQL 319
B_cin_ChsIIIa    IVVCLVFDGIDPCDKDTLDVLATIGIYQDGVMKKDVDGKETVAHVFEYTTQLSVTASQQL 318
A_fum_ChsG       IVVCLVFDGIDPCDKDTLDVLATVGVYQDGVMKRDVDGKETVAHIFEYTTQLSVTPNQQL 322
A_nid_ChsB       IVVCLVFDGIDPCDKDTLDVLATVGIYQDGVMKRDVDGKETVAHIFEYTTQLSVTPNQQL 327
                 *********:  *  ..:*:*:*:*** : *  .::*:: .**:**:*:*:*:.    *

                                                                      2.
T_M11_g677       VRPEAN-DVD-ALPPVQFIFCLKQENEKKINSHRWLFNAFGKILQPEVCVLLDAGTKPGK 379
T_def_g3955      IRPSEN-DPD-ALPPVQFIFCLKQENEKKINSHRWLFNAFGKILQPEVCVLLDAGTKPGK 380
B_cin_ChsIIIb    SRPNSDVDDSSNLPPVQLLFCLKQKNTKKINSHRWLFNAFGRILNPEVTILIDAGTKPGP 375
F_oxy_Chs3       VRPDPD-KPHRNLPPVQFIFCLKQKNSKKINSHRWLFNAFGRILNPEVAILIDAGTKPAP 442
A_fum_ChsC       VRPQPN-DPS-NLPPVQMLFCLKQKNSKKINSHRWLFNAFSRILNPEICILLDAGTKPGS 362
N_cra_Chs1       IRPVDD-GPQ-TLPPVQFIFCLKQKNTKKINSHRWLFNAFGRILNPEVCILLDAGTKPSP 382
C_gra_ChsIII     IRPVDD-GPS-TLPPVQFIFCLKQKNSKKINSHRWLFNAFGRILNPEVCILIDAGTKPSP 377
B_cin_ChsIIIa    IRPTEG-DAN-CLPPVQMMFCLKAKNTKKINSHRWLFNAFGRLLNPEVCILLDAGTKPGP 376
A_fum_ChsG       IRPTDD-GPS-TLPPVQMMFCLKQKNSKKINSHRWLFNAFGRILNPEVCILLDAGTKPGP 380
A_nid_ChsB       IRPTDD-GPS-TLPPVQMMFCLKQKNSKKINSHRWLFNAFGRILNPEVCILLDAGTKPGP 385
                  **   .     *****::**** :* ************.::*:**: :*:*****.

T_M11_g677       RSIYHLWKAFFNDANLGGACGEIHAMLSNG-RKLVNPLVATQNFEYKMSNILDKPLESSF 438
T_def_g3955      RSIYHLWKAFFNDANLGGACGEIHAMLSKG-KKLVNPLVATQNFEYKMSNILDKPLESSF 439
B_cin_ChsIIIb    RSLLALWEAFYNDKNLGGACGEIHAMLGSRNSKLLNPLVAIQNFEYKISNVLDKPLESSF 435
F_oxy_Chs3       RALLSLWEGFYNDRDLGGACGEIHVMLGKGGKMLLNPLVAVQNFEYKISNVLDKPLESAF 502
A_fum_ChsC       KSLLALWEAFYNDKTLGGACGEIHAMLGRGWRNVLNPLVAAQNFEYKISNILDKPLESAF 422
N_cra_Chs1       RSLLALWEGFYNDKDLGGACGEIHAMLGKGGKKLLNPLVAVQNFEYKISNILDKPLESAF 442
C_gra_ChsIII     RSLLALWEGFYNDKDLGGACGEIHAMLGKGGKKLLNPLVAVQNFEYKISNILDKPLESSF 437
B_cin_ChsIIIa    KSLLSLWEGFYNDKDLGGACGEIHAMLGKGGKKLLNPLVAGQNFEYKISNILDKPLESSF 436
A_fum_ChsG       KSLLSLWEAFYNDKDLGGACGEIHAMLGKGWKNLINPLVAAQNFEYKISNILDKPLESSF 440
A_nid_ChsB       KSLLYLWEAFYNDKDLGGACGEIHAMLGKGWKKLLNPLVAAQNFEYKISNILDKPLESSF 445
                 ::: **:.*:**  *********.**.     ::***** ******:**:*******:*
                        3.                                             4.
```

```
T_M11_g677     GYVSVLPGAFSAYRYRAIQGRPLDQYFRGDHSLANKLGEKGVNGMGIFKKNMFLAEDRIL 498
T_def_g3955    GYVSVLPGAFSAYRYRAIQGRPLDQYFRGDHSLAGVLGNKGVNGMGIFRKNMFLAEDRIL 499
B_cin_ChsIIIb  GYVTVLPGAFSAYRFRAIMGRPLEQYSHGDHTLL--SGKKSINNMNIFKENMFLAEDRIL 493
F_oxy_Chs3     GYVSVLPGAFSAYRFRAIMGRPLEQYFHGDHTLSKSLGKKGIDGMNIFKKNMFLAEDRIL 562
A_fum_ChsC     GYVSVLPGAFSAYRYRAIMGRPLEQYFHGDHTLSKRLGKKGIEGMNIFKKNMFLAEDRIL 482
N_cra_Chs1     GYVSVLPGAFSAYRFRAIMGRPLEQYFHGDHTLSKLLGKKGIEGMNIFKKNMFLAEDRIL 502
C_gra_ChsIII   GYVSVLPGAFSAYRFRAIMGRPLEQYFHGDHTLSKILGKKGIDGMNIFKKNMFLAEDRIL 497
B_cin_ChsIIIa  GYVSVLPGAFSAYRFRAIMGRPLEQYFHGDHTLSKILGKKGIEGMNIFKKNMFLAEDRIL 496
A_fum_ChsG     GYVSVLPGAFSAYRFRAIMGRPLEQYFHGDHTLSKQLGKKGIEGMNIFKKNMFLAEDRIL 500
A_nid_ChsB     GYVSVLPGAFSAYRFRAIMGRPLEQYFHGDHTLSKQLGKKGIEGMNIFKKNMFLAEDRIL 505
               ***:**********:***  ****:**  :***:*      *:*.::.*.**:**********

                                                     5.
T_M11_g677     CFELVAKAGARWKLTYVKASQAETDVPEQVAEFISQRRRWLNGSFAASIYSMVHFPRLYR 558
T_def_g3955    CFELVAKAGAKWKLTYVKASQAETDVPEQAAEFISQRRRWLNGSFAASIYSMVHFPRLYR 559
B_cin_ChsIIIb  CFELVVKEGQNWHLSYVKAAKGETDVPEGPAEFLSQRRRWLNGSFAASLYSLIHFGRMYK 553
F_oxy_Chs3     CFELVAKASQKWHLSYIKASKGETDVPEGAAEFIGQRRRWLNGSFAMSLYSLMHFGRMYG 622
A_fum_ChsC     CFELVAKAGYKWHLTYVKASKGETDVPEAAPEYISQRRRWLNGSFAASLYSIMHFGRIYK 542
N_cra_Chs1     CFELVAKAGQKWHLSYIKAAKGETDVPEGAPEFISQRRRWLNGSFAASLYSLMHFGRMYK 562
C_gra_ChsIII   CFELVAKAGQKWHLSYIKAAKGETDVPEGAAEFISQRRRWLNGSFAASLYSLMHFGRMYK 557
B_cin_ChsIIIa  CFELVAKAGSKWHLTYIKAAKGETDVPEGAAEFIGQRRRWLNGSFAAGMYSIMHFGRMYK 556
A_fum_ChsG     CFELVAKAGSKWHLTYVKASKAETDVPEGAPEFISQRRRWLNGSFAAGIYSLMHFGRMYK 560
A_nid_ChsB     CFELVAKAGSKWHLSYVKASKGETDVPEGAPEFISQRRRWLNGSFAAGIYSLMHFGRMYK 565
               *****.*  .  .*:*:*:**::.******    *::.***********.:**::**  *:*

T_M11_g677     SNHSIARMVIFHLQLLYNIFSTIFSWFALANLWLTFSVVIQLTSQQSPFLAPSSCNLCHA 618
T_def_g3955    SNHSFFRMFVFHVQLVYNIFSTIFSWFALANLWLTFSVVIQLTSQQSPFLGTNSCNTCHA 619
B_cin_ChsIIIb  SGHSLLRMIMFHFQLLYNIANVIFSWFSLSSYWLTTTVIMDLVGTPVTASNY-------- 605
F_oxy_Chs3     SGHNLIRLFFLHIQFVYNLVNVLFSWFSLAAFYLTTTIIMKLVGTPQVLSEY-------- 674
A_fum_ChsC     SGHSFVRMFFLHIQMIYNCCQLIMTWFSLASYWLTSSVIMDLVGTPSSHNKY-------- 594
N_cra_Chs1     SGHNIVRMFFFHVQLIYNIANVIFTWFSLASYWLTTTVIMDLVGTPVTASSS-------- 614
C_gra_ChsIII   SGHNLVRMIFFHIQLVYNILQVLFTWFSLGSYYLTTTVIMDLVGNPVVSEDP-------- 609
B_cin_ChsIIIa  SGHNIVRMFFLHIQFLYNVFSNFLSWFMLSSFWLTTTVIMDLVGTPTAASDT-------- 608
A_fum_ChsG     SGHNIVRMFFLHIQMLYNIFSTVLTWFSLASYWLTTTVIMDLVGTPSDNNGN-------- 612
A_nid_ChsB     SGHNIVRMFFLHLQMLYNWFSTFLTWFSLASYWLTTSVIMDLVGTPSSSNGY-------- 617
               *.*.: *:..:*.*::**  .  .::** *.  :** ::::.*..

T_M11_g677     IQTGTLSGAVTFSQVNATVTGLGTSASGV-LLTQYLFNGHNYTSAELIGQSTRCVCGTDV 677
T_def_g3955    IQSGITSGTLLFNSTAGLITGLSLDGSGTTTLAALDFNKHAYTAAELVGKSTRCVCATDV 679
B_cin_ChsIIIb  --------------------------------------HGWP---------FGDTASPI 617
F_oxy_Chs3     --------------------------------------HGWP---------FGDTATPI 686
A_fum_ChsC     --------------------------------------KAWP---------FGNDASPI 606
N_cra_Chs1     ----------------------------------SAEHHGWP---------FGDTVTPF 630
C_gra_ChsIII   ----------------------------------KLARHGWP---------FGDTATHI 625
B_cin_ChsIIIa  ----------------------------------TALHHGWP---------FGDTATPI 624
A_fum_ChsG     --------------------------------------KAFP---------FGKTATPI 624
A_nid_ChsB     --------------------------------------TAFP---------FGKTATPI 629
                                                        :              : .

T_M11_g677     FNLVLQYVYLGALLLSFLLALGQRPKGSIKTYTGAMAVFAVCQVYLLACSFYLAIKALIS 737
T_def_g3955    FNLVLQYVYLGTILLSFLLALGQRPKGSVKTYLGAMTLFAFCQIYLLACSFYLAITALMT 739
B_cin_ChsIIIb  FNHVIEYIYLAFVITQFILALGNRPKGSQVTYLVLFVVFGFIQLYIIILSFYLVYRALRT 677
F_oxy_Chs3     VNVLIKYIYIAFLVLQFVLALGNRPKGAQYTYVLSFMVFGLIQLYLLVLTGYLVYRAFTG 746
A_fum_ChsC     VNFFVKYGYLLVLMLQFVLALGNRPKGTKLAYTMSFLWFSLVQFYVLILSFYLVANAFMG 666
N_cra_Chs1     FNAVLKYIYLAFVILQFILALGNRPKGSKWTYITSFFVFSLIQSYILVLSGYLVARAFSV 690
C_gra_ChsIII   FNALLKYLYLAFVILQFILALGNRPKGSKYTYIASFVVFGLIQSYILILSMYLVVQAFQT 685
B_cin_ChsIIIa  INTILKYLYLGFLLVQFILALGNRPKGSKATYIVSFVVFGVINLYVLVLSMYLVVRAFSS 684
A_fum_ChsG     INTIVKYVYLGFLLLQFILALGNRPKGSKFSYLASFVVFGIIQVYVVIDALYLVVRAFSG 684
A_nid_ChsB     INTLVKYIYLAFLLLQFILALGNRPKGSKLSYLASFVAFGIIQLYVVVDALYLVVRAFTG 689
               .* .::* *:  ::  .*:****:****:  :*    :  *.. : *::  : **.  *:
```

```
T_M11_g677       VQNL---------QDFLEGFFTATADNPSSGNGVIVVALASTFGLYFFASFIYMDAWHML 788
T_def_g3955      VSSF---------KDFMNGFFVASANNTSSGNGVIVVALASTFGLYFVASFLYMDAWHMF 790
B_cin_ChsIIIb    PIGE-QIDTSSA-AAFFESMFGG---T--GVAGVILLALVTIYGLNYVASFLYLDPWHMF 730
F_oxy_Chs3       TPIEEQISFESG-KAFFDSFFGG---D-TGVAGLIIIALFTIYGLNYIASFLYLDPWHMF 801
A_fum_ChsC       GM-I-DFDFDQGVGNFLSSFFS------STGGGIVLIALVSTYGIYIVASILYMDPWHIL 718
N_cra_Chs1       PLDQ-QLQLDNA-KDAMASLFGG---S--GSAGVILVALVTIYGLYFLASFMYLDPWHMF 743
C_gra_ChsIII     PLSQ-QISLDSG-KDFVQSFFGG---T--NAAGVILVALVTIYGLNFIASFMYLDPWHMF 738
B_cin_ChsIIIa    GDSL-DTSSAK---DFFESFFGD---SSSSGAGIIILALAATFGLYFVASFMYMDPWHMF 737
A_fum_ChsG       SAPM-DFTTDQGVGEFLKSFFS------SSGAGIIIIALAATFGLYFVASFMYLDPWHMF 737
A_nid_ChsB       GAPM-DFNTDDGIGAFLSSFFG------SSGAGIIIIALAATFGLYFVASFMYLDPWHMF 742
                                 . .:*           *::::** : :*:  .**::*:* **::

T_M11_g677       HSFPQYLLLAPSYTNILNVYSFCNTHDVSWGTKGADKPEALPAVEAKKGGHA-SAIVEEN 847
T_def_g3955      HSLPQYILLAPSYVNILNVYSFCNTHDVSWGTKGSDKPEALPALEAKKGD-R-SAIVEEN 848
B_cin_ChsIIIb    HSFPQYIILASTYINILMVYAFNNWHDVSWGTKGSDESEKLPSANVIKDSKSGVEMVEEE 790
F_oxy_Chs3       HSFPQYLVLMSTYINILMVYAFNNWHDVSWGTKGSDTAEALPSAMIVKDEKGKEAVVEEI 861
A_fum_ChsC       TSSWAYFLGMTTSINILMVYAFCNWHDVSWGTKGSDKADALPSAQTKKADGSKSNFIEEI 778
N_cra_Chs1       HSFPYYMLLMSTYINILMIYAFNNWHDVSWGTKGSDKAEALPSANVSKGEKD-EAVVEEI 802
C_gra_ChsIII     TSFPHYLVLMSTYINILMVYAFNNWHDVSWGTKGSDKTEALPSAQVSKGEKD-EAVVEEI 797
B_cin_ChsIIIa    HSFGPYLLLMSSYINILMVYAFSNWHDVSWGTKGSDKAEALPSAKTTKVDGK-AAVIEEI 796
A_fum_ChsG       TSFPAYMCVQSSYINILNVYAFSNWHDVSWGTKGSDKADALPSAKTTKDEGK-EVVIEEI 796
A_nid_ChsB       TSFPAYMAVQSSYINILNVYAFSNWHDVSWGTKGSDKADALPSAKTTGGKGE-EAVIEEI 801
                  *   *:    :  *** :*:* * *********:*  : **:            .:**

T_M11_g677       EQEQSEIDLAFESTVKRALVPLPKA--TSQPSDSPEDGYKAFRTRLIIAWIFSNIIIILG 905
T_def_g3955      EQEQAEIDSAFETTVKRALAPMPKQ--EKKEAPSLEDGYKTFRTSLIIAWIFSNLLIVLA 906
B_cin_ChsIIIb    EMQQTDIDQKFQETVLRTLAPVEVE--VTVETKEVDDTYKSFRTRLVVCWILSNMLLVGI 848
F_oxy_Chs3       EQEQEDIDSKFEKVVWRALAPMSEMAEEKPEKKDVEDSYKSFRTGLVILWLLCNIVLIVV 921
A_fum_ChsC       DKPQADIDSQFEATVKRALAPYQEP--KEDSTISLDDSYRNFRTSLVLLWILSNLLVSLL 836
N_cra_Chs1       EKPQEDIDQQFEATVRRALAPYKED--ETPEPKDLEDSYKSFRTMLVVSWLFSNCLLAVV 860
C_gra_ChsIII     DMPQEDIDSQFESTVKRALEPFKEV--EEVEKPDIEDSYKSFRTGLVVSWLFSNTFSIIV 855
B_cin_ChsIIIa    DRPQEDIDSQFEATVKRALSPFVPE--VEDESKTLEDSYKSFRTKLLISWVFSNALLAVA 854
A_fum_ChsG       DKPQADIDSQFEATVKRALTPYVPP--VEKEEKTLEDSYKSFRTRLVTFWIFSNAFLAVC 854
A_nid_ChsB       DKPQADIDSQFEATVKRALTPYVPP--EEKEEKSLDDSYKSFRTRLVTLWLFSNGLLAVC 859
                 :  * :**  *: .* *:* *              :* *: *** *:  *::.* .

T_M11_g677       FTSTDLQKDFGLVTTTKTRTASYFQFILWSTAALSTIRCLGCVFFLCKTGILRIPGLSKR 965
T_def_g3955      FTSSDIQTAFGIGQSTKTRTATYFQFILWSTAVLSFIRFVGCLYFVTQTGIQQIPGFSKR 966
B_cin_ChsIIIb    VTSDDF-AFLGVGPLR-PGPPYYFKFLLYATAFLSIIRFLGFLWFLGRTNIMYCF--AKR 904
F_oxy_Chs3       VTTDDF-ITLGVSKAADVRTPTYFRVLLYSTAVLSIVRFFGFLWFIGRTGIMCCI--ARR 978
A_fum_ChsC       ITNDGI-RKMCLTNTSTTRTQYYFQVILWATAGLSIFRFIGSIYFLGKSGILCCV--TRR 893
N_cra_Chs1       ITSDNF-NTFGIGQTASARTAWFFKFLLFATGALSVIRFIGFCWFLGRTGIMCCF--ARR 917
C_gra_ChsIII     ITSDNF-DSFGIGESSSKRTASYFSFLLYSTAILSLVRFFGFLWFLGRTGIMCCF--ARR 912
B_cin_ChsIIIa    ITSDSV-DGFGFGNTASIRTAKFFEILLYSTAFLALIRFLGCSYFLFKSGIMCCF--MRR 911
A_fum_ChsG       ITSDGV-DKFGFTNSATDRTQRFFQALLWSNAVVALFRFIGACWFLGKTGLMCCF--ARR 911
A_nid_ChsB       ITSEGL-DKFGFTNTSTERTSRFFQALLWSNAVVALIRFIGATWFLGKTGLLCCF--ARR 916
                 .*. ..   : .          :*  :*::.. :: .* .*  :*: ::.:       :*
```
