# Supplementary material for: A Novel Pathosystem With the Model Plant Arabidopsis thaliana for Defining the Molecular Basis of Taphrina Infections
Source: Environ Microbiol Rep. 2025 Jun 10;17(3):e70118. doi: 10.1111/1758-2229.70118 (PMC12152203; doi:10.1111/1758-2229.70118)
Supplement: Supplementary file 15 — TABLE S1. Receptor‐like kinase (RLK) and receptor‐like protein (RLP) lines included in the reverse genetic screen. [file EMI4-17-e70118-s003.pdf]

Table S1. Receptor-like kinase (RLK) and receptor-like protein (RLP) lines included in the reverse genetic screen.

Set	No.	Mutant name	Mutant line	Gene name	ATG code	Lectin domain	Source	References*
Known receptors/co-receptors	1	*cerk1-2*	GABI-Kat 096F09	*CERK1*	AT3G21630	LysM RLK	Cyril Zipfel	
	2	*lyk4 lyk5-2*	WiscDsLox297300_01c; SALK_131911c	*LYK4, LYK5*	AT2G23770; AT2G33580	-	Hugo Mélida	
	3	*lyk4*	WiscDsLox297300_01c; CS850683	*LYK4*	AT2G23770	LysM RLK	Christian Staehelin	
	4	*lyk5-2*	SALK_131911c	*LYK5*	AT2G33580	LysM RLK	Christian Staehelin	
	5	*bak1-5*	bak1-5 dCAPS line	*BAK1*	AT4G33430	LRR RLK	Cyril Zipfel	Xin *et al*., Nature 2016
	6	*bkk1-1*	SALK_057955	*BKK1*	AT2G13790	LRR RLK	Cyril Zipfel	
	7	*bak1-5 bkk1-1 cerk1-2*	*bbc* TM	*BAK1, BKK1, CERK1*	AT4G33430; AT2G13790; AT3G21630	-	Cyril Zipfel	
	8	*sobir1*	SALK_050715.49.20.x	*SOBIR1*	AT2G31880	LRR RLK	NASC N2107893	
	9	*fls2*	SAIL_691C04	*FLS2*	AT5G46330	LysM RLK	Cyril Zipfel	
	10	LYM1	SALK_094404c	LYM1	AT1G21880	LysM RLK	NASC N653591	
	11	LYM3_m1	SALK_111212c	LYM3	AT1G77630	LysM RLK	NASC N663666	
	12	LYM3_m2	SALK_132566c	LYM3	AT1G77630	LysM RLK	NASC N664031	
	13	DORN1	SALK_042209	LECRK-I.9	AT5G60300	L-type RLK	Klaas Bouwmeester	Wang *et al*., 2014
L-type RLKs/RLPs	14	AT3G09035	SALK_097008c	AT3G09035	AT3G09035	L-type RLP	NASC N678863	
	15	LECRK-I.1_m1	SALK_0521236c	LECRK-I.1	AT3G45330	L-type RLK	NASC N685929	
	16	LECRK-I.1_m2	SALK_069415c	LECRK-I.1	AT3G45330	L-type RLK	Klaas Bouwmeester	Wang *et al*., 2014
	17	LECRK-I.2	SAIL_847_F07	LECRK-I.2	AT3G45390	L-type RLK	NASC N862791	
	18	LECRK-I.3_m1	SALK_087804c	LECRK-I.3	AT3G45410	L-type RLK	Klaas Bouwmeester	Wang *et al*., 2014
	19	LECRK-I.3_m2	SALK_089591c	LECRK-I.3	AT3G45410	L-type RLK	Klaas Bouwmeester	Wang *et al*., 2014
	20	LECRK-I.7	SAIL_717_B11	LECRK-I.7	AT5G60270	L-type RLK	Klaas Bouwmeester	Wang *et al*., 2014
	21	LECRK-I.8_m1	SALK_147846c	LECRK-I.8	AT5G60280	L-type RLK	Klaas Bouwmeester	Wang *et al*., 2014
	22	LECRK-I.8_m2	SALK_066416c	LECRK-I.8	AT5G60280	L-type RLK	Klaas Bouwmeester	Wang *et al*., 2014
	23	LECRK-I.10	SALK_088366	LECRK-I.10	AT5G60310	L-type RLK	Klaas Bouwmeester	Wang *et al*., 2014
	24	LECRK-III.1_m1	SALK_149292	LECRK-III.1	AT2G29220	L-type RLK	Klaas Bouwmeester	Wang *et al*., 2014
	25	LECRK-III.1_m2	SALK_070736c	LECRK-III.1	AT2G29220	L-type RLK	NASC N686240	

Set	No.	Mutant name	Mutant line	Gene name	ATG code	Lectin domain	Source	References*
	26	LECRK-III.2	SALK_010738	LECRK-III.2	AT2G29250	L-type RLK	Klaas Bouwmeester	Wang *et al.*, 2014
	27	LECRK-IV.1_m1	SALK_019496c	LECRK-IV.1	AT2G37710	L-type RLK	Klaas Bouwmeester	Wang *et al.*, 2014
	28	LECRK-IV.1_m2	SALK_053703c	LECRK-IV.1	AT2G37710	L-type RLK	Klaas Bouwmeester	Wang *et al.*, 2014
	29	LECRK-IV.2	SALK_199402c	LECRK-IV.2	AT3G53810	L-type RLK	Klaas Bouwmeester	Wang *et al.*, 2014
	30	LECRK-IV.4_m1	SALK_077767c	LECRK-IV.4	AT4G02420	L-type RLK	Klaas Bouwmeester	Wang *et al.*, 2014
	31	LECRK-IV.4_m2	SALK_128001c	LECRK-IV.4	AT4G02420	L-type RLK	Klaas Bouwmeester	Wang *et al.*, 2014
	32	LECRK-V.1	SALK_071152c	LECRK-V.1	AT1G70110	L-type RLK	NASC N671496	
	33	LECRK-V.2	SALK_014678c	LECRK-V.2	AT1G70130	L-type RLK	Klaas Bouwmeester	Wang *et al.*, 2014
	34	LECRK-V.3	SALK_013197c	LECRK-V.3	AT2G43690	L-type RLK	Klaas Bouwmeester	Wang *et al.*, 2014
	35	LECRK-V.4	SALK_119422c	LECRK-V.4	AT2G43700	L-type RLK	Klaas Bouwmeester	Wang *et al.*, 2014
	36	LECRK-V.5	SALK_133163c	LECRK-V.5	AT3G59700	L-type RLK	Klaas Bouwmeester	Wang *et al.*, 2014
	37	LECRK-V.6_m1	SALK_009623c	LECRK-V.6	AT3G59730	L-type RLK	Klaas Bouwmeester	Wang *et al.*, 2014
	38	LECRK-V.6_m2	SALK_083045c	LECRK-V.6	AT3G59730	L-type RLK	Klaas Bouwmeester	Wang *et al.*, 2014
	39	LECRK-V.7	SALK_151041	LECRK-V.7	AT3G59740	L-type RLK	Klaas Bouwmeester	Wang *et al.*, 2014
	40	LECRK-V.8	WiscDsLox381A8	LECRK-V.8	AT3G59750	L-type RLK	Klaas Bouwmeester	Wang *et al.*, 2014
	41	LECRK-V.9_m1	SAIL_917_A03	LECRK-V.9	AT4G29050	L-type RLK	Klaas Bouwmeester	Wang *et al.*, 2014
	42	LECRK-V.9_m2	SALK_092640c	LECRK-V.9	AT4G29050	L-type RLK	Klaas Bouwmeester	Wang *et al.*, 2014
	43	LECRK-VI.1	SALK_058958c	LECRK-VI.1	AT3G08870	L-type RLK	Klaas Bouwmeester	Wang *et al.*, 2014
	44	LECRK-VI.2_m1	SALK_877782	LECRK-VI.2	AT5G01540	L-type RLK	Klaas Bouwmeester	Wang *et al.*, 2014
	45	LECRK-VI.2_m2	SALK_70801	LECRK-VI.2	AT5G01540	L-type RLK	Klaas Bouwmeester	Wang *et al.*, 2014
	46	LECRK-VI.3	SALK_108000c	LECRK-VI.3	AT5G01550	L-type RLK	Klaas Bouwmeester	Wang *et al.*, 2014
	47	LECRK-VI.4_m1	SALK_026891c	LECRK-VI.4	AT5G01560	L-type RLK	Klaas Bouwmeester	Wang *et al.*, 2014
	48	LECRK-VI.4_m2	SAIL_170_F02	LECRK-VI.4	AT5G01560	L-type RLK	NASC N862498	
	49	LECRK-VII.1_m1	SALK_093876c	LECRK-VII.1	AT4G04960	L-type RLK	Klaas Bouwmeester	Wang *et al.*, 2014
	50	LECRK-VII.1_m2	SALK_051149c	LECRK-VII.1	AT4G04960	L-type RLK	NASC N681136	
	51	LECRK-VII.2	SALK_141841c	LECRK-VII.2	AT4G28350	L-type RLK	Klaas Bouwmeester	Wang *et al.*, 2014
	52	LECRK-VIII.1_m1	SALK_056492c	LECRK-VIII.1	AT3G53380	L-type RLK	Klaas Bouwmeester	Wang *et al.*, 2014
	53	LECRK-VIII.1_m2	SALK_092365c	LECRK-VIII.1	AT3G53380	L-type RLK	Klaas Bouwmeester	Wang *et al.*, 2014
	54	LECRK-VIII.2_m1	SALK_051706c	LECRK-VIII.2	AT5G03140	L-type RLK	Klaas Bouwmeester	Wang *et al.*, 2014
	55	LECRK-VIII.2_m2	SALK_053278c	LECRK-VIII.2	AT5G03140	L-type RLK	Klaas Bouwmeester	Wang *et al.*, 2014

Set	No.	Mutant name	Mutant line	Gene name	ATG code	Lectin domain	Source	References*
	56	LECRK-IX.1_m1	SALK_042414c	LECRK-IX.1	AT5G10530	L-type RLK	Klaas Bouwmeester	Wang *et al.*, 2014
	57	LECRK-IX.1_m2	SALK_127554c	LECRK-IX.1	AT5G10530	L-type RLK	Klaas Bouwmeester	Wang *et al.*, 2014
	58	LECRK-IX.1_m3	SALK_149005c	LECRK-IX.1	AT5G10530	L-type RLK	NASC N655197	
	59	LECRK-IX.2	SALK_111817c	LECRK-IX.2	AT5G65600	L-type RLK	Klaas Bouwmeester	Wang *et al.*, 2014
	60	LECRK-S.1	SALK_033248c	LECRK-S.1	AT1G15530	L-type RLK	NASC N665711	
	61	LECRK-S.2	SALK_064192c	LECRK-S.2	AT2G32800	L-type RLK	NASC N653258	
	62	LECRK-S.4_m1	SALK_059967c	LECRK-S.4	AT3G55550	L-type RLK	Klaas Bouwmeester	Wang *et al.*, 2014
	63	LECRK-S.4_m2	SAIL_795_F11	LECRK-S.4	AT3G55550	L-type RLK	Klaas Bouwmeester	Wang *et al.*, 2014
	64	LECRK-S.5	SALK_088076c	LECRK-S.5	AT5G06740	L-type RLK	Klaas Bouwmeester	Wang *et al.*, 2014
	65	LECRK-S.6	SALK_151974c	LECRK-S.6	AT5G42120	L-type RLK	Klaas Bouwmeester	Wang *et al.*, 2014
	66	LECRK-S.7_m1	SALK_140480c	LECRK-S.7	AT5G55830	L-type RLK	NASC N677174	
	67	LECRK-S.7_m2	SALK_008479c	LECRK-S.7	AT5G55830	L-type RLK	NASC N679294	
G-type RLKs/RLPs	68	AT4G21390_m1	SALK_099394c	AT4G21390	AT4G21390	G-type RLK	NASC N663452	
	69	AT4G21390_m2	SALK_147351c	AT4G21390	AT4G21390	G-type RLK	NASC N664269	
	70	RFO3	SALK_136842c	RFO3	AT3G16030	G-type RLK	NASC N672169	
	71	RLK1_m1	SALK_084958c	RLK1	AT5G60900	G-type RLK	NASC N679906	
	72	RLK1_m2	SALK_146545c	RLK1	AT5G60900	G-type RLK	NASC N698380	
	73	SD1-13_m1	SALK_099776c	SD1-13	AT1G11350	G-type RLK	NASC N682709	
	74	SD1-13_m2	SALK_026338c	SD1-13	AT1G11350	G-type RLK	NASC N685512	
	75	RDA2	SALK_143489c	RDA2	AT1G11330	G-type RLK	NASC N677232	
	76	EGM1	SALK_058300	EGM1	AT1G11300	G-type RLK	NASC N682396	
	77	GAL2_m1	SALK_144144c	GAL2	AT1G78860	G-type RLP	NASC N660636	
	78	GAL2_m2	SALK_021225c	GAL2	AT1G78860	G-type RLP	NASC N660343	
Malectin type RLKs/RLPs	79	AT1G05700	SALK_025603c	AT1G05700	AT1G05700	Malectin RLK	NASC N674179	
	80	AT1G07560	SALK_058394c	AT1G07560	AT1G07560	Malectin RLK	NASC N655078	
	81	AT1G51790	SALK_025281c	AT1G51790	AT1G51790	Malectin RLK	NASC N670442	
	82	AT1G51860	SALK_035831c	AT1G51860	AT1G51860	Malectin RLK	NASC N665768	
	83	AT1G51880	SALK_109605c	AT1G51880	AT1G51880	Malectin RLK	NASC N671869	
	84	AT2G28970	SALK_142677c	AT2G28970	AT2G28970	Malectin RLK	NASC N664203	
	85	AT2G28990	SALK_051317c	AT2G28990	AT2G28990	Malectin RLK	NASC N669693	

Set	No.	Mutant name	Mutant line	Gene name	ATG code	Lectin domain	Source	References*
	86	AT3G46270	SALK_035789c	AT3G46270	AT3G46270	Malectin RLK	NASC N678352	
	87	AT3G46280_m1	SALK_132865c	AT3G46280	AT3G46280	Malectin RLK	NASC N664037	
	88	AT3G46280_m2	SALK_098065c	AT3G46280	AT3G46280	Malectin RLK	NASC N666849	
	89	AT3G46340	SALK_040269c	AT3G46340	AT3G46340	Malectin RLK	NASC N662189	
	90	AT3G46400	SALK_060803c	AT3G46400	AT3G46400	Malectin RLK	NASC N662661	
	91	AT4G20450	SALK_148633c	AT4G20450	AT4G20450	Malectin RLK	NASC N656063	
	92	AT5G16900	SALK_113523c	AT5G16900	AT5G16900	Malectin RLK	NASC N667068	
	93	AT5G48740	SALK_148236c	AT5G48740	AT5G48740	Malectin RLK	NASC N667583	
	94	AT5G59680	SALK_001929c	AT5G59680	AT5G59680	Malectin RLK	NASC N654468	
	95	CAP1	SALK_083442c	CAP1	AT5G61350	Malectin RLK	NASC N666567	
	96	HERK2	SALK_105055c	HERK2	AT1G30570	Malectin RLK	NASC N663563	
	97	*ios1*-1	GT_5_22250	IOS1	AT1G51800	Malectin RLK	Laurent Zimmerli	Yeh *et al.*, 2016.
	98	*ios1*-2	SALK_137388	IOS1	AT1G51800	Malectin RLK	Laurent Zimmerli	
	99	*ios1*-3	SAIL_343_B11	IOS1	AT1G51800	Malectin RLK	Laurent Zimmerli	
	100	MDS2_m1	SALK_115434c	MDS2	AT5G39000	Malectin RLK	NASC N660120	
	101	MDS2_m2	SALK_152141c	MDS2	AT5G39000	Malectin RLK	NASC N661219	
	102	MEE39_m1	SALK_065070c	MEE39	AT3G46330	Malectin RLK	NASC N657653	
	103	MEE39_m2	SALK_108641c	MEE39	AT3G46330	Malectin RLK	NASC N657769	
	104	RLP4_m1	SALK_085249c	RLP4	AT1G28340	Malectin/LRR RLP	NASC N672807	
	105	RLP4_m2	SALK_039264c	RLP4	AT1G28340	Malectin/LRR RLP	NASC N674570	
Jacalin type RLPs	106	AT1G52060_m1	SALK_108616c	AT1G52060	AT1G52060	Jacalin RLP	NASC N678939	
	107	AT1G52060_m2	SALK_151007c	AT1G52060	AT1G52060	Jacalin RLP	NASC N679251	
	108	AT1G52070	SALK_125442c	AT1G52070	AT1G52070	Jacalin RLP	NASC N625442	

***References:**

Xin *et al*. Bacteria establish an aqueous living space in plants crucial for virulence. Nature 539, 524–529 (2016). https://doi.org/10.1038/nature20166

Yeh *et al*. The *Arabidopsis* Malectin-Like/LRR-RLK IOS1 Is Critical for BAK1-Dependent and BAK1-Independent Pattern-Triggered Immunity. The Plant Cell, Volume 28, Issue 7, 1701-1721 (2016). https://doi.org/10.1105/tpc.16.00313

Wang *et al*. Phenotypic Analyses of *Arabidopsis* T-DNA Insertion Lines and Expression Profiling Reveal That Multiple L-Type Lectin Receptor Kinases Are Involved in Plant Immunity MPMI Vol. 27, No. 12, 2014, pp. 1390–1402. http://dx.doi.org/10.1094/MPMI-06-14-0191-R
