# Supplementary material for: A Novel Pathosystem With the Model Plant Arabidopsis thaliana for Defining the Molecular Basis of Taphrina Infections
Source: Environ Microbiol Rep. 2025 Jun 10;17(3):e70118. doi: 10.1111/1758-2229.70118 (PMC12152203; doi:10.1111/1758-2229.70118)
Supplement: Supplementary file 16 — TABLE S2. Primers used for genotyping and qPCR. [file EMI4-17-e70118-s015.pdf]

Table S2. Primers used for genotyping and qPCR.

A. Genotyping

Experiment	Target	Mutant line	AGI code	Forward	Reverse	T-DNA primer or enzyme
Known receptor/ co-receptor mutants	*cerk1*-2	GABI-Kat 096F09	AT3G21630	ATGAAGCTAAAGATTTCTCTAAT	CCGGCCGGACATAAGACTGACT	ATATTGACCATCATACTCATTGC (o8409)
	lyk4	WiscDsLox297300_01c	AT2G23770	CCACAATCGGTTTCTCCTCCTCCATTGTC	GTACGACGATTCTTCCCAGTTCTGCGTAG	AACGTCCGCAATGTGTTATTAAGTTGTC (P745)
	lyk5-2	SALK_131911c	AT2G33580	CATGGCTGCGTGTACACTCCACG	GTTGCCAAGAGAGCCGGAACGAAG	ATTTTGCCGATTTCGGAAC (LBb1.3)
	bak1-5	*bak1*-5 dCAPS (SNP) NASC N799997	AT4G33430	AAGAGGGCTTGCGTATTTACATGATCAGT	GTCCAAAATCCCCAACCACG	RsaI (*bak1*-5 derived PCR products contain an additional RsaI site)
	bkk1-1	SALK_057955c	AT2G13790	TGGCTCAGAAGAAAACCACAG	CTGCTCCACTTCTGTTTCCAC	ATTTTGCCGATTTCGGAAC (LBb1.3)
	sobir1	SALK_050715.49.20.x	AT2G31880	CAAGCAATAAGAGGATCAGAAAAAC	GGATCATCCAAAGTACAGTAACAGG	ATTTTGCCGATTTCGGAAC (LBb1.3)
	fls2	SAIL_691C04	AT5G46330	ACATGTCCGGTACTATCGCAG	TCCATCAAGACAGCTAATGAGC	GCTTCCTATTATATCTTCCCAAATTACCAATACA (Lb2 JK455)
Mutants from the primary screen	LECRK-IV.1_m1	SALK019496c	AT2G37710	CTCTAACAGCCGTAACAAGCG	CGTCGAATCTTCCATCATCAC	ATTTTGCCGATTTCGGAAC (LBb1.3)
	SD1-13_m1	SALK099776c	AT1G11350	ATAACCGCTGTGGTGTTATGC	TATGCAGGAGTTTTCTGGCAC	ATTTTGCCGATTTCGGAAC (LBb1.3)
	SD1-13_m2	SALK026338c	AT1G11350	ACAGTAACAGCCCGATCAATG	GCGATCTTCCACAGTGCTAAG	ATTTTGCCGATTTCGGAAC (LBb1.3)
	ios1-1	Ds transposon GT_5_22250	AT1G51800	CAACCACGAACGAGACCGAAG	GCCGTAAGCCGACTTGATGTTC	*ios1*-1 lacks PCR product

B. qPCR

Target	Target gene name	Marker of	AGI code	Forward	Reverse	Efficiency
Immunity marker genes	*CBp60g*	SA response	AT5G26920	GGAGACACACAACTGCCAGA	TATCGTGCAACGCAAGAAAC	1.708
	ICS1/SID2	SA response	AT1G74710	GCTTGGCTAGCACAGTTACAGC	CACTGCAGACACCTAATTGAGTCC	1.928
	PR1	SA response	AT2G14610	CGGAGCTACGCAGAACAACT	CTCGCTAACCCACATGTTCA	1.658
	CEJ1	JA response	AT3G50260	GGTGGTGCAGAACAACAAACGC	AATAACCCGCCGCCAAAGTCAC	2
	JAZ1	JA response	AT1G19180	CGTGGCTCGGTTTAGCAG	TGAAGCAACGTCGTCAAAAG	1.801
	PDF1.2	JA response	AT5G44420	CCAAACATGGATCATGCAAC	CACACGATTTAGCACCAAAGA	1.698
	PAD3	Camalexin	AT3G26830	GATGTTCCTGCGAAAACACA	GTTTTGGATCACGACCCATC	1.646
	CYP71A13	Camalexin	AT2G30770	GGGTAGAGGCTGGACCAAAT	ACAACCGAAGATGGAAATGC	1.957
	WRKY40	PTI	AT1G80840	CTTGACTGTGCCGGTGACTA	TCTGAACTTGGGGAAAATCG	1.924
Reference genes	*PP2AA3*	-	AT1G13320	GCGGTTGTGGAGAACATGATACG	GAACCAAACACAATTCGTTGCTG	1.854
	TIP41	-	AT4G34270	GTGAAAACTGTTGGAGAGAAGCAA	TCAACTGGATACCCTTTCGCA	1.867
	YLS8	-	AT5G08290	TTACTGTTTCGGTTGTTCTCCATTT	CACTGAATCATGTTCGAAGCAAGT	1.822
