# Supplementary material for: A Novel Pathosystem With the Model Plant Arabidopsis thaliana for Defining the Molecular Basis of Taphrina Infections
Source: Environ Microbiol Rep. 2025 Jun 10;17(3):e70118. doi: 10.1111/1758-2229.70118 (PMC12152203; doi:10.1111/1758-2229.70118)
Supplement: Supplementary file 17 — TABLE S3. Carbon assimilation by Taphrina tormentillae strains. [file EMI4-17-e70118-s002.pdf]

Table S3. Carbon assimilation by *Taphrina tormentillae* strains. Yeast cells were grown in API® 50 CH strips from bioMérieux suspended in yeast nitrogen base (YNB) media from US Biological (defined media without carbohydrate, with amino acids and with ammonium sulfate). Growth was visually assessed after 21 days and scored as very strong (+++), strong (++), medium (+), weak (w), weak to none (?), clearly absent (-). Experiment was replicated three times and summarized results are presented (full experimental data availble in Data S1). Carbohydrates with clear, replicable difference in assimilation capacity among the tested *T. tormentillae* strains are in bold. Bottom part of the table shows carbohydrate assimilation pattern for different *T. tormentillae* strain found in literature. Strains used in Fonseca and Rodrigues, 2011 - PYCC 5705 (CBS 332.55); Inácio *et al*., 2004 - PYCC 5705 (CBS 332.55) and PYCC 5727(CBS 339.55); Petrýdesová *et al*., 2016 - PYCC 5727 (CBS 339.55T), CBS 311.31, CCY 058-008-001, CCY 058-008-002, CCY 058-008-003, CCY 058-008-004, CCY 058-008-005, CCY 058-008-006.

	M11	PYCC 5705	PYCC 5727	Fonseca & Rodrigues, 2011	Inácio *et al*., 2004	Petrýdesová *et al*., 2016
Glycerol	++	++	++	+		
Erythritol	-	-	-	-		
D-Arabinose	+	w	w	+	-	
L-Arabinose	+	w	+	+		
D-Ribose	-	-	-	-	-	
D-Xylose	++	++	++	+	+	
**L-Xylose**	**w**	**-**	**-**			
D-Adonitol	-	?	-			
**Methyl-ß-D-xylopyranoside**	**-**	**-**	**+**			
D-Galactose	++	+	++	+	+	+
D-Glucose	++	++	++	+		+
D-Fructose	++	++	++			
D-Mannose	++	++	+++			
L-Sorbose	-	-	-	v		-/w
L-Rhamnose	-	-	-	-		
Dulcitol	-	-	-			
Inositol	-	-	-	-	-	
D-Mannitol	+	+	w	v		
D-Sorbitol	++	+	++		+	
Methyl-α-D-mannopyranoside	-	-	-			
Methyl-α-D-glucopyranoside	-	-	-		-	
N-Acetylglucosamine	-	-	-	n		
Amygdalin	-	-	-			

	M11	PYCC 5705	PYCC 5727	Fonseca & Rodrigues, 2011	Inácio *et al.*, 2004	Petrýdesová *et al.*, 2016
**Arbutin**	**w**	**-**	**?**			
Esculin Ferric citrate	+++	++	+++			
Salicin	?	w	?	+		-
D-Cellobiose	++	++	++	+		+
**D-Maltose**	**++**	**w**	**?**	**-**	**-**	**-**
D-Lactose (bovine origin)	-	-	-	-		-
D-Melibiose	-	-	-	-		-
D-Saccharose (sucrose)	++	++	++	+	+	+
D-Trehalose	++	+	+	+		+
Inulin	?	?	-	+		-
**D-Melezitose**	**w**	**-**	**-**	**-**	**-**	**-**
D-Raffinose	+	++	+	+	+	+
**Starch (amidon)**	**+++**	**++**	**+**	**+**		**+**
**Glycogen**	**w**	**-**	**?**			
Xylitol	+	+	w	+	+	
Gentiobiose	++	++	++			
D-Turanose	-	-	-			
**D-Lyxose**	**+**	**-**	**?**			
D-Tagatose	-	-	-			
**D-Fucose**	**-**	**-**	**w**			
L-Fucose	-	-	-			
D-Arabitol	+	++	++			
**L-Arabitol**	**+**	**?**	**?**			
Potassium gluconate	++	+++	+++	+		
Potassium 2-ketogluconate	w	+	+			
Potassium 5-ketogluconate	++	+++	+++			

References:

Fonseca, Á., and Rodrigues, M. G. (2011). *Taphrina* fries (1832). In The Yeasts (pp. 823-858). Elsevier.

Inácio, J. *et al.* (2004). Characterisation and classification of phylloplane yeasts from Portugal related to the genus *Taphrina* and description of five novel *Lalaria* species. FEMS yeast research, 4(4-5), 541-555.

Petrýdesová, J. *et al.* (2016). Disentangling identity of species of the genus *Taphrina* parasitizing herbaceous *Rosaceae*, with proposal of *Taphrina gei-montani* sp. nov. International Journal of Systematic and Evolutionary Microbiology, 66(7), 2540-2549.
