# Supplementary material for: A Novel Pathosystem With the Model Plant Arabidopsis thaliana for Defining the Molecular Basis of Taphrina Infections
Source: Environ Microbiol Rep. 2025 Jun 10;17(3):e70118. doi: 10.1111/1758-2229.70118 (PMC12152203; doi:10.1111/1758-2229.70118)
Supplement: Supplementary file 18 — TABLE S4. Taphrina tormentillae distribution. [file EMI4-17-e70118-s012.pdf]

Table S4. *Taphrina tormentillae* distribution.

A. *T. tormentillae* on *Arabidopsis*. Identified from studies where sequences similar to the *Taphrina* strain M11 ITS were found. Identity and gaps are for alignments of *Taphrina* M11 ITS sequence with OTU sequences from referenced studies. OTU sequences available in Data S2. ID (%) – % nucleotide identity; Ref – study reference.

OTU	ID (%)	Gaps	Findings	Source	Ref
F150	99.52	0/210	Heritable hub, not part of the core microbiome, prevalent in 4 out of 8 experiments	*Arabidopsis*, Sweden	Brachi *et al.*, 2022
F3841	99.05	1/211	-		
F7835	99.51	0/204	-		
F4195	98.1	0/210	-		
Otu00933	98.85	0/174	Occurrence on *A. thaliana* in field experiments varies by year. Year 1 - 17%, year 2 - 0%, and year 3 - 3%. Not part of the core microbiome.	*Arabidopsis*, Germany	Almario *et al.*, 2022

B. Data from Brachi *et al.*, 2022. Text in red - data on the closest OTU match to *Taphrina* M11. n/8 – the number of experiments (out of eight) where the corresponding trait was observed.

OTU	Heritable (n/8)	Hub (n/8)	Heritable hub (n/8)	Prevalence (n/8)	Core microbiota	Species
F147	2	0	0	5	no	*Taphrina carpini*
**F150**	**2**	**2**	**1**	**4**	**no**	***Taphrina tormentillae***
F174	1	1	0	4	no	*Taphrina carpini*
F626	0	0	0	0	no	*Taphrina padi*
F805	0	0	0	0	no	*Taphrina epiphylla*

C. Data from Almario *et al.*, 2022. Text in red - data on the closest OTU match to *Taphrina* M11. Core - part of the core microbiome; RA – relatilve abundance; OC - occurance; Y - year; Size - length of the OTU sequence in nucleotides.

OTU	Core	RA total	OC total	RA y1	RA y2	RA y3	OC y1	OC y2	OC y3	Size	Species
Ftrad_Otu_00389	no	6.03E-05	0.20	1.50E-04	2.44E-05	1.54E-05	0.34	0.17	0.10	316	*Taphrinomycetes* sp.
Ftrad_Otu_00964	no	1.15E-05	0.08	2.95E-05	9.81E-07	5.81E-06	0.19	0.03	0.04	64	*Taphrinaceae* sp.
Ftrad_Otu_00048	no	1.78E-03	0.81	3.44E-03	1.14E-03	9.29E-04	0.88	0.77	0.78	9804	*T. carpini*
Ftrad_Otu_00466	no	7.73E-05	0.05	1.59E-05	0.00E+00	2.07E-04	0.05	0.00	0.11	215	*T. padi*
**Ftrad_Otu_00933**	**no**	**1.21E-05**	**0.06**	**3.53E-05**	**0.00E+00**	**3.16E-06**	**0.17**	**0.00**	**0.03**	**68**	***T. tormentillae***

D. Environmental distribution of *Taphrina tormentillae*. Identified from presence of *T. tormentillae* ITS region sequences in environmental metgemoics data. NCBI database was searched using BLAST tool for *Taphrina tormentillae* strain M11 ITS region hits in June 2023. Searches performed with full length ITS sequences (containing ITS1, 5S rDNA, and ITS2), and recovered ITS sequences that were full lenth containing ITS1, 5.8S rRNA gene, and ITS2 (FULL), or partial (ITS1, or ITS2), as indicated. The criteria for positive hits here were similarity >97% and coverage >95%. For full search data see Data S2. Culutred "+" strains are available in cultrure colections.

Nr	Accessions	Source	Cultured	ITS
1	FN428580.1	*Tragopogon pratensis,* Germany	+	FULL
2-7	KX516408.1; KX516406.1; KX516379.1; KX516356.1; KX516339.1; KX516159.1	Rainwater, China	-	FULL
9-12	KU134810.1; KU134808.1; KU134807.1; KU134806.1	*Potentilla erecta,* Slovakia	-	FULL
13-29	KX516463.1; KX516459.1; KX516457.1; KX516441.1; KX516436.1; KX516431.1; KX516430.1; KX516407.1; KX516372.1; KX516364.1; KX516354.1; KX516352.1; KX516347.1; KX516344.1; KX516342.1; KX516333.1; KX516331.1	Rainwater, China	-	FULL
30-32	KX147846.1; KX147824.1; KX147815.1	Surface of pine sapwood treated with raw linseed oil	-	FULL
33-34	KU134809.1; KU134805.1	*Potentilla erecta,* Slovakia	+	FULL
36-43	KX516432.1; KX516418.1; KX516382.1; KX516375.1; KX516348.1; KX516337.1; KX516334.1; KX516236.1	Rainwater, China	-	FULL
45	KR698797.1	Arctic stream, Svalbard, Norway	-	FULL
46	KX147810.1	Surface of pine sapwood treated with raw linseed oil	-	FULL
48	MZ314479.1	*Cladonia stellaris,* Russia	+	FULL
49	MK782173.1	Wing surface of a *Myotis evotis* bat, western North America	-	FULL
50	MN836186.1	Soil from semi-arid temperate steppe, China	-	ITS1
51	MK722886.1	Soil from high-elevation, late-melting Navajo Peak in Colorado, USA	-	ITS1
52	MK718712.1	Unvegetated soil from high-elevation, late-melting Navajo Peak in Colorado, USA	-	ITS1
54	MH239610.1	Plant root (species information unavailable), high-elevation research site, Colorado, USA	-	ITS1
55	KX516239.1	Rainwater, China	-	ITS1
56	JF945653.1	*Fagus sylvatica* phyllosphere, southern France	-	ITS1

Nr	Accessions	Source	Cultured	ITS
58-68	LS952548.1; LS963834.1; LS963833.1; LS963832.1; LS963831.1; LS963830.1; LS963829.1; LS963828.1; LS963827.1; LS963826.1; LS963825.1	Soil from *Carex curvula* meadows of the European alpine system	-	ITS1
69	KF222467.1	Indoor surface swab, California, USA	-	ITS1
70	MN059913.1	Whim Bog, *Erica tetralix* roots, Scotland	-	ITS1
71	MF180946.1	Soil, Northeast Greenland	-	ITS2
72	KJ827949.1	Soil from Alaskan tundra, USA	-	ITS2
73	OX032890.1	Roots of *Festuca brevipila,* Germany: Brandenburg, Mallnow	-	ITS2
74-75	LR874746.1; LR874745.1	Wooded meadow soil, Estonia	-	ITS2
76	MZ441955.1	Needles of *Pinus peuce* in high-elevation region of Montenegro	-	ITS2
77	MN903602.1	Soil and needles of *Pinus sylvestris*, Lithuania	-	ITS2
78	KX147567.1	Surface of pine sapwood treated with raw linseed oil	-	ITS2
79	KP892365.1	*Scolytus multistriatus* beetle, Sweden	-	ITS2
80-81	MW076032.1; MW075993.1	*Vitis vinifera* bulk soil, Tokaj wine region, Hungary	-	ITS2
82	MW448958.1	*Empetrum nigrum* fruit endophyte, Oulu, Finland	-	ITS2
83	MW757709.1	Fungal spore trap (air), Lithuania	-	ITS2
84	MN151755.1	Sub-arctic tundra soil, Eight Mile Lake, USA	-	ITS2

References:

1. Brachi, B. *et al* (2022). Plant genetic effects on microbial hubs impact host fitness in repeated field trials. Proceedings of the National Academy of Sciences, 119(30), e2201285119. DOI: 10.1073/pnas.2201285119

2. Almario J. *et al* (2022). The Leaf Microbiome of Arabidopsis Displays Reproducible Dynamics and Patterns throughout the Growing Season. mBio 13:e02825-21. DOI: 10.1128/mbio.02825-21
