# Supplementary material for: A Novel Pathosystem With the Model Plant Arabidopsis thaliana for Defining the Molecular Basis of Taphrina Infections
Source: Environ Microbiol Rep. 2025 Jun 10;17(3):e70118. doi: 10.1111/1758-2229.70118 (PMC12152203; doi:10.1111/1758-2229.70118)
Supplement: Supplementary file 19 — TABLE S5. Taphrina strain M11 genome assembly statistics. [file EMI4-17-e70118-s007.pdf]

Table S5. *Taphrina* strain M11 genome assembly statistics. Genome assembly quality was analysed using QUAST tool, version 5.0. For additional explanation of QUAST output see (http://quast.sourceforge.net/). [a]These sequence data are available at the Genebank (NCBI) database under the given accession numbers. Abbreviations used: SRA, sequence read archive. [b]Genome completeness estimated using BUSCO.

BioProject[a]	PRJNA487587
BioSample[a]	SAMN09906266
SRA[a]	SRX4936057
Total length (bp)	13 601 285
Contigs (≥ 0 bp)	382
Total length of contigs (≥ 0 bp)	13 654 843
Contigs (≥ 1000 bp)	147
Total length of contigs (≥ 1000 bp)	13 544 983
Total contigs	234
Largest contig (bp)	514 056
Average GC (%)	48.83
N50	277 880
N75	157 923
L50	19
L75	34
# N's per 100 kbp	0.02
Annotated genes	6496
ORFs >100 aa	14 561
Density (ORFs >100 aa/kbp)	1.07
Genome completeness	96.97%
