# Supplementary material for: A Novel Pathosystem With the Model Plant Arabidopsis thaliana for Defining the Molecular Basis of Taphrina Infections
Source: Environ Microbiol Rep. 2025 Jun 10;17(3):e70118. doi: 10.1111/1758-2229.70118 (PMC12152203; doi:10.1111/1758-2229.70118)
Supplement: Supplementary file 20 — TABLE S6. Comparison of genome statistics and candidate effector proteins. [file EMI4-17-e70118-s021.pdf]

Table S6: Comparison of genome statistics and candidate effector proteins. Analysis was performed with *Taphrina* strain M11 and other fungi from the subphyla Taphrinomycotina and Saccharomycotina. Strains and genome assemblies used: *Taphrina deformans* PYCC 5710, CAHR02; *T. deformans* JCM 22205, BAVV01; *T. flavorubra* JCM 22207, BAVW01; *T. populina* JCM 22190, BAVX01; *T. wiesneri* JCM 22204, BAVU01; *Neolecta irregularis* DAH-3, LXFE01; *Pneumocystis murina* B123, AFWA02; *Schizosaccharomyces pombe* 972h-, ASM294v2; *S. cryophilus* OY26, ACQJ02; *S. japonicus* yFS275, AATM02; *S. octosporus* yFS286, ABHY03; *Saitoella complicata* NRRL Y-17804; *Saccharomyces cerevisiae* S288c, R64; *Yarrowia lipolytica* CLIB122, ASM252v1; *Candida albicans* SC5314, ASM18296v3. Secretion signals were identified using SignalP 4.1. Lifestyle abbreviations used: PP, plant pathogen; RA, rhizosphere associated; S, saprotroph; MP, mammalian pathogen; IA, insect associated.

Species/Strains	Genome size (Mbp)	GC (%)	Annotated genes	ORFs > 100 aa	ORF Density (>100 aa)/kbp	Short ORFs 80-333 aa	Short secreted proteins (80-333 aa)	Cys-rich proteins (C >= 4)	Lifestyle
Taphrina strain M11	13.6	48.8	6496	14561	1.07	18660	767	337	PP
Taphrina deformans	13.4	49.5	4663	14510	1.08	18081	881	421	PP
Taphrina deformans	13.8	49.5	6621	14769	1.07	18421	909	431	PP
Taphrina flavorubra	15.7	49.5	7192	16947	1.08	21829	1097	513	PP
Taphrina populina	12.0	47.4	6205	11633	0.97	14424	799	378	PP
Taphrina wiesneri	13.1	48.1	6320	12317	0.94	15314	756	334	PP
Neolecta irregularis	14.2	44.2	5536	12144	0.86	17954	892	354	S/RA
Pneumocystis murina	7.5	27.0	3675	3951	0.53	5154	136	26	MP
Schizosaccharomyces pombe	12.6	36.0	6991	6260	0.50	6077	248	65	S
Schizosaccharomyces cryophilus	11.6	37.7	5494	6387	0.55	6543	271	68	S
Schizosaccharomyces japonicus	11.7	43.8	5224	9011	0.77	10507	511	195	S
Schizosaccharomyces octosporus	11.6	37.5	5347	6274	0.54	6465	255	60	S
Saitoella complicata	14.1	52.6	7211	19353	1.37	24552	1018	322	IA
Saccharomyces cerevisiae	12.2	38.2	6445	7473	0.61	6367	440	100	S
Yarrowia lipolytica	20.6	49.0	7357	20544	1.00	26526	1499	541	S
Candida albicans SC5314	14.3	33.5	6263	7272	0.51	5756	392	126	MP
