# Supplementary material for: A Novel Pathosystem With the Model Plant Arabidopsis thaliana for Defining the Molecular Basis of Taphrina Infections
Source: Environ Microbiol Rep. 2025 Jun 10;17(3):e70118. doi: 10.1111/1758-2229.70118 (PMC12152203; doi:10.1111/1758-2229.70118)
Supplement: Supplementary file 21 — TABLE S7. Comparison of orthologs in selected Taphrinomycotina species. [file EMI4-17-e70118-s001.pdf]

Table S7. Comparison of orthologs in selected Taphrinomycotina species. Annotated proteins were analyzed using OrthoVenn2 web platform. M11 and *Protomyces arabidopsidicola* were isolated from wild *Arabidopsis* plants. Other included *Protomyces* species are known to infect plants in the families *Compositae* and *Umbelliferae*. *T. deformans, T. wiesneri*, and *T. flavorubra* are pathogenic on *Prunus* species. The host of *T. populina* is *Populus nigra*. *Schizosaccharomyces pombe* is phylogenetically most distant to other analyzed species and has a non-pathogenic lifestyle.

	**Clusters**	**Total proteins**	**M11**	***T. deformans***	***T. wiesneri***	***T. populina***	***T. flavorubra***	***P. arabidopsidicola***	***P. lactucaedebilis***	***P. gravidus***	***P. macrosporus***	***S. pombe***	**Average**	**%**
**Total (proteins)**	-	-	5808	5867	5630	5134	6297	5514	5952	5422	5912	5133	5666.9	100
**Total (clusters)**	-	-	5166	5407	5376	4735	5521	5206	5347	4979	5186	3262	5018.5	-
**All species**	2110	21836	2166	2198	2173	2165	2178	2169	2153	2164	2164	2306	2183.6	38.533
**Taphrinales specific**	829	7582	844	842	838	835	840	845	839	845	854	-	842.4	14.866
***Protomyces* specific**	388	1612	-	-	-	-	-	394	398	399	421	-	403	7.1115
***Taphrina* specific**	297	1496	298	299	299	300	300	-	-	-	-	-	299.2	5.2798
Prunus* specific *Taphrina	175	542	-	184	178	-	180	-	-	-	-	-	180.6	3.1881
**M11 and *Prunus* pathogens**	129	522	132	131	130	-	129	-	-	-	-	-	130.5	2.3028
**M11 and *Populus* pathogen**	16	41	20	-	-	21	-	-	-	-	-	-	20.5	0.3617
**Isolated from *Arabidopsis***	2	10	7	-	-	-	-	3	-	-	-	-	5	0.0882
**Other**	-	-	2341	2213	2012	1633	2850	2103	2562	2014	2473	2827	2302.8	40.636
**Species specific (proteins)**	-	1278	151	92	8	137	303	26	174	108	89	190	127.8	2.2552
**Species specific (clusters)**	369	-	39	17	4	39	58	11	58	38	39	66	36.9	0.6511
