# Supplementary material for: A Novel Pathosystem With the Model Plant Arabidopsis thaliana for Defining the Molecular Basis of Taphrina Infections
Source: Environ Microbiol Rep. 2025 Jun 10;17(3):e70118. doi: 10.1111/1758-2229.70118 (PMC12152203; doi:10.1111/1758-2229.70118)
Supplement: Supplementary file 22 — TABLE S8. Auxin and cytokinin biosynthesis pathways in Taphrina strain M11. [file EMI4-17-e70118-s004.pdf]

Table S8. Auxin and cytokinin biosynthesis pathways in *Taphrina* strain M11. [a]Auxin (IAA: indole acetic acid) biosynthesis pathway name abbreviations are the indole-3-acetamide (IAM), Tryptophan side chain oxidase (TSO), indole-3-pyruvate (IPyA), indole-3-acetonitrile (IAN), and tryptamine (TAM) pathways. [b]Here the number of hits meeting the criteria of bit scores > 120, E-values > 0.05, and identities > 50% are presented.

Hormone	Pathway[a]	Enzymes	Hits[b]
IAA	IAM pathway	Trp2-monooxygenase (TMO/IaaM)	1
		IAM hydrolase (IaaH)	2
	IAN pathway	Unknown enzymes	NA
		Nitrilase (NIT)	3
	IPyA pathway	Trp aminotransferase (TAM)	1
		IPyA decarboxylase (IPDC)	1
		IAAld dehydrogenase (IAD)	2
	TSO pathway	Tryptophan side-chain oxidase (TSO)	NA
		IAAld dehydrogenase (IAD)	2
	TAM pathway	Tryptophan decarboxylases (TDC)	2
		Amine oxidase (AOX)	3
		IAAld dehydrogenase (IAD)	2
		Flavin monooxygenase (YUC)	1
	other proteins	Auxin efflux carrier (1)	1
		Auxin efflux carrier (2)	1
Cytokinin	Isopentenyladenine dependent	tRNA-isopentenyltransferase	1
		Cytokinin phosphoribohydrolase	1
