# Supplementary material for: A Novel Pathosystem With the Model Plant Arabidopsis thaliana for Defining the Molecular Basis of Taphrina Infections
Source: Environ Microbiol Rep. 2025 Jun 10;17(3):e70118. doi: 10.1111/1758-2229.70118 (PMC12152203; doi:10.1111/1758-2229.70118)
Supplement: Supplementary file 23 — TABLE S9. M11 Taphrina auxin (IAA: indole‐3‐acetic acid) biosynthesis pathways data. [file EMI4-17-e70118-s017.pdf]

Table S9. M11 *Taphrina* auxin (IAA: indole-3-acetic acid) biosynthesis pathways data. Analysis identified IAA biosynthesis genes of *Taphrina* species strain M11. Genes were identified using potential IAA biosynthesis genes from other fungal species as BLAST search terms, as in (Wang *et al*., 2019). Pathway name abbreviations are indole-3-acetamide (IAM), indole-3-pyruvate (IPyA), indole-3-acetonitrile (IAN), and tryptamine (TAM) pathways.

IAA pathway	M11 enzyme	Hit sequence ID	Bits scores	E-value	Identities
IAM pathway	TMO (IaaM)	NODE_17_length_282705_cov_10.4592_ID_33	624	0.0	74%
	IaaH1	NODE_18_length_280144_cov_10.6475_ID_35	308	3.00E-169	61%
	IaaH2	NODE_1_length_514056_cov_10.365_ID_1	481	3.00E-153	55%
IAN pathway	NIT1	NODE_29_length_215297_cov_10.5838_ID_61	91.7	7.00E-21	58%
	NIT2	NODE_6_length_394779_cov_10.7723_ID_11	410	2.00E-131	68%
	NIT3	NODE_12_length_327303_cov_10.5206_ID_23	281	9.00E-87	51%
IPyA pathway	IPDC	NODE_44_length_123889_cov_10.6904_ID_87	810	0.0	71%
	Iad1	NODE_8_length_361341_cov_10.7402_ID_15	536	5.00E-172	60%
	Iad2	NODE_8_length_361341_cov_10.7402_ID_15	536	5.00E-172	60%
		NODE_15_length_296775_cov_10.4422_ID_29	454	2.00E-143	47%
	TAM1				
	TAM2	NODE_15_length_296775_cov_10.4422_ID_29	459	0.0	80%
TAM pathway	AOx1	NODE_22_length_257679_cov_10.5919_ID_47	1201	0.0	80%
	AOx2	NODE_20_length_270696_cov_10.5006_ID_39	1018	0.0	68%
	AOx3	NODE_20_length_270696_cov_10.5006_ID_39	1018	0.0	68%
		NODE_188_length_622_cov_0.640404_ID_375	145	2.00E-40	54%
	TDC1	NODE_22_length_257679_cov_10.5919_ID_47	815	0.0	81%
		NODE_188_length_622_cov_0.640404_ID_375	145	2.00E-40	54%
	TDC2	NODE_13_length_326311_cov_11.2801_ID_25	635	0.0	64%
	Iad1	NODE_8_length_361341_cov_10.7402_ID_15	536	5.00E-172	60%
	Iad2	NODE_8_length_361341_cov_10.7402_ID_15	536	5.00E-172	60%
		NODE_15_length_296775_cov_10.4422_ID_29	454	2.00E-143	47%
	YUC	NODE_5_length_408885_cov_10.082_ID_9	267	5.00E-173	83%
