# Supplementary material for: A Novel Pathosystem With the Model Plant Arabidopsis thaliana for Defining the Molecular Basis of Taphrina Infections
Source: Environ Microbiol Rep. 2025 Jun 10;17(3):e70118. doi: 10.1111/1758-2229.70118 (PMC12152203; doi:10.1111/1758-2229.70118)
Supplement: Supplementary file 24 — TABLE S10. Putative cell wall biosynthesis and chitin modification genes in Taphrina strain M11. [file EMI4-17-e70118-s019.pdf]

Table S10. Putative cell wall biosynthesis and chitin modification genes in *Taphrina* strain M11. Genes were idntified based on the putative cell wall biosynthesis genes. Blast protein search was performed with default search parameters against the *Taphrina* M11 genome using Unipro UGENE software (Okonechnikov *et al*. 2012) . Sequences of well-described homologs from *S. cerevisiae, S. pombe*, and *A. nidulans* were used as protein blast queries. *Taphrina* M11 gene hits were confirmed by using NCBI BLASTp tool against non-redundant UniProtKB/SwissProt sequences. Additionally, all predicted genes containing conserved pfam domains specific for the cell wall biosynthesis genes were analyzed to confirm their identity. Query genes and conserved domains used are listed in Data S7. [a] Biochemical evidence (BE) from study by Petit and Schneider, 1983. [b.] Very small amount of glucosamine was detected, but was < 0.2% of wall monosaccharides and could have originated from cell wall glycoproteins. [c.] See Data S7 for corrected sequence. [d] α-1,3-glucan [e]Mannose, galactose, and rhamnose mono-saccharides.

Product	Gene	Accession	Function (inferred from homology)	BE[a]
Chitin	*Chs1*	TM11_g683.t1	Chitin synthase, class I. Polymerizes UDP-N-acetylglucosamine into chitin.	No[b]
	Chs2	TM11_g677.t1	Chitin synthase, class III. Polymerizes UDP-N-acetylglucosamine into chitin.	
	Cfr1-like	TM11_g3567.t1	*S. pombe homolog Cfr1 is not involved in chitin biosynthesis; S. cerevisiae homolog Chs5* regulates *Chs3* activity.	
	Bch1	TM11_g5773.t1	*S. pombe* homolog *Bch1* is not involved in chitin biosynthesis; *S. cerevisiae* homolog *Chs6* regulates *Chs3* activity.	
	Cts1	TM11_g5571.t1	LysM domain containing chitinase, remodelling of chitin.	
	Cts2	TM11_g4787.t1[c]	CMB19 domain containing chitinase, remodelling of chitin.	
	Crr1	TM11_g2933.t1	Chitin crosslinking	
	Crr2	TM11_g4569.t1	Chitin crosslinking	
	Crh1	TM11_g3026.t1	Chitin crosslinking	
Chitosan	*Cda1*	TM11_g2542.t1	Chitin deacetylase.	Not tested
α-glucan	*Ags1*<br>*Ags2*	TM11_g3195.t1<br>TM11_g396.t1[c]	α-1,3-glucan/α-1,4-glucan synthase.	Yes[d]
β-1,3-glucan	*Gas1*<br>*Gas2*<br>*Gas3*<br>*Gas4*	TM11_g1673.t1<br>TM11_g762.t1<br>TM11_g2885.t1<br>TM11_g1143.t1[c]	β-1,3-glucanosyltransferase. Elongation of β-1,3-glucan chains.	Yes

Product	Gene	Accession	Function (inferred from homology)	BE[a]
	Fks1	TM11_g3331	Catalytic subunit of β-1,3-glucan synthase complex	
	Rho1	TM11_g2469.t1	Regulatory subunit of β-1,3-glucan synthase complex	
β-1,6-glucan	*Skn1 /Kre6*	TM11_g4026.t1	Required for β-1,6-glucan biosynthesis, glucolsyltransferase	Yes
	Big1-like	TM11_g912.t1	Required for β-1,6-glucan biosynthesis	
	Kre9-like	TM11_g138.t1	Involved in β-1,6-glucan assembly	
N- and *O*- linked glycans	*Och1*	TM11_g1900.t1	*N*-glycan biosynthesis, initiation-specific α-1,6-mannosyltransferase	Yes[e]
	Och2	TM11_g3705.t1	*N*-glycan biosynthesis, initiation-specific α-1,6-mannosyltransferase	
	Och3	TM11_g612.t1[c]	*N*-glycan biosynthesis, initiation-specific α-1,6-mannosyltransferase	
	Och4/ Hoc1	TM11_g5232.t1	*N*-glycan biosynthesis, initiation-specific α-1,6-mannosyltransferase	
	Mnn9	TM11_g1214.t1	N-glycan biosynthesis, mannan polymerase complex subunit, Anp1 family protein	
	Anp1	TM11_g1427.t1	N-glycan biosynthesis, mannan polymerase complex subunit, Anp1 family protein	
	Anp2	TM11_g2471.t1	N-glycan biosynthesis, mannan polymerase complex subunit, Anp1 family protein	
	Gmh5	TM11_g326.t1	alpha-1,2-galactosyltransferase	
	Gmh6	TM11_g3737.t1	alpha-1,2-galactosyltransferase	
	Gmh4	TM11_g4039.t1	alpha-1,2-galactosyltransferase	
	Mnn5	TM11_g1566.t1	MNN1/MNT family mannosyltransferase	
	Mnn21	TM11_g3549.t1	Mnn2 mannosyltransferase family (N-glycan outer chain synthesis)	
	Mnn22	TM11_g4252.t1	Mnn2 mannosyltransferase family (N-glycan outer chain synthesis)	
	Mnn23	TM11_g209.t1	Mnn2 mannosyltransferase family (N-glycan outer chain synthesis)	
	Pmt1	TM11_g2967.t1	PMT gene family	
	Pmt2	TM11_g1995.t1	PMT gene family	
	Pmt3	TM11_g5433.t1	PMT gene family	
	Omh1	TM11_g5559.t1	KRE2/MNT1 mannosyltransferase family	
	Omh2	TM11_g166.t1	KRE2/MNT1 mannosyltransferase family	
	Omh3	TM11_g236.t1	KRE2/MNT1 mannosyltransferase family	
	Omh4	TM11_g4977.t1	KRE2/MNT1 mannosyltransferase family	
	Omh5	TM11_g990.t1	KRE2/MNT1 mannosyltransferase family	
