# Supplementary material for: A Novel Pathosystem With the Model Plant Arabidopsis thaliana for Defining the Molecular Basis of Taphrina Infections
Source: Environ Microbiol Rep. 2025 Jun 10;17(3):e70118. doi: 10.1111/1758-2229.70118 (PMC12152203; doi:10.1111/1758-2229.70118)
Supplement: Supplementary file 25 — TABLE S11. Promotor‐RUBY line immunity marker genes. [file EMI4-17-e70118-s008.pdf]

Table S11. Promotor-RUBY line immunity marker genes.

Gene	AGI code	Responsive to	References
ZAT12	AT5G59820	Biotic and abiotic stress, reactive oxygen species (ROS)	Lim *et al*., 2019
WRKY75	AT5G13080	Multiple treatments including *Botrytis cinerea,* senescence, ozone, salicylic acid	Chen *et al*., 2020; Guo *et al*., 2017; Vuorinen *et al*., 2021
WRKY40	AT1G80840	Flagellin (Flg22) treatment	Birkenbihl *et al*., 2016
LURP1	AT2G14560	Infection with *Hyaloperonospora arabidopsidis*	Knoth and Eulgem, 2008
JAZ10	AT5G13220	Methyl-jasmonate and wounding	Lehmann *et al*., 2020, Mishra *et al*., 2022

References:

Lim, S. *et al*. 2019. Quantitative ROS bioreporters: A robust toolkit for studying biological roles of ROS in response to abiotic and biotic stresses. *Physiologia Plantarum*, 165, 356-368.

Chen, L. *et al*. 2020. The transcription factor WRKY75 positively regulates jasmonate-mediated plant defense to necrotrophic fungal pathogens. *Journal of Experimental Botany*, 72, 1473-1489.

Guo, P. *et al*. 2017. A tripartite amplification loop involving the transcription factor WRKY75, salicylic acid, and reactive oxygen species accelerates leaf senescence. *The Plant Cell*, 29, 2854-2870.

Vuorinen *et al*. 2021. Dissecting contrasts in cell death, hormone, and defense signaling in response to *Botrytis cinerea* and reactive oxygen species. *Molecular Plant-Microbe Interactions*, 34, 75-87.

Birkenbihl, R. P. *et al*. 2016. Induced genome-wide binding of three *Arabidopsis* WRKY transcription factors during early MAMP-triggered immunity. *The Plant Cell*, 29, 20-38.

Knoth, C. and Eulgem, T. 2008. The oomycete response gene LURP1 is required for defense against *Hyaloperonospora parasitica* in *Arabidopsis thaliana*. *The Plant Journal*, 55, 53-64.

Lehmann, S. *et al*. 2020. Novel markers for high-throughput protoplast-based analyses of phytohormone signaling. *PLOS ONE*, 15, e0234154.

Mishra, D. K. *et al*. 2022. Identification and validation of the wound and insect bite early inducible promoter from *Arabidopsis thaliana*. 3 *Biotech*, 12, 74.
